# Gold(I)-catalysed one-pot synthesis of chromans using allylic alcohols and phenols

**DOI:** 10.3762/bjoc.9.209

**Published:** 2013-09-04

**Authors:** Eloi Coutant, Paul C Young, Graeme Barker, Ai-Lan Lee

**Affiliations:** 1 Institute of Chemical Sciences, Heriot-Watt University, Edinburgh, EH14 1LP, United Kingdom

**Keywords:** allylic alcohols, chromans, Friedel–Crafts, gold catalysis, heterocycles

## Abstract

A gold(I)-catalysed reaction of allylic alcohols and phenols produces chromans regioselectively via a one-pot Friedel–Crafts allylation/intramolecular hydroalkoxylation sequence. The reaction is mild, practical and tolerant of a wide variety of substituents on the phenol.

## Introduction

Chromans (dihydrobenzopyrans) are important and ubiquitous structural motifs found in a variety of important biologically active natural products such as vitamin E and flavanoids [1–5]. One approach towards chromans [6–12], which is biosynthetically inspired, is the Friedel–Crafts allylation [13] of phenols followed by cyclisation of the allylated phenol intermediate (via hydroalkoxylation). Initially, traditional allylating reagents such as allylic acetates were employed in Friedel–Crafts allylations [14], but more recently, there has been a distinct drive towards utilising more environmentally benign allylic alcohols (via a direct dehydrative coupling strategy) [15–16]. To this end, the use of molybdenum catalyst CpMoCl(CO)_3_ together with an oxidant, *o*-chloranil, has been documented to catalyse the reaction of allylic alcohols with phenols to form chromans [17–18]. Strong and superacids have also been utilised in the synthesis of chroman-containing targets [19–21]. Nevertheless, there are still a few drawbacks with these methods, for example, they usually require a large excess of substrate (e.g. ~30-fold excess), and in the case of acid catalysis, also poor yields when the phenol is not *para*-substituted. Therefore, it would be desirable to have a milder method which is compatible with a wide range of substituted phenols.

**Scheme 1:**
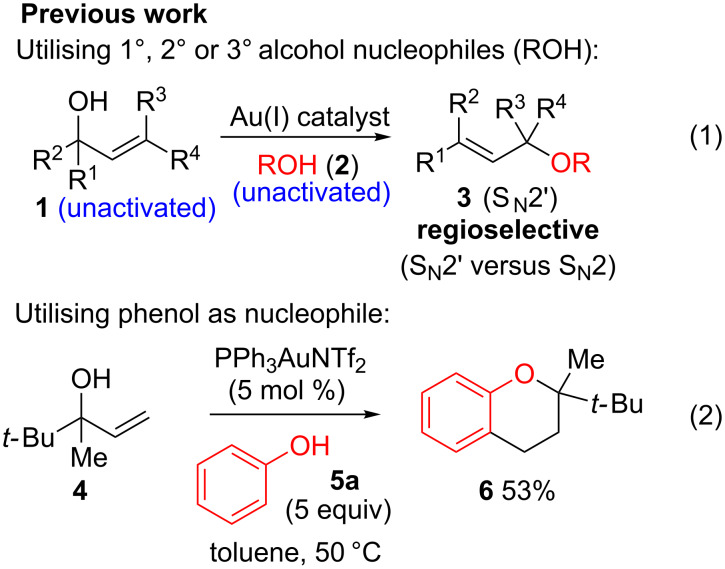
Previous work on direct allylic etherification of allylic alcohols.

As part of our continued interest in developing new gold-catalysed [22–41] reactions [42–51], we have recently shown that gold(I) can catalyse a direct allylic etherification [52–59] of unactivated alcohols **2** with unactivated allylic alcohols **1** (Scheme 1, reaction 1) [60–61]. The reaction is mild, regioselective, and produces only water as a byproduct. During our studies, a wide range of primary, secondary and tertiary alcohols were successfully employed as nucleophiles [61–72], but our one attempt employing a phenol **5** as a nucleophile surprisingly produced chroman **6** instead (Scheme 1, reaction 2). Although gold(III)-catalysed Friedel–Crafts allylation of phenols has been reported by Chan and co-workers [73], there have been no reports on the direct synthesis of chromans [74] using gold catalysis [75] with phenols and allylic alcohols prior to our example shown in Scheme 1.

Since the reaction is very practical: distilled solvents and inert air atmosphere are *not* required, and the chroman is formed directly in a mild one-pot procedure with only water as the byproduct, we decided to investigate the chroman-forming reaction in more detail, beyond the sole example previously reported (Scheme 1, reaction 2). In this paper, we present our further studies on this one-pot chroman synthesis: improving the yield of the desired chroman and exploring the substrate scope.

## Results and Discussion

To commence our studies, we first investigated the possibility of lowering the reaction temperature and used equivalents of phenol nucleophile. Suspecting that the moderate yield of **6** in Scheme 1 is due to the slight volatility of **6**, an allylic alcohol **7** with a higher molecular weight was chosen as the model substrate in order to avoid any issues of volatility with the chroman products. As shown in Table 1, the standard conditions (50 °C, 5 equiv phenol **5**) pleasingly provide a good 65% yield of the desired chroman **8** (Table 1, entry 1). Reducing the temperature is unfortunately detrimental to the formation of chroman: 40 °C gives a lower 59% yield of **8**, as well as Friedel–Crafts allylation products **9** and **10**, whereas 30 °C provides no chroman **8** at all, instead yielding only Friedel–Crafts products **9** and **10** (Table 1, entries 2 and 3). The *ortho*- and *para*-Friedel–Crafts products **9** and **10** are formed via formal S_N_2' regioselectivity and **9** is presumably the intermediate towards chroman **8** (vide infra). Thus, the higher temperature is clearly necessary to force the in situ cyclisation of **9** to **8**.

**Table 1:** Initial temperature and equivalents screens, and control reactions.

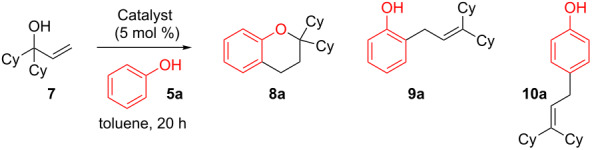						

Entry	Equiv **5**	Temp. (°C)	Catalyst	**8** (%)	**9** (%)	**10** (%)

1	5	50	PPh_3_AuNTf_2_	65^a^	-	–
2	5	40	PPh_3_AuNTf_2_	59^b^	10^b^	20^b^
3	5	30	PPh_3_AuNTf_2_	–	66^b^	27^b^
4	2	50	PPh_3_AuNTf_2_	–	63^b^	22^b^
5	1	50	PPh_3_AuNTf_2_	–	50^b^	19^b^
6	3	60	PPh_3_AuNTf_2_	63^b^	–	–
7	4	60	PPh_3_AuNTf_2_	60^b^	–	–
8	5	50	No catalyst	–	–	–
9	5	50	HNTf_2_	21^a^	–	–
10	5	50	AgNTf_2_	–	70^b^	25^b^

^a^Isolated yield. ^b^Yield obtained using ^1^H NMR analysis with 1,2,4,5-tetrachloro-3-nitrobenzene as internal standard.

Next, lower equivalents of phenol **5** were investigated. Unfortunately, dropping the equivalents of phenol also appears to be detrimental to chroman formation: only Friedel–Crafts products **9** and **10** are observed with 2 or 1 equivalents of phenol (Table 1, entries 4 and 5). Pleasingly however, lower equivalents of phenol are tolerated if the temperature is increased to 60 °C (Table 1, entries 6 and 7).

In order to ascertain if the gold(I) catalyst is really necessary for the formation of chroman **8**, several control reactions were carried out (Table 1, entries 8–10). Firstly, no reaction is observed in the absence of a catalyst (Table 1, entry 8). The Brønsted acid catalyst HNTf_2_ does form chroman **8**, but in a poorer isolated yield (21%, Table 1, entry 9) and the silver salt [76] AgNTf_2_ as a catalyst does not provide any **8**, yielding only **9** and **10** (Table 1, entry 10). The former is consistent with literature reports that Brønsted acid-catalysed reactions give poor yields when the phenol is not *para*-substituted [19]. Therefore, it seems that the gold(I) catalyst is most efficient in catalysing the one-pot formation of chroman **8**.

With these results in hand, a phenol screen was carried out next (Table 2). Initially, the same conditions that were best for the formation of **8a** were used (50 °C, 5 equiv **5a**, Table 2, entry 1) with *p*-cresol (**5b**), but these conditions only produced the Friedel–Crafts intermediate **9b** (Table 2, entry 2). Pleasingly, when the temperature was raised to 60 °C, the desired chroman **8b** is successfully formed in 57% yield (Table 2, entry 3). Therefore, 60 °C was adopted as the new general conditions temperature. Additionally, it was found that the equivalents of phenol **5** can be lowered to 2 equivalents in some cases at this higher temperature (Table 2, entries 4, 7, 8 and 10). In contrast to the Brønsted acid procedure [19], the substitution position has limited effect on the efficiency of the gold-catalysed reaction, with *p*-cresol (**5b**), *m*-cresol (**5c**) and *o*-cresol (**5d**) all forming the desired chromans **8b**–**d** in decent to good yields (Table 2, entries 3–5). The regioselectivity of the *meta* isomer is unsurprisingly poor (1:1 of **8c**:**8c'**), and when both *meta*-positions are substituted (**5e**) the reaction proceeds to **8e** well (Table 2, entry 6). A phenol with an electron-donating substituent **5f** provides **8f** in a good 71% yield (Table 2, entry 7) and one with an electron-withdrawing substituent **5g** pleasingly also produces chroman **8g** in a reasonable 54% yield (Table 2, entry 8). *p*-Bromo-substituted phenol **5h** successfully yields chroman **8h** which provides a handle for further functionalisation (Table 2, entry 9).

**Table 2:** Phenol nucleophile scope.

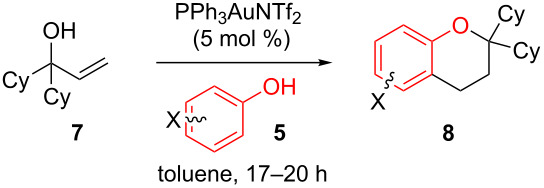						

Entry	Equiv **5**	Temp. (°C)	Time (h)	Phenol **5**	Product	Yield (%)^a^

1	5	50	19	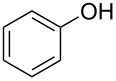 **5a**	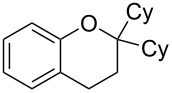 **8a**	64
2	5	50	64	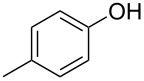 **5b**	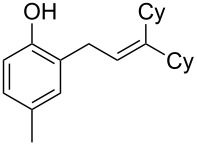 **9b**	N/D^b^
3	5	60	18	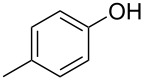 **5b**	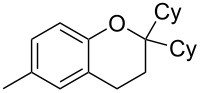 **8b**	57
4	2	60	17	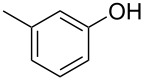 **5c**	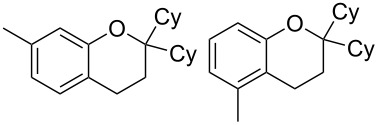 **8c** and **8c'**	71 (~1:1 **8c**:**8c'**)
5	5	60	18	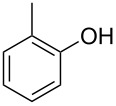 **5d**	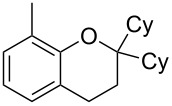 **8d**	69
6	5	60	17	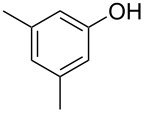 **5e**	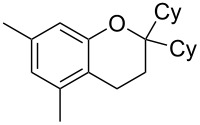 **8e**	63
7	2	60	17	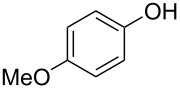 **5f**	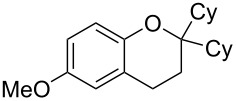 **8f**	71
8	2	60	18	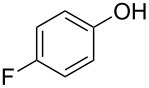 **5g**	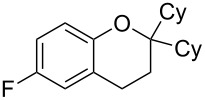 **8g**	54
9	5	60	17	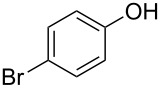 **5h**	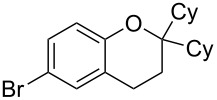 **8h**	58
10	2	60	17	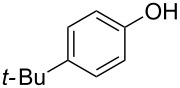 **5i**	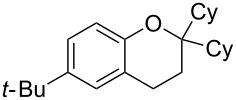 **8i**	57
11	5	70	43	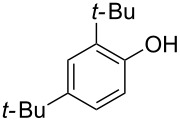 **5j**	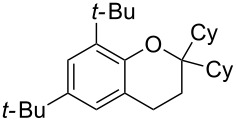 **8j**	83
12^c^	5	70, 48 h; 80, 17 h	65	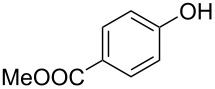 **5k**	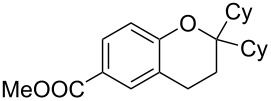 **8k**	69
13^c,d^	5	90	65	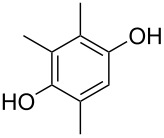 **5l**	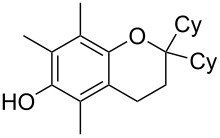 **8l**	83

^a^Isolated yield. ^b^Not determined. ^c^Solvent: dioxane. ^d^Reaction carried out in sealed tube.

Next, the effect of a larger substituent was probed (Table 2, entries 10 and 11). More hindered 2,4-di-*tert*-butylphenol (**5j**, Table 2, entry 11) requires a higher temperature (70 °C) and twice the reaction time (43 h) to go to completion compared to 4-*tert*-buylphenol (**5i**, Table 2, entry 10). Although phenol **5j** exhibits the slowest reactivity of the phenols screened, it was chosen as the model phenol in the next allylic alcohol substrate screen (Table 3) since the extra molecular weight from the *t*-Bu substituents should reduce any volatility issues with the chroman products [77].

At this point, we observed that the general procedure does not work if the phenol reactant is insoluble in toluene, such as **5k**. However, a simple change of solvent from toluene to dioxane provides the desired chroman **8k** (Table 2, entry 12), although slightly higher temperatures (70–80 °C) and a longer reaction time (65 h) are required in this polar solvent to push the reaction to completion.

**Figure 1:**
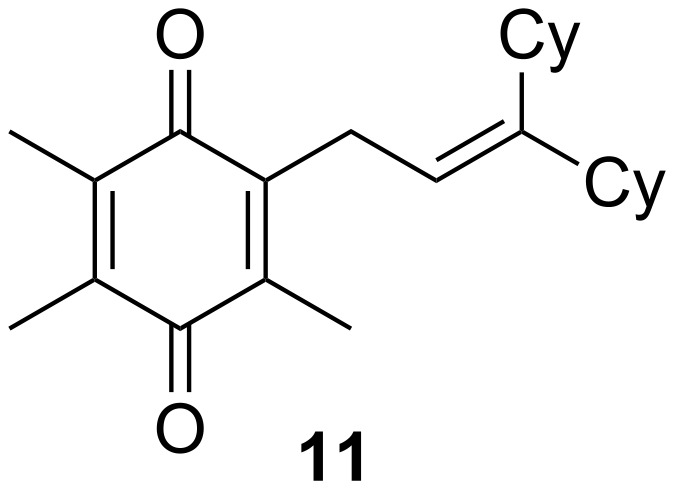
Initial side product with TMHQ.

Finally, to show the synthetic utility of this procedure, a hydroquinone (trimethylhydroquinone TMHQ (**5l**)) was also evaluated, as TMHQ is commonly used towards the synthesis of vitamin E and its analogues [17,21]. For solubility issues, dioxane is used as the solvent. Initially, an oxidised side product **11** (Figure 1, formed by auto-oxidation of the Friedel–Crafts intermediate) is observed in 35% yield if the reaction is carried out in air (80 °C), resulting in a low 45% yield of **8l**. When the reaction vessel is flushed with argon, the yield of **8l** improves to 69%, but ultimately carrying out the reaction at 90 °C in a sealed tube provides a much better yield of 83% (Table 2, entry 13).

Next, the allylic alcohol scope was investigated (Table 3). Firstly, going from more hindered Cy substituents (**7**) to less hindered *n*-hexyl substituents (**12**) allows the reaction to work smoothly at a lower temperature of 60 °C (Table 3. entries 1 and 2). Hindered **4** works equally well but requires extended reaction times (67 h) to achieve a good 83% yield (Table 3, entry 3). Less hindered **13** as well as **14** work smoothly to form **8o** and spirocyclic chroman **8p** (Table 3, entries 4 and 5). An aromatic substituent is also well tolerated (Table 3, entry 6). Next, the effect of substitution along the alkene was investigated (Table 3, entries 7–10). Substitution at the γ-position (**16** and **17**) seems to be tolerated, forming chromans **8r** and **8s** respectively albeit in moderate yields (Table 3, entries 7 and 8). Substitution at the β-position, however, is not tolerated: the reaction stops at the Friedel–Crafts stage (**9t**), and is reluctant to undergo further cyclisation to the desired chroman (Table 3, entry 9). Having investigated a series of tertiary allylic alcohols in entries 1–9, we next looked at selected primary and secondary allylic alcohols (Table 3, entries 10–12). The γ,γ-disubstituted primary allylic alcohol **19** forms the chroman **8o** efficiently (Table 3, entry 10), which is the same product as from the tertiary allylic alcohol substrate **13** in entry 4. This implies that **19** undergoes the Friedel–Crafts allylation via opposite regioselectivity (a formal S_N_2 instead of S_N_2' observed in all other examples so far in Table 2 and Table 3) to form **9**, followed by cyclisation to form the observed **8o**. Using a γ,γ-disubstituted secondary allylic alcohol **20** also forms chroman **8u** via an initial S_N_2 Friedel–Crafts regioselectivity, thus γ,γ-disubstitution on the alkene appears to be responsible for the switch in regioselectivity (Table 3, entry 11). This implies that the initial Friedel–Crafts allylation goes via Markovnikov selectivity. The unsubstituted secondary allylic alcohol **21**, however, produces only the Friedel–Crafts allylation product **9v** and is reluctant to undergo cyclisation to the chroman (Table 3, entry 12) even under more forcing conditions (80 °C, 65 h).

**Table 3:** Allylic alcohol scope.

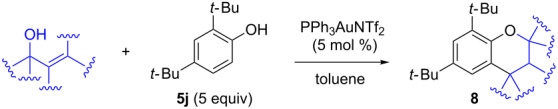					

Entry	Time (h)	Temp. (°C)	Allylic alcohol	Product	Yield (%)^a^

1	43	70	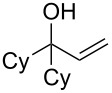 **7**	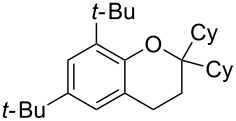 **8j**	83
2	43	60	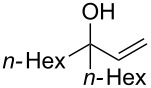 **12**	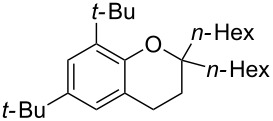 **8m**	69
3	67	60	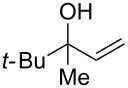 **4**	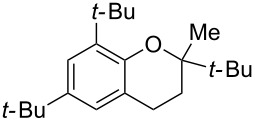 **8n**	83
4	42	60	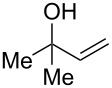 **13**	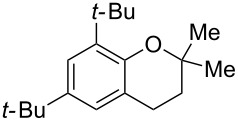 **8o**	64
5	41	60	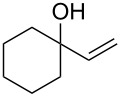 **14**	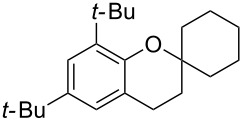 **8p**	61
6	41	70	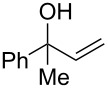 **15**	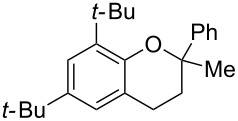 **8q**	66
7	43	70	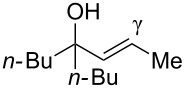 **16**	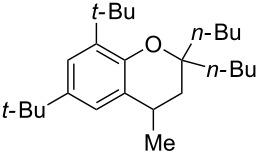 **8r**	45
8	47	70	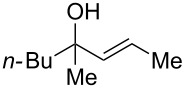 **17**	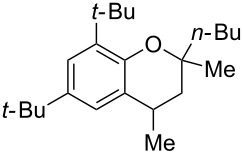 **8s**	48^b^
9	42	60	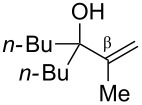 **18**	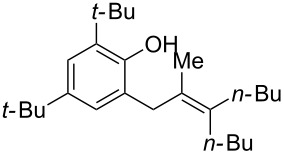 **9t**	35
10	42	60	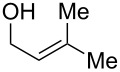 **19**	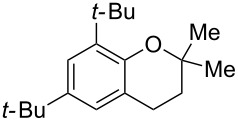 **8o**	78
11	41	70	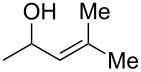 **20**	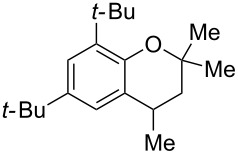 **8u**	51
12^c^	41	60	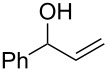 **21**	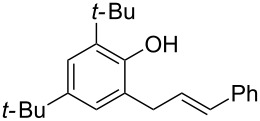 **9v**	74

^a^Isolated yield. ^b^Approximately 2:1 d.r. ^c^Heating at 80 °C for 65 h still only gives **9v** and no desired chroman.

**Scheme 2:**
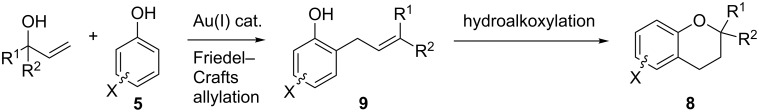
Proposed pathway.

Since **9** is always observed as a precursor towards **8** (i.e. at lower temperatures, shorter reactions times or when the reaction is analysed before completion), the most likely mechanism is the expected gold(I)-catalysed Friedel–Crafts allylation of the phenol (via Markovnikov regioselectivity), with allylic alcohol [15,73], followed by cyclisation of the intermediate **9** via hydroalkoxylation to form chroman **8** (Scheme 2) [13,15–16]. Chan and co-workers have previously proposed that the Friedel–Crafts mechanism could involve the activation of the allylic alcohol by the gold catalyst to turn the hydroxy group into a better leaving group [73]. The observed regioselectivities is then due to the subsequent attack at the less hindered position of this presumed activated intermediate [73,78].

**Scheme 3:**
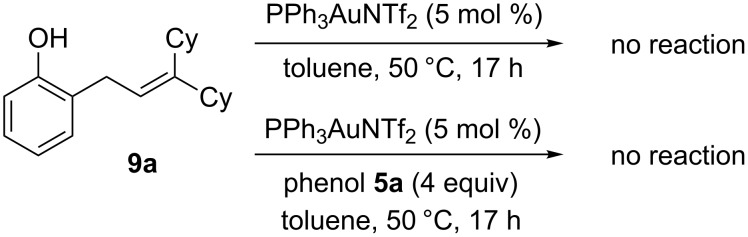
Control reactions.

Our subsequent investigations with the Friedel–Crafts intermediate **9a** suggests that the second hydroalkoxylation step is not (or not solely) gold catalysed (Scheme 3). When isolated **9a** is resubjected to the reaction conditions with or without additional phenol (**5a**), no cyclisation to the chroman **8a** occurs (Scheme 3). Thus, the second cyclisation step is most likely Brønsted acid catalysed (or acid and gold(I) co-catalysed) [79], where the H^+^ required is being released in situ during the first Friedel–Crafts step to form **9**. This would explain why **9** readily cyclises to chroman **8** in situ, but is reluctant to do so when it is isolated before being resubjected to more gold(I) catalyst, as in Scheme 3. Nevertheless, simply using the equivalent Brønsted acid HNTf_2_ is not as efficient as using gold(I), as shown in Table 1, entry 9.

**Scheme 4:**
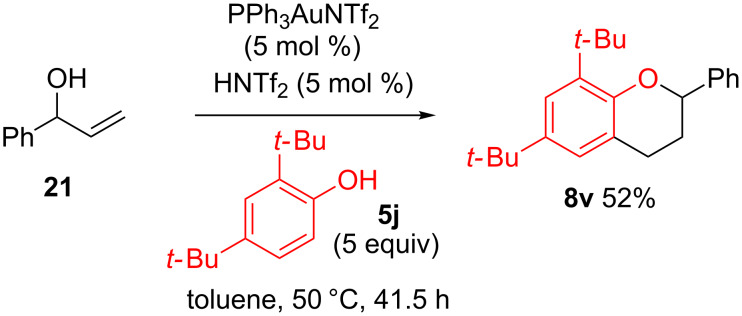
Reaction of **21** with added Brønsted acid co-catalyst.

If the hydroalkoxylation step is indeed H^+^ catalysed, addition of a Brønsted acid co-catalyst could force the chroman formation from substrates such as **21**, which do not undergo the second hydroalkoxylation step under just gold(I)-catalysed conditions (Table 3, entry 12). Indeed, the reaction of **21** with **5j** successfully produces the desired chroman **8v** in 52% yield when HNTf_2_ is added as a co-catalyst (Scheme 4), further suggesting that the hydroalkoxylation step is most likely Brønsted acid catalysed or co-catalysed.

**Scheme 5:**
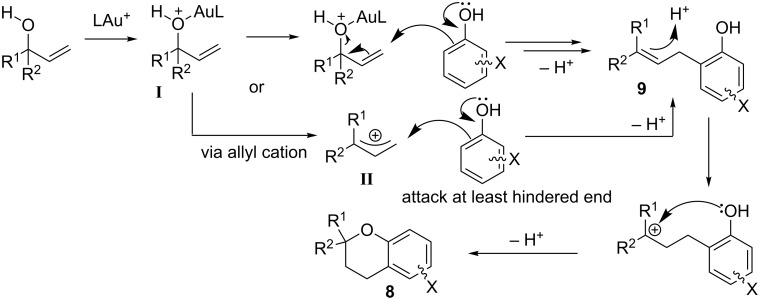
Suggested mechanism.

The suggested mechanism is presented in Scheme 5. Gold(I) catalysts are known to coordinate to alcohols [80], in this case turning the hydroxy group into a better leaving group (**I**), as previously suggested by Chan [73]. Attack at the less hindered position could occur either directly on **I** [S_N_2' shown, but in the case of γ,γ-disubstituted substrates (e.g. **19** and **20**), this will occur via S_N_2] or via an allylic cation intermediate **II**. The intermediate **9** subsequently undergoes an acid-catalysed hydroalkoxylation to produce the desired chroman **8**. Active catalyst LAu^+^ is presumably regenerated by protonolysis of LAuOH [81].

## Conclusion

In conclusion, a simple one-pot procedure towards chromans is described via gold(I)-catalysed reaction of readily accessible phenols with allylic alcohols. This one-pot procedure involves a regioselective Friedel–Crafts allylation followed by cyclisation via hydroalkoxylation to form the chromans in good yields. At lower temperatures or shorter reaction times, the Friedel–Crafts allylation intermediates are usually observed. The reaction works with *ortho*-, *meta*- and *para*-substituents as well as electron donating and withdrawing substituents on the phenol, and hydroquinones (Table 2). A variety of allylic alcohol substrates work well, although substitution on the alkene is only tolerated at the γ-position, and not the β-position (Table 3). The procedure is mild, practically simple and regioselective. We therefore believe that it should find utility as a convenient method towards the synthesis of chroman targets.

## Experimental

**General procedure:** The gold-catalysed reactions were all carried out in 1 dram screw-cap vials without the need for distilled solvents or inert atmosphere, unless otherwise stated. PPh_3_AuNTf_2_ (5 mol %) was added to a toluene solution (0.386 M) of allylic alcohol (1 equiv) and phenol (2 or 5 equiv). The reaction mixture was allowed to stir at 50–70 °C until the reaction is complete (16–67 h). The reaction was then filtered through a plug of silica (eluent: neat diethyl ether). The filtrate was concentrated under reduced pressure, and ^1^H NMR analysis of the crude mixture was used to determine the conversion to chroman **8**. The crude material was then purified by flash column chromatography. [Note: If the starting materials are insoluble in toluene, dioxane is used as the solvent instead and the reaction temperature increased to 70–80 °C.]

## Supporting Information

File 1 Full experimental procedures, characterisation for all new compounds and copies of ^1^H and ^13^C NMR spectra.

## References

[1] Schneider C (2005). Mol Nutr Food Res.

[2] Brogden P J, Gabbutt C D, Hepworth J D, Katrizky A (1984). Pyrans and Fused Pyrans. Comprehensive Heterocyclic Chemistry.

[3] Middleton E, Kandaswami C, Theoharides T C (2000). Pharmacol Rev.

[4] Ren W, Qiao Z, Wang H, Zhu L, Zhang L (2003). Med Res Rev.

[5] Shen H C (2009). Tetrahedron.

[R6] 6A related procedure towards chromans is the acid- or metal triflate-mediated cyclisation of phenols with 1,3-dienes. Using these methods, chromans and/or coumarans are obtained, depending on the structure of the 1,3-diene used. For selected recent examples, see references [7–10].

[7] Dang T T, Boeck F, Hintermann L (2011). J Org Chem.

[8] Youn S W (2007). Synlett.

[9] Youn S W, Eom J I (2006). J Org Chem.

[10] Adrio L A, Hii K K (2008). Chem Commun.

[11] Bienaymé H, Ancel J-E, Meilland P, Simonato J-P (2000). Tetrahedron Lett.

[12] Nguyen R V, Yao X, Li C J (2006). Org Lett.

[13] Rueping M, Nachtsheim B J (2010). Beilstein J Org Chem.

[14] Malkov A V, Davis S L, Baxendale I R, Mitchell W L, Kočovský P (1999). J Org Chem.

[15] Bandini M, Tragni M (2009). Org Biomol Chem.

[16] Kumar R, Van der Eycken E V (2013). Chem Soc Rev.

[17] Yamamoto Y, Itonaga K (2009). Org Lett.

[18] Malkov A V, Spoor P, Vinader V, Kočovský P (1999). J Org Chem.

[19] Ishino Y, Mihara M, Hayakawa N, Miyata T, Kaneko Y, Miyata T (2001). Synth Commun.

[20] Lee J H, Bang H B, Han S Y, Jun J-G (2007). Tetrahedron Lett.

[21] Hasegawa A, Ishihara K, Yamamoto H (2003). Angew Chem, Int Ed.

[22] Gorin D J, Toste F D (2007). Nature.

[23] Fürstner A, Davies P W (2007). Angew Chem, Int Ed.

[24] Li Z, Brouwer C, He C (2008). Chem Rev.

[25] Shen H C (2008). Tetrahedron.

[26] Shen H C (2008). Tetrahedron.

[27] Hashmi A S K (2007). Chem Rev.

[28] Marion N, Nolan S P (2008). Chem Soc Rev.

[29] Jiménez-Núñez E, Echavarren A M (2007). Chem Commun.

[30] Corma A, Leyva-Peréz A, Sabater M J (2011). Chem Rev.

[31] Bandini M (2011). Chem Soc Rev.

[32] Boorman T C, Larrosa I (2011). Chem Soc Rev.

[33] Hashmi A S K, Bührle M (2010). Aldrichimica Acta.

[34] Shapiro N D, Toste F D (2010). Synlett.

[35] Sengupta S, Shi X (2010). ChemCatChem.

[36] Bongers N, Krause N (2008). Angew Chem, Int Ed.

[37] Gorin D J, Sherry B D, Toste F D (2008). Chem Rev.

[38] Jiménez-Núñez E, Echavarren A M (2008). Chem Rev.

[39] Hashmi A S K, Hutchings G J (2006). Angew Chem, Int Ed.

[40] Rudolph M, Hashmi A S K (2012). Chem Soc Rev.

[41] Muzart J (2008). Tetrahedron.

[42] Bauer J T, Hadfield M S, Lee A-L (2008). Chem Commun.

[43] Hadfield M S, Lee A-L (2010). Org Lett.

[44] Hadfield M S, Bauer J T, Glen P E, Lee A-L (2010). Org Biomol Chem.

[45] Heuer-Jungemann A, McLaren R G, Hadfield M S, Lee A-L (2011). Tetrahedron.

[46] Hadfield M S, Lee A-L (2011). Chem Commun.

[47] Kilpin K J, Paul U S D, Lee A-L, Crowley J D (2011). Chem Commun.

[48] Young P C, Hadfield M S, Arrowsmith L, Macleod K M, Mudd R J, Jordan-Hore J A, Lee A-L (2012). Org Lett.

[49] Hadfield M S, Häller L J L, Lee A-L, Macgregor S A, O'Neill J A T, Watson A M (2012). Org Biomol Chem.

[50] Mudd R J, Young P C, Jordan-Hore J A, Rosair G M, Lee A-L (2012). J Org Chem.

[51] Young P C, Green S L J, Rosair G M, Lee A-L (2013). Dalton Trans.

[52] Aponick A, Biannic B, Jong M R (2010). Chem Commun.

[53] Aponick A, Biannic B (2011). Org Lett.

[54] Aponick A, Li C-Y, Biannic B (2008). Org Lett.

[55] Biannic B, Ghebreghiorgis T, Aponick A (2011). Beilstein J Org Chem.

[56] Bandini M, Monari M, Romaniello A, Tragni M (2010). Chem–Eur J.

[57] Unsworth W P, Stevens K, Lamont S G, Robertson J (2011). Chem Commun.

[58] Ghebreghiorgis T, Biannic B, Kirk B H, Ess D H, Aponick A (2012). J Am Chem Soc.

[59] Biannic B, Aponick A (2011). Eur J Org Chem.

[60] Young P C, Schopf N A, Lee A-L (2013). Chem Commun.

[61] Mukherjee P, Widenhoefer R A (2013). Chem–Eur J.

[R62] 62For an independent report using NHC gold(I) complexes, see reference [61]. For related reactions with *N*-nucleophiles instead, see references [63–67].

[63] Mukherjee P, Widenhoefer R A (2010). Org Lett.

[64] Ohshima T, Nakahara Y, Ipposhi J, Miyamoto Y, Mashima K (2011). Chem Commun.

[65] Mukherjee P, Widenhoefer R A (2011). Org Lett.

[66] Kothandaraman P, Foo S J, Chan P W H (2009). J Org Chem.

[67] Mukherjee P, Widenhoefer R A (2012). Angew Chem, Int Ed.

[68] Kothandaraman P, Mothe S R, Toh S S M, Chan P W H (2011). J Org Chem.

[69] Kothandaraman P, Huang C, Susanti D, Rao W, Chan P W H (2011). Chem–Eur J.

[70] Kothandaraman P, Rao W, Foo S J, Chan P W H (2010). Angew Chem, Int Ed.

[71] Rao W, Chan P W H (2008). Chem–Eur J.

[72] Chen Z, Zhang Y-X, Wang Y-H, Zhu L L, Liu H, Li X X, Guo L (2010). Org Lett.

[73] Rao W, Chan P W H (2008). Org Biomol Chem.

[74] Jean M, van de Weghe P (2011). Tetrahedron Lett.

[75] Rudolph M, Hashmi A S K (2011). Chem Commun.

[76] Wang D, Cai S, Sharma S, Jirak J, Thummanapelli S K, Akhmedov N G, Zhang H, Liu X, Peterson J L, Shi X (2012). J Am Chem Soc.

[R77] 77We have noted that the formation of chromans **8a**–**8j** from **7** is also accompanied by the formation of a rearrangement side-product in approximately 15–25% yields (see Supporting Information File 1 for details). The formation of this rearrangement side product is only ever observed with cyclohexyl substituents (i.e. **7**) and is not detected in any of the other allylic alcohols screened in Table 3. We have previously observed unusual behaviour in other substrates containing *bis*-cyclohexyl substituents in gold(I)-catalysed reactions, see reference [48].

[78] Georgy M, Boucard V, Campagne J-M (2005). J Am Chem Soc.

[79] Hashmi A S K (2007). Catal Today.

[80] Zhdanko A, Ströbele M, Maier M E (2012). Chem–Eur J.

[81] Gaillard S, Bosson J, Ramón R S, Nun P, Slawin A M Z, Nolan S P (2010). Chem–Eur J.

