# Supplementary material for: Gold(I)-catalysed one-pot synthesis of chromans using allylic alcohols and phenols
Source: Beilstein J Org Chem. 2013 Sep 4;9:1797–806. doi: 10.3762/bjoc.9.209 (PMC3778418; doi:10.3762/bjoc.9.209)
Supplement: File 1 — Full experimental procedures, characterisation for all new compounds and copies of 1H and 13C NMR spectra. [file Beilstein_J_Org_Chem-09-1797-s001.pdf]

Supporting Information

for

Gold(I)-catalysed one-pot synthesis of chromans using allylic alcohols and phenols

Eloi Coutant, Paul C. Young, Graeme Barker, Ai-Lan Lee*

Address: Institute of Chemical Sciences, Heriot-Watt University, Edinburgh, EH14 1LP, United Kingdom

Email: Ai-Lan Lee - A.Lee@hw.ac.uk

*Corresponding author

Full experimental procedures, characterisation for all new compounds and copies of ^{1}H and ^{13}C NMR spectra

Contents

General experimental section S3

Procedure, characterisation, ^{1}H and ^{13}C NMR spectra of synthesised compounds S4

General experimental section

^{1}H NMR spectra were recorded on Bruker AV 300 and AV 400 spectrometers at 300 and 400 MHz respectively. ^{13}C NMR spectra were recorded using the same spectrometers at 75 and 100 MHz respectively. Chemical shifts (δ in ppm) were referenced to tetramethylsilane (TMS) or to residual solvent peaks ($CDCl_3$ at δ_H 7.26). *J* values are given in Hz and s, d, dd, t, q, qn and m abbreviations correspond to singlet, doublet, doublet of doublet, triplet, quartet, quintet and multiplet. Mass spectra were obtained at the EPSRC National Mass Spectrometry Service Centre in Swansea. Infrared spectra were obtained on Perkin-Elmer Spectrum 100 FTIR Universal ATR Sampling Accessory, deposited neat or as a chloroform solution to a diamond/ZnSe plate. Flash column chromatography was carried out using Matrix silica gel 60 from Fisher Chemicals and TLC was performed using Merck silica gel 60 F_{254} precoated sheets and visualised by UV (254 nm) or stained by the use of aqueous acidic $KMnO_4$ or aqueous acidic ceric ammonium molybdate or iodine as appropriate. Petrol ether refers to petroleum ether (40–60 °C). Solvents were purchased from Fisher and used without further purification unless otherwise stated.

Gold catalysts were purchased from Sigma-Aldrich and used without further purification. All phenols **5** were purchased from Sigma-Aldrich or Fisher and used without further purification. Allylic alcohol **13** and **19** were purchased from Sigma-Aldrich. All other allylic alcohol substrates were prepared following known literature procedures. Allylic alcohols **4**, **7**, **12–15** were obtained following known literature procedure with Grignard addition to ketones/aldehydes [1,2]. **16**, **17**, **18** were prepared by *n*-BuLi addition to the corresponding enone [3]. **20** was prepared from reduction of the corresponding enone [4].

The gold(I)-catalysed reactions were carried out in 1 dram screw cap vials without the need for dry solvents or inert atmosphere, unless stated otherwise.

2,2-Dicyclohexylchroman **(8a)**

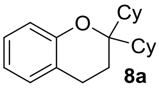

8a

1,1-Dicylohexylprop-2-en-1-ol (**7**, 20.5 mg, 92.2 µmol) and phenol **5a** (42.8 mg, 455 µmol) were dissolved in toluene (0.19 mL). PPh_3AuNTf_2 (as the 2:1 toluene adduct) (3.4 mg, 4.6 µmol) was added to the resulting solution. The reaction was allowed to stir at 50 °C for 19 hours. The reaction was then filtered over a plug of silica, using diethyl ether as eluent. The crude mixture was purified using column chromatography, using a gradient eluent system of neat hexane to 20:1 hexane:diethyl ether. Product **8a** was obtained as a colourless film (17.7 mg, 59.3 µmol, 64%).

ν_{max}/cm^{-1} 2923, 2852, 1582, 1488, 1452, 1255, 1242, 987, 907, 751, 731.

δ_H (300 MHz, $CDCl_3$) 7.11–6.98 (2H, m, Ar-H), 6.81 - 6.74 (2H, m, Ar-H), 2.67 (2H, t, *J* 6.7 Hz, CH_2), 1.87 (2H, t, *J* 6.7 Hz, CH_2), 1.95–1.57 (12H, m, Cy-H), 1.31–1.03 (10H, m, Cy-H).

δ_C (75 MHz, $CDCl_3$) 154.8 (C), 129.1 (CH), 127.3 (CH), 122.2 (C), 119.1 (CH), 117.2 (CH), 82.0 (C), 44.3 (CH), 28.3 (CH_2), 27.8 (CH_2), 27.5 (CH_2), 27.2 (CH_2), 27.0 (CH_2), 23.0 (CH_2), 22.2 (CH_2).

Found (APCI$^+$) $[M + H]^+$ 299.2369, $C_{21}H_{31}O$ requires 299.2369.

elchb002
1H 300.1MHz Job 22818 Coutant Eloi B002 CDCl3   25.1°C
Isolated dicyclohexyl chroman

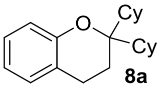

8a

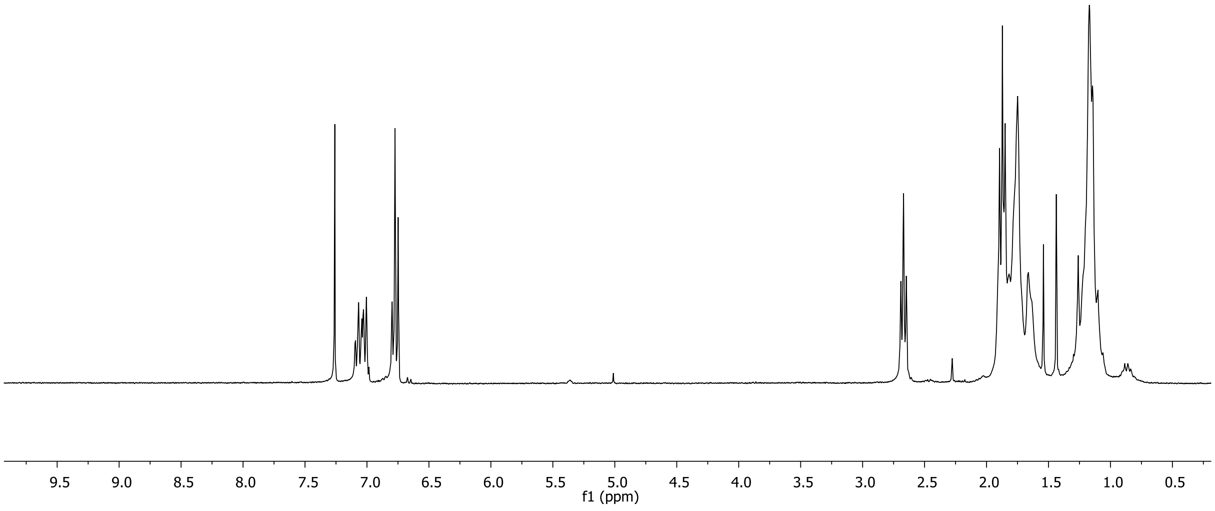

elccc002.1.fid
13C 75.5MHz Job 22846 Coutant Eloi C002 CDCl3   25.1°C   3 hours 1 min
Dicyclohexyl chroman, dried

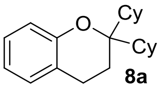

8a

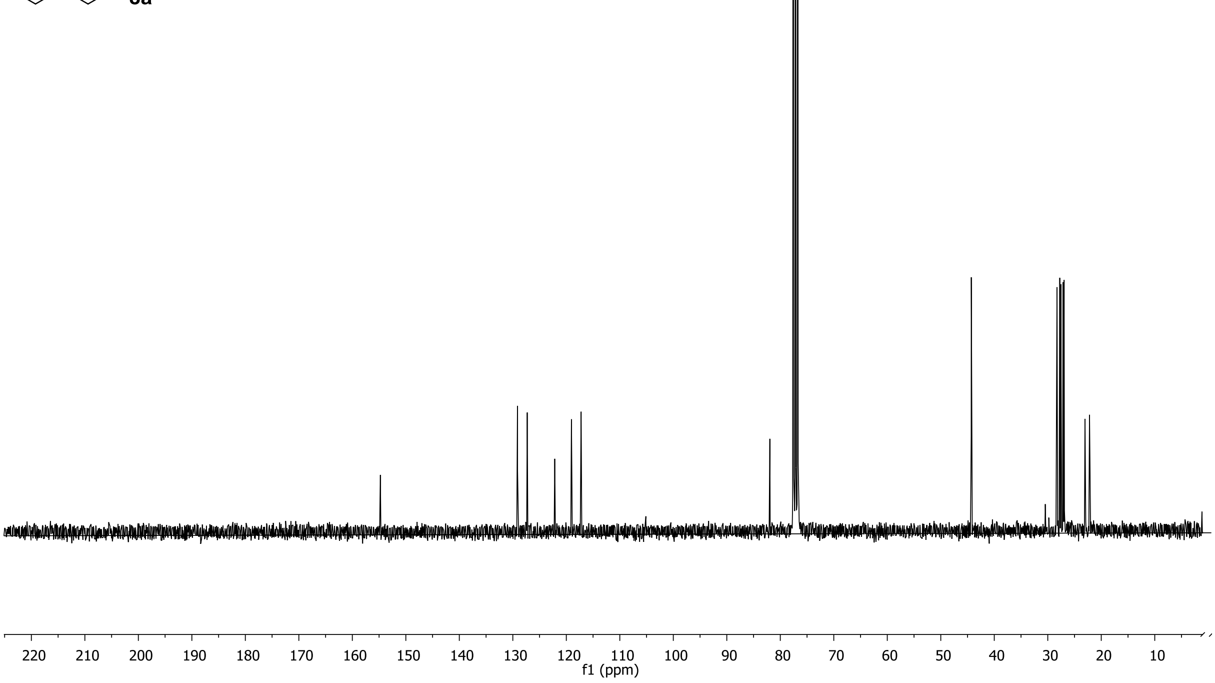

2,2-Dicyclohexyl-6-methylchroman (8b)

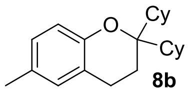

8b

1,1-Dicylohexylprop-2-en-1-ol (**7**, 15.5 mg, 69.7 µmol) and *p*-cresol (**5b**, 37.3 mg, 345 µmol) were dissolved in toluene (0.14 mL). PPh_3AuNTf_2 (as the 2:1 toluene adduct) (2.6 mg, 3.5 µmol) was added to the resulting solution. The reaction was allowed to stir at 60 °C for 18 hours. The reaction was then filtered over a plug of silica, using diethyl ether as eluent. The crude mixture was purified using column chromatography, using a gradient eluent system of neat hexane to 9:1 hexane:diethyl ether. Product **8b** was obtained as a white solid (12.4 mg, 39.7 µmol, 57%).

Mp: 99–103 °C.

ν_{max}/cm^{-1} 2921, 2851, 1497, 1448, 1302, 1255, 1243, 1217, 1142, 987, 946, 894, 877, 811, 756.

δ_H (300 MHz, $CDCl_3$) 6.90–6.82 (2H, m, Ar-H), 6.67 (1H, d, *J* 8.2 Hz, Ar-H), 2.63 (2H, t, *J* 6.7 Hz, CH_2), 2.25 (3H, s, CH_3), 1.85 (2H, t, *J* 6.7 Hz, CH_2), 1.93–1.58 (12H, m, Cy-H), 1.40–1.00 (10H, m, Cy-H).

δ_C (75 MHz, $CDCl_3$) 152.5 (C), 129.5 (CH), 128.1 (C), 128.0 (CH), 121.8 (C), 117.0 (CH), 81.7 (C), 44.3 (CH), 28.3 (CH_2), 27.8 (CH_2), 27.5 (CH_2), 27.2 (CH_2), 27.0 (CH_2), 23.1 (CH_2), 22.2 (CH_2), 20.7 (CH_3).

Found (APCI$^+$) $[M + H]^+$ 313.2526, $C_{22}H_{33}O$ requires 313.2526.

elchb023a
1H 300.1MHz Job 24033 Coutant Eloi B023A CDCl3 25.1°C
A fraction - DiCyAA + p-Cresol, 50C, over week-end

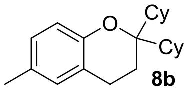

8b

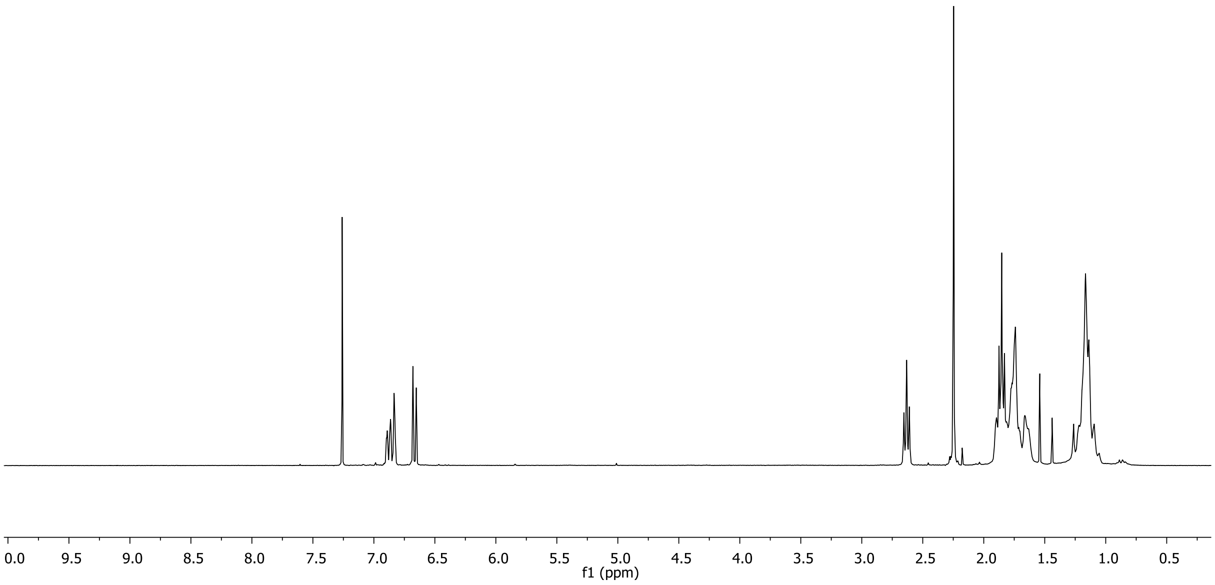

elccb023a
13C 75.5MHz Job 24072 Coutant Eloi B023A CDCl3 25.0°C 3 hours 1 min
A fraction - DiCyAA + p-Cresol, 50C, over week-end

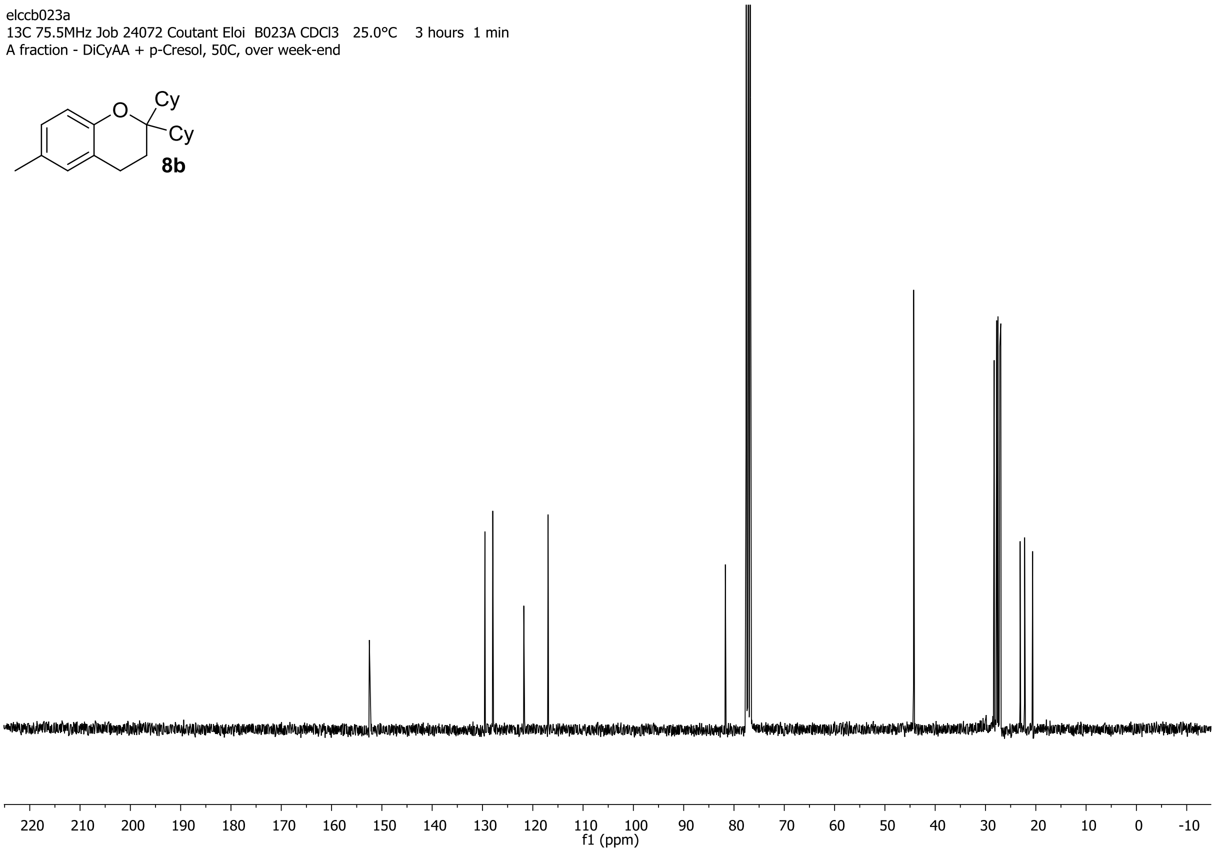

2,2-Dicyclohexyl-5-methylchroman and 2,2-dicyclohexyl-7-methylchroman **(8c and 8c')**

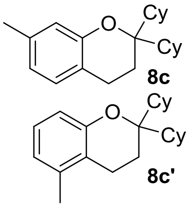

1,1-Dicylohexylprop-2-en-1-ol (**7**, 15.2 mg, 68.4 µmol) and *m*-cresol (**5c**, 14 µL, 125.2 µmol) were dissolved in toluene (0.14 mL). PPh_3AuNTf_2 (as the 2:1 toluene adduct) (2.5 mg, 3.4 µmol) was added to the resulting solution. The reaction was allowed to stir at 60°C for 17 hours. The reaction was then filtered over a plug of silica, using diethyl ether as eluent. 2,2-Dicyclohexyl-5-methylchroman **8c** and 2,2-dicyclohexyl-7-methylchroman **8c'** were isolated as a mixture of inseparable isomers by column chromatography, using a gradient eluent system of neat hexane to 9:1 hexane:diethyl ether. Product **8c** and **8c'** (approximately 1:1 mixture) were obtained as an off-white oil (15.1 mg, 48.3 µmol, 71%).

ν_{max}/cm^{-1} 2922, 2852, 1466, 1449, 1265, 1247, 1215, 1083, 1063, 1051, 753.

δ_H (300 MHz, $CDCl_3$) 6.99 (1H, t, *J* 8.0 Hz, Ar-$\underline{H}$), 6.91 (1H, d, *J* 8.0 Hz, Ar-$\underline{H}$), 6.70–6.55 (4H, m, Ar-$\underline{H}$), 2.63 (2H, t, *J* 6.7 Hz, $C\underline{H_2}$), 2.53 (2H, t, *J* 6.7 Hz, $C\underline{H_2}$), 2.26 (3H, s, $C\underline{H_3}$), 2.22 (3H, s, $C\underline{H_3}$), 1.88 (2H, t, *J* 6.7 Hz, $C\underline{H_2}$), 1.93–1.57 (24H, m, Cy-$\underline{H}$), 1.85 (2H, d, *J* 6.7 Hz, $C\underline{H_2}$), 1.29–1.03 (20H, m, Cy-$\underline{H}$).

δ_C (75 MHz, $CDCl_3$) 154.45 (C), 154.42 (C), 137.1 (C), 136.8 (C), 128.9 (CH), 126.7 (CH), 120.7 (C), 120.5 (CH), 120.0 (CH), 119.0 (C), 117.6 (CH), 115.1 (CH), 81.8 (C), 81.0 (C), 44.2 (CH), 43.9 (CH), 28.31 (CH_2), 28.29 (CH_2), 27.7 (CH_2), 27.5 (CH_2), 27.14 (CH_2), 27.12 (CH_2), 27.0 (CH_2), 23.0 (CH_2), 22.8 (CH_2), 21.8 (CH_2), 21.3 (CH_3), 19.7 (CH_2), 19.3 (CH_3).

Found (APCI$^+$) $[M + H]^+$ 313.2524, $C_{22}H_{33}O$ requires 313.2526.

elchb043b
1H 300.1MHz Job 25303 Coutant Eloi B043B CDCl3 18.7°C
B fraction - DiCyAA + 2eq m-Cresol, 60C, o/n

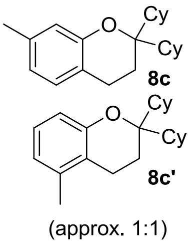

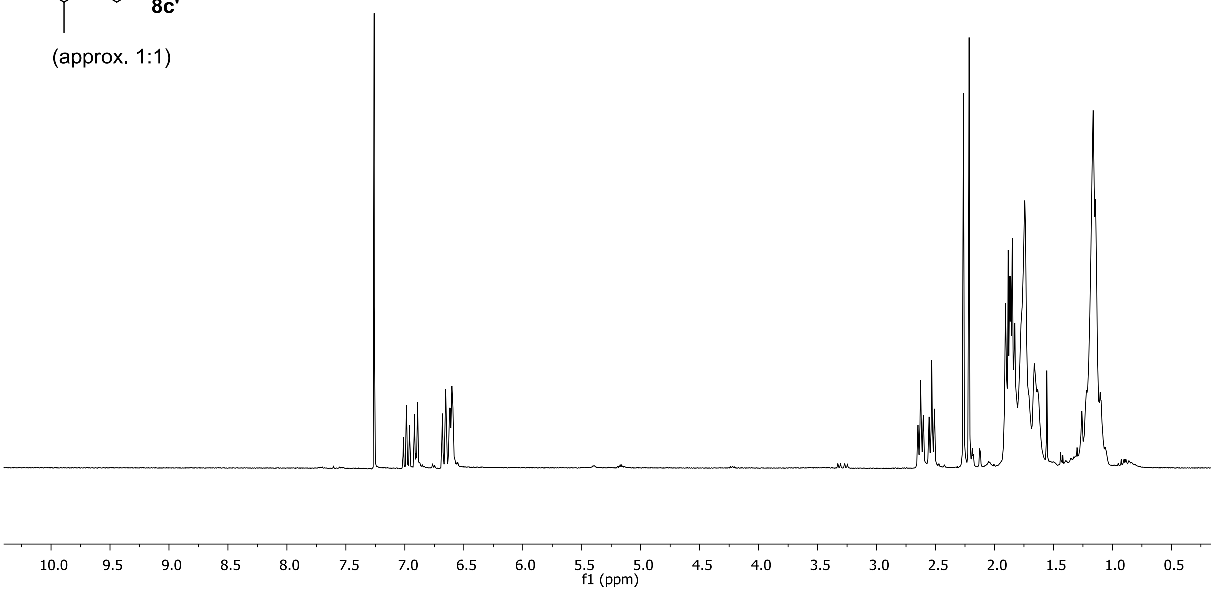

elccb043b
13C 75.5MHz Job 25331 Coutant Eloi B043B CDCl3 19.3°C 3 hours 1 min
Mixture of 2 chromans - DiCyAA + 2eq m-Cresol, 60C, o/n

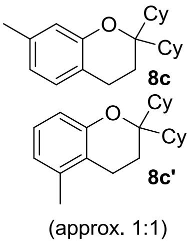

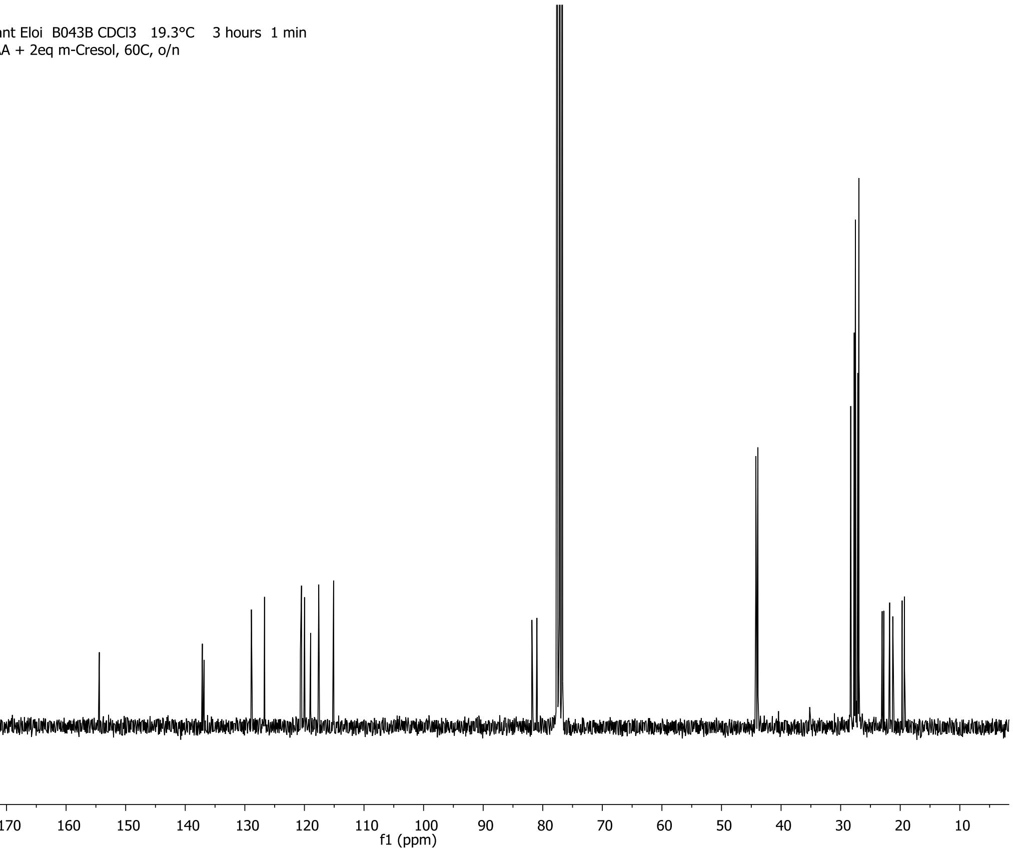

2,2-Dicyclohexyl-8-methylchroman (8d)

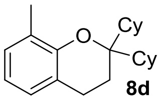

8d

1,1-Dicylohexylprop-2-en-1-ol (**7**, 15.1 mg, 67.9 µmol) and *o*-Cresol (**5d**, 36.6 mg, 338 µmol) were dissolved in toluene (0.14 mL). PPh_3AuNTf_2 (as the 2:1 toluene adduct) (2.7 mg, 3.7 µmol) was added to the resulting solution. The reaction was allowed to stir at 60 °C for 18 hours. The reaction was then filtered over a plug of silica, using diethyl ether as eluent. The crude mixture was purified using column chromatography, using a gradient eluent system of neat hexane to 9:1 hexane:diethyl ether. Product **8d** was obtained as a pink oil (14.7 mg, 47.0 µmol, 69%).

ν_{max}/cm^{-1} 2924, 2853, 1467, 1449, 1263, 1216, 1263, 1216, 1084, 987, 943, 907, 754, 731.

δ_H (300 MHz, $CDCl_3$) 6.94 (1H, d, *J* 7.4 Hz, Ar-H), 6.87 (1H, d, *J* 7.4 Hz, Ar-H), 6.69 (1H, t, *J* 7.4 Hz, Ar-H), 2.66 (2H, t, *J* 6.7 Hz, CH_2), 2.17 (3H, s, CH_3), 1.92–1.50 (12H, m, Cy-H), 1.87 (2H, t, *J* 6.7 Hz, CH_2), 1.32–1.02 (10H, m, Cy-H).

δ_C (75 MHz, $CDCl_3$) 152.6 (C), 128.3 (CH), 126.7 (CH), 126.2 (C), 121.3 (C), 118.4 (CH), 81.8 (C), 44.3 (CH), 28.3 (CH_2), 27.9 (CH_2), 27.6 (CH_2), 27.2 (CH_2), 27.0 (CH_2), 23.3 (CH_2), 22.3 (CH_2), 16.31 (CH_3).

Found ($APCI^+$) $[M + H]^+$ 313.2527, $C_{22}H_{33}O$ requires 313.2526.

elcha039a
1H 300.1MHz Job 25020 Coutant Eloi A039A CDCl3 25.0°C
A fraction - DiCyAA + 5eq o-Cresol, 60C, o/n

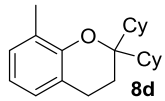

8d

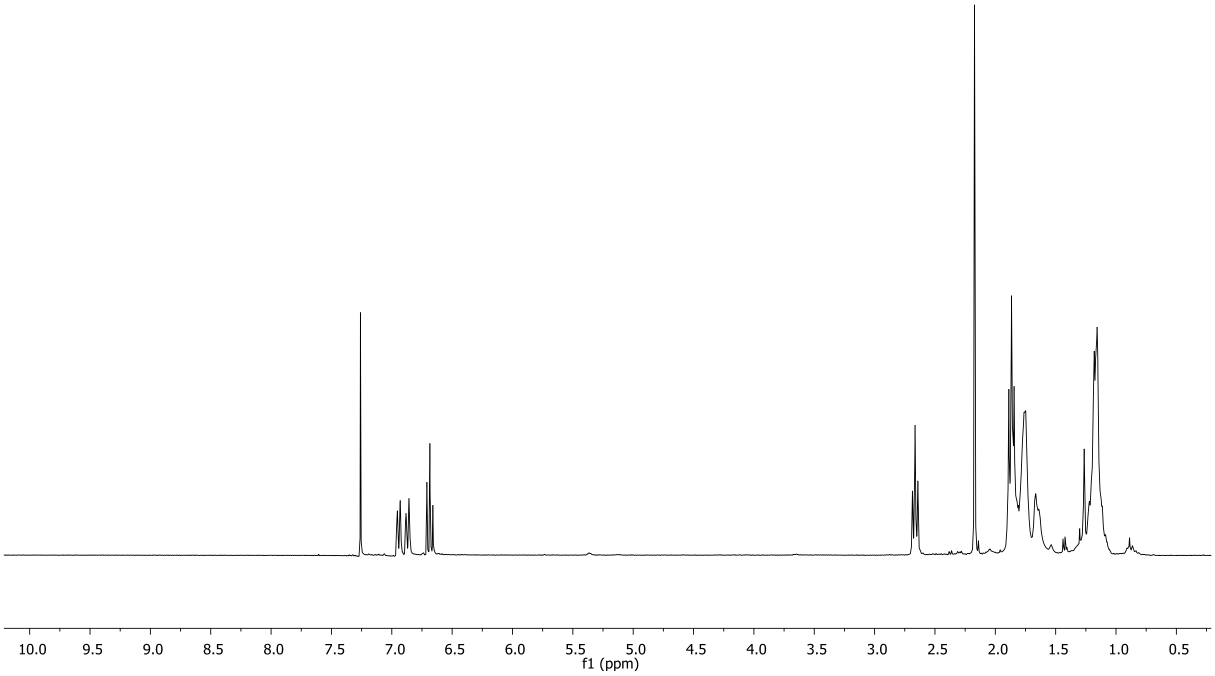

elccc039a
13C 75.5MHz Job 25152 Coutant Eloi C039A CDCl3 21.5°C 3 hours 1 min
2,2-dicyclohexyl-8-methylchroman

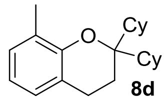

8d

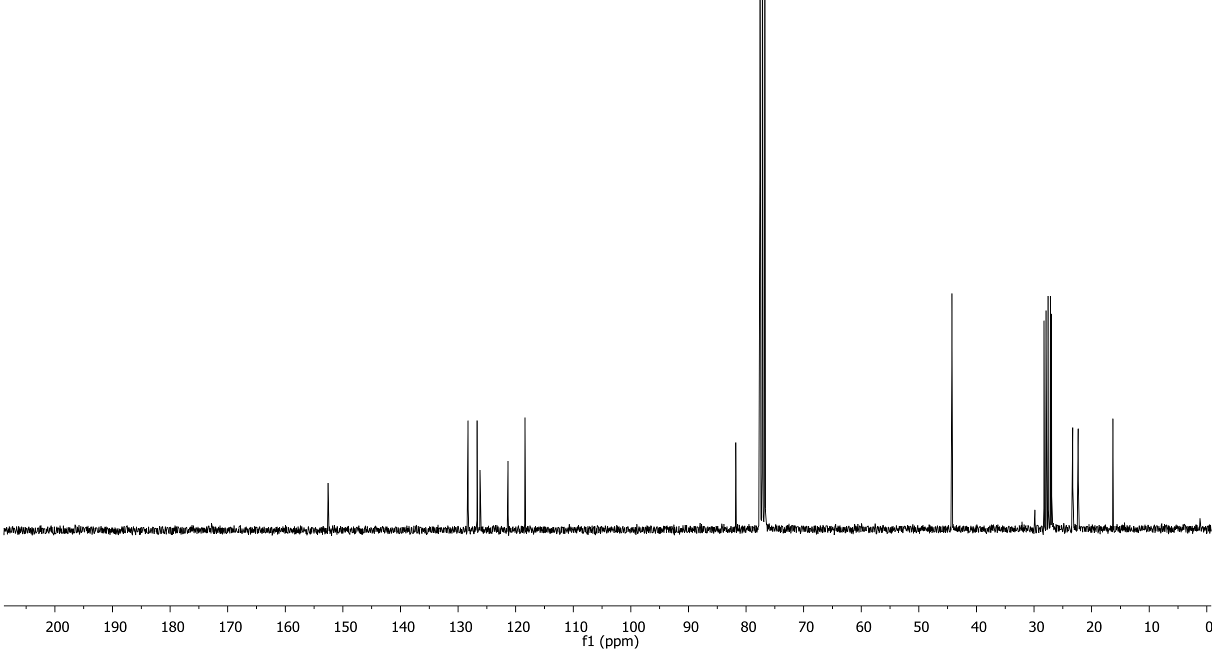

2,2-Dicyclohexyl-5,7-dimethylchroman (8e)

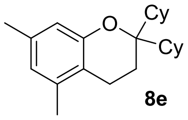

8e

1,1-Dicylohexylprop-2-en-1-ol (**7**, 15.0 mg, 67.5 μmol) and 3,5-dimethylphenol (**5e**, 41.2 mg, 337 μmol) were dissolved in toluene (0.14 mL). PPh_3AuNTf_2 (as the 2:1 toluene adduct) (2.6 mg, 3.5 μmol) was added to the resulting solution. The reaction was allowed to stir at 60 °C for 17 hours. The reaction was then filtered over a plug of silica, using diethyl ether as eluent. The crude mixture was purified using column chromatography, using a gradient eluent system of neat hexane to 9:1 hexane:diethyl ether. Product **8e** was obtained as a pink oil (13.8 mg, 42.3 μmol, 63%).

ν_{max}/cm^{-1} 2922, 2852, 1578, 1449, 1316, 1301, 1140, 1083, 1070, 836, 755.

δ_H (300 MHz, $CDCl_3$) 6.51 (1H, s, Ar-H), 6.48 (1H, s, Ar-H), 2.50 (2H, t, *J* 6.7 Hz, CH_2), 2.23 (3H, s, CH_3), 2.18 (3H, s, CH_3), 1.87 (2H, t, *J* 6.7 Hz, CH_2), 1.94–1.50 (12H, m, Cy-H), 1.31–1.00 (10H, m, Cy-H).

δ_C (75 MHz, $CDCl_3$) 154.3 (C), 136.6 (C), 136.5 (C), 121.6 (CH), 117.5 (C), 115.4 (CH), 80.9 (C), 44.0 (CH), 28.4 (CH_2), 27.8 (CH_2), 27.6 (CH_2), 27.2 (CH_2), 27.0 (CH_2), 22.9 (CH_2), 21.1 (CH_3), 19.4 (CH_2), 19.2 (CH_3).

Found (APCI$^+$) $[M + H]^+$ 327.2684, $C_{23}H_{35}O$ requires 327.2682.

elchb031a
1H 300.1MHz Job 24599 Coutant Eloi B031A CDCl3 25.1°C
A fraction - DiCyAA + 5eq m,m-DiMePhenol, 60C, o/n

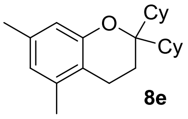

8e

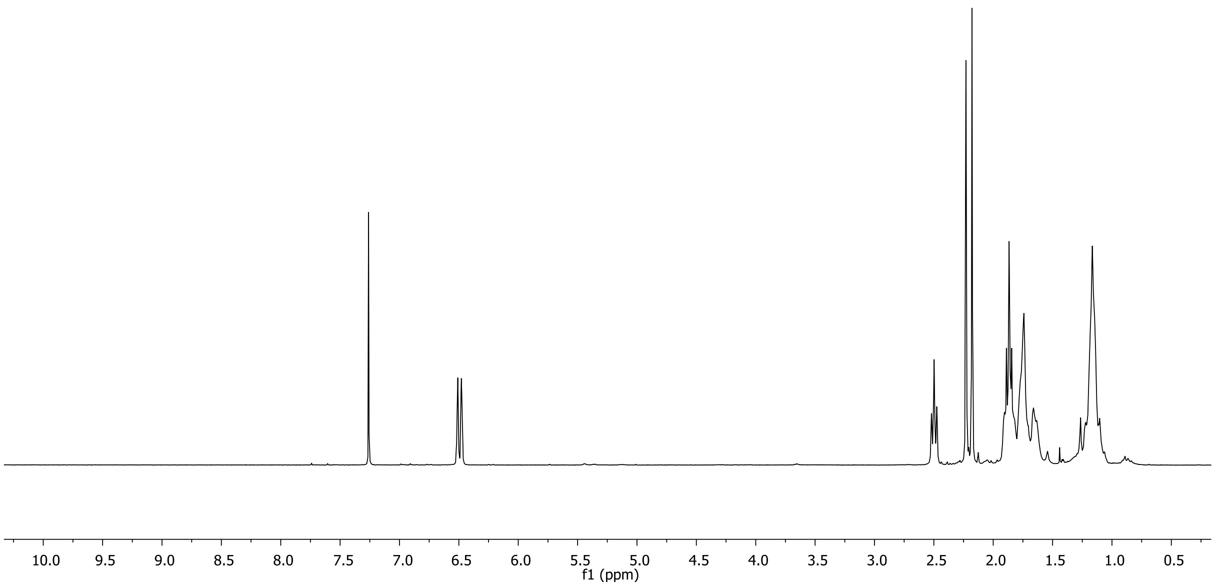

elccb031a
13C 75.5MHz Job 24612 Coutant Eloi B031A CDCl3 25.0°C 3 hours 1 min
A fraction - DiCyAA + 5eq m,m-DiMePhenol, 60C, o/n

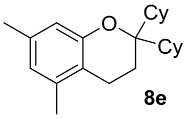

8e

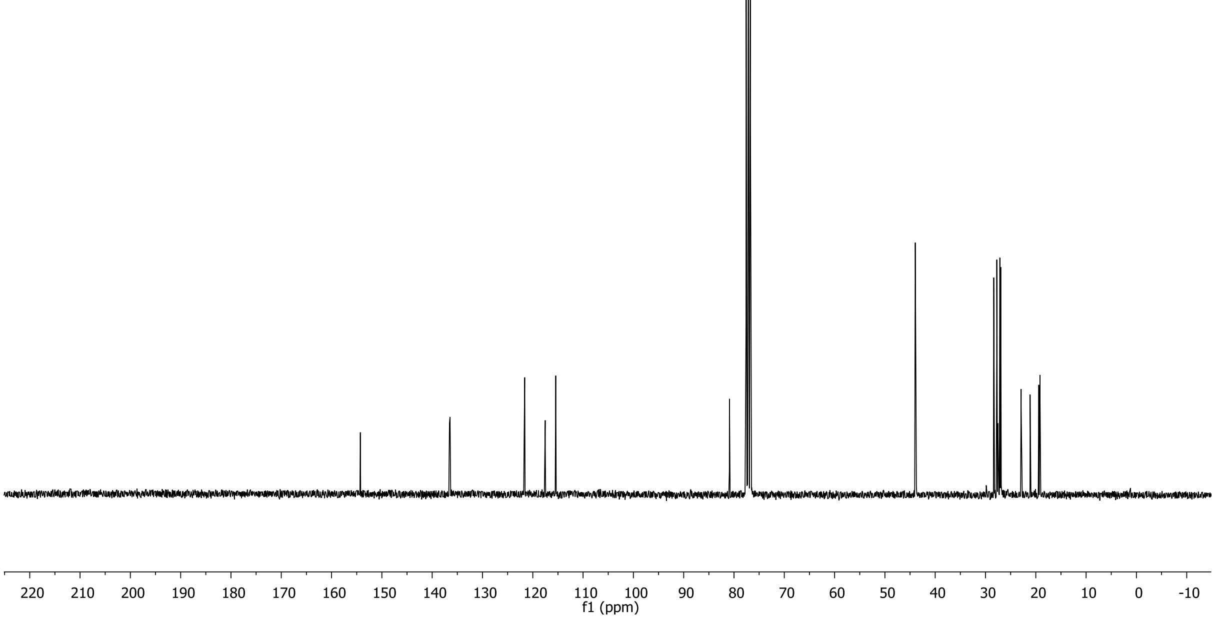

2,2-Dicyclohexyl-6-methoxychroman (8f)

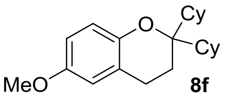
8f

1,1-Dicylohexylprop-2-en-1-ol (**7**, 15.1 mg, 67.9 µmol) and 4-methoxyphenol (**5f**, 17.0 mg, 137 µmol) were dissolved in toluene (0.14 mL). PPh_3AuNTf_2 (as the 2:1 toluene adduct) (2.5 mg, 3.4 µmol) was added to the resulting solution. The reaction was allowed to stir at 60 °C for 17 hours. The reaction was then filtered over a plug of silica, using diethyl ether as eluent. The crude mixture was purified using column chromatography, using a gradient eluent system of neat hexane to 9:1 hexane:diethyl ether. Product **8f** was obtained as a yellow solid (15.9 mg, 48.4 µmol, 71%).

Mp: 95-99 °C.

ν_{max}/cm^{-1} 2924, 2852, 1495, 1449, 1235, 1216, 1148, 1047, 895.

δ_H (300 MHz, $CDCl_3$) 6.69–6.65 (2H, m, Ar-H), 6.59–6.56 (1H, m, Ar-H), 3.74 (3H, s, O-CH$_3$), 2.65 (2H, t, *J* 6.7 Hz, CH$_2$), 1.85 (2H, t, *J* 6.7 Hz, CH$_2$), 1.93–1.57 (12H, m, Cy-H), 1.28–1.01 (10H, m, Cy-H).

δ_C (75 MHz, $CDCl_3$) 152.5 (C), 148.7 (C), 122.7 (C), 117.7 (CH), 113.8 (CH), 113.3 (CH), 81.6 (C), 55.8 (CH_3), 44.2 (CH), 28.3 (CH_2), 27.8 (CH_2), 27.5 (CH_2), 27.2 (CH_2), 26.9 (CH_2), 23.1 (CH_2), 22.6 (CH_2).

Found (APCI$^+$) $[M + H]^+$ 329.2475, $C_{22}H_{33}O_2$ requires 329.2476.

elchb041a
1H 300.1MHz Job 25122 Coutant Eloi B041A CDCl3 25.0°C
100-75:1 fraction - DiCyAA + 5eq p-methoxyphenol, 60C, o/n

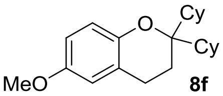

8f

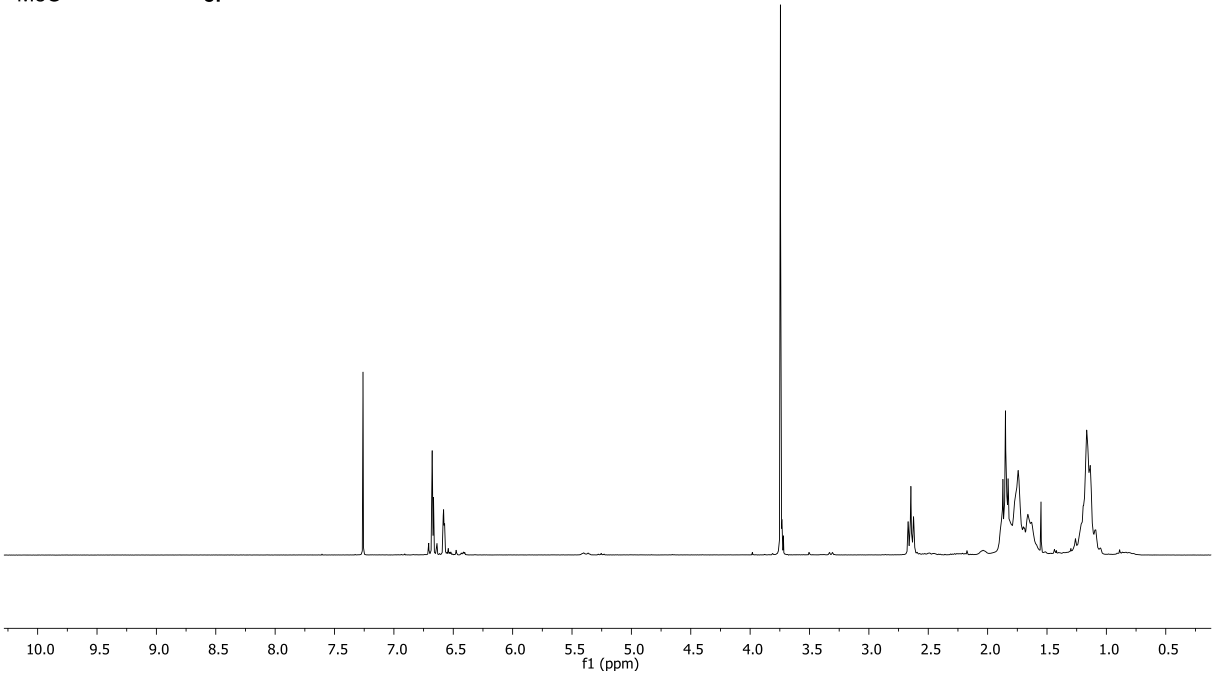

elccb041a
13C 75.5MHz Job 25158 Coutant Eloi B041A CDCl3 19.6°C 3 hours 1 min
2,2-dicychlohexyl-6-methoxychroman

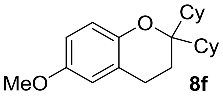

8f

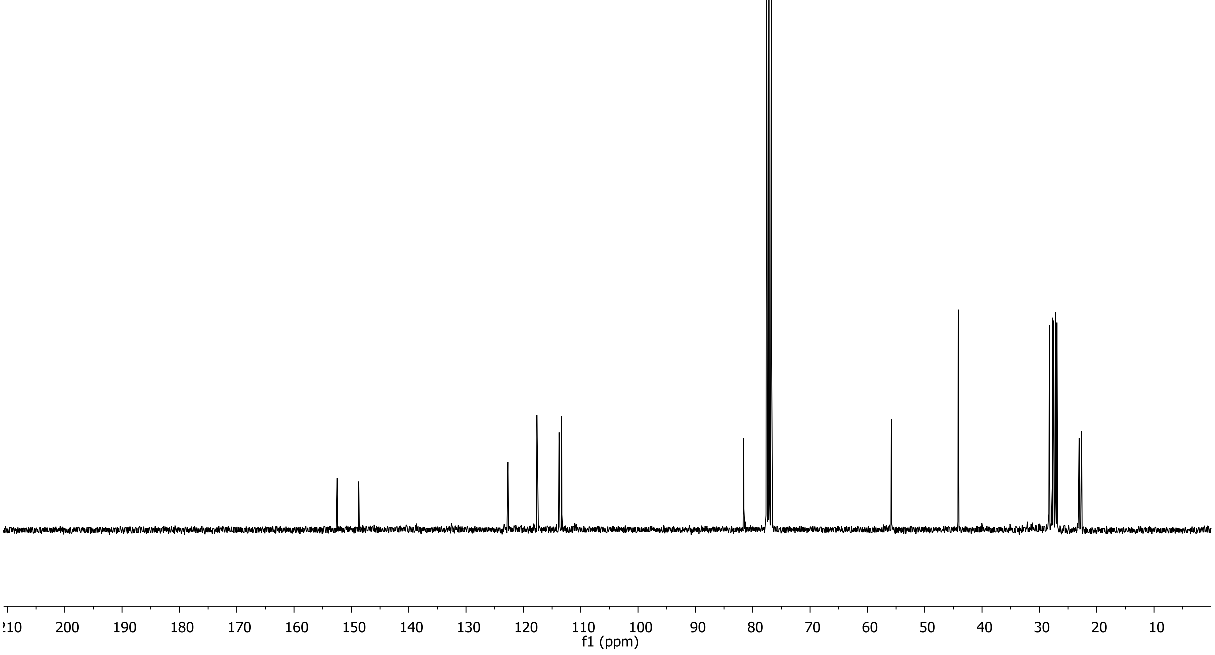

2,2-Dicyclohexyl-6-fluorochroman **(8g)**

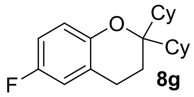

8g

1,1-Dicylohexylprop-2-en-1-ol (**7**, 15.1 mg, 67.9 µmol) and 4-fluorophenol (**5g**, 15.1 mg, 135 µmol) were dissolved in toluene (0.14 mL). PPh_3AuNTf_2 (as the 2:1 toluene adduct) (2.5 mg, 3.4 µmol) was added to the resulting solution. The reaction was allowed to stir at 60 °C for 18 hours. The reaction was then filtered over a plug of silica, using diethyl ether as eluent. The crude mixture was purified using column chromatography, using a gradient eluent system of neat hexane to 9:1 hexane:diethyl ether. Product **8g** was obtained as a yellowish amorphous solid (11.5 mg, 36.3 µmol, 54%).

ν_{max}/cm^{-1} 2923, 2852, 1490, 1449, 1433, 1254, 1232, 1212, 1138, 945, 895, 861, 808, 758, 727.

δ_H (300 MHz, $CDCl_3$) 6.84–6.62 (3H, m, Ar-H), 2.64 (2H, t, *J* 6.7 Hz, C$\underline{H_2}$), 1.90–1.56 (12H, m, Cy-$\underline{H}$), 1.85 (2H, t, *J* 6.7 Hz, C$\underline{H_2}$), 1.31–1.01 (10H, m, Cy-$\underline{H}$).

δ_C (75 MHz, $CDCl_3$) 156.2 (d, *J* 236.3 Hz, C), 150.7 (C), 123.2 (d, *J* 7.3 Hz, C), 117.8 (d, *J* 8.0 Hz, CH), 114.9 (d, *J* 22.3 Hz, CH), 113.9 (d, *J* 22.9 Hz, CH), 82.0 (C), 44.2 (CH), 28.2 (CH_2), 27.8 (CH_2), 27.5 (CH_2), 27.2 (CH_2), 26.9 (CH_2), 22.9 (CH_2), 22.5 (CH_2).

δ_F (282 MHz, $CDCl_3$) -126.12 (td, *J* 8.5, 5.1 Hz).

Found (APCI$^+$) $[M + H]^+$ 317.2278, $C_{21}H_{30}FO$ requires 317.2275.

elchb047b
1H 300.1MHz Job 25414 Coutant Eloi B047B CDCl3 25.0°C
B fraction, fluorochroman? - DiCyAA + 2eq 4-fluorophenol,60C,o/n

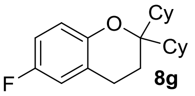

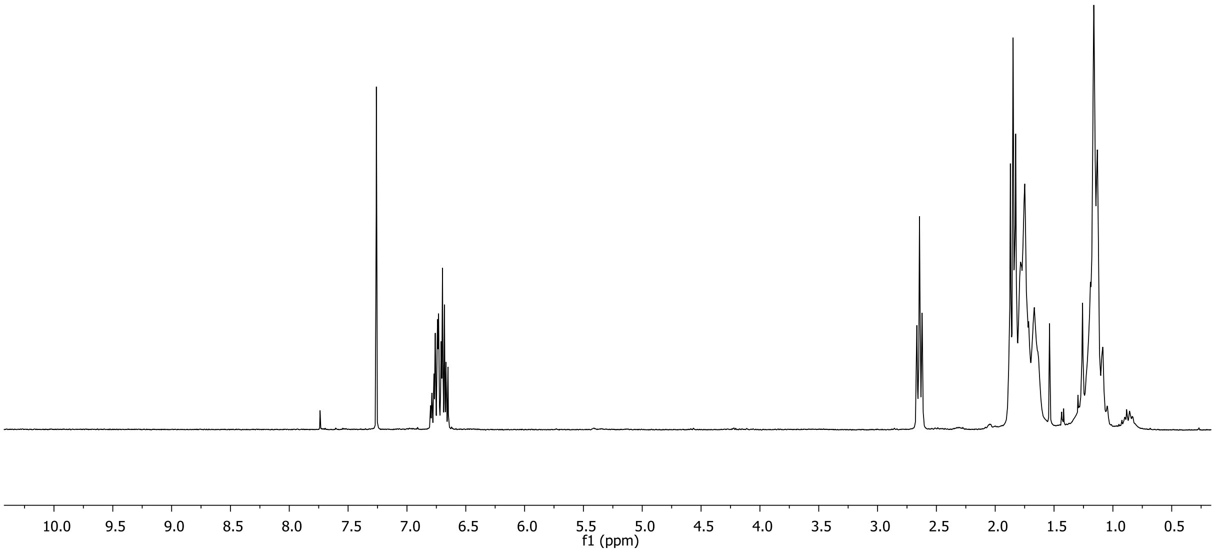

elccb047b
13C 75.5MHz Job 25434 Coutant Eloi B047B CDCl3 25.0°C 3 hours 1 min
B fraction, fluorochroman? - DiCyAA + 2eq 4-fluorophenol,60C,o/n

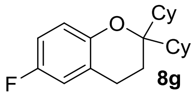

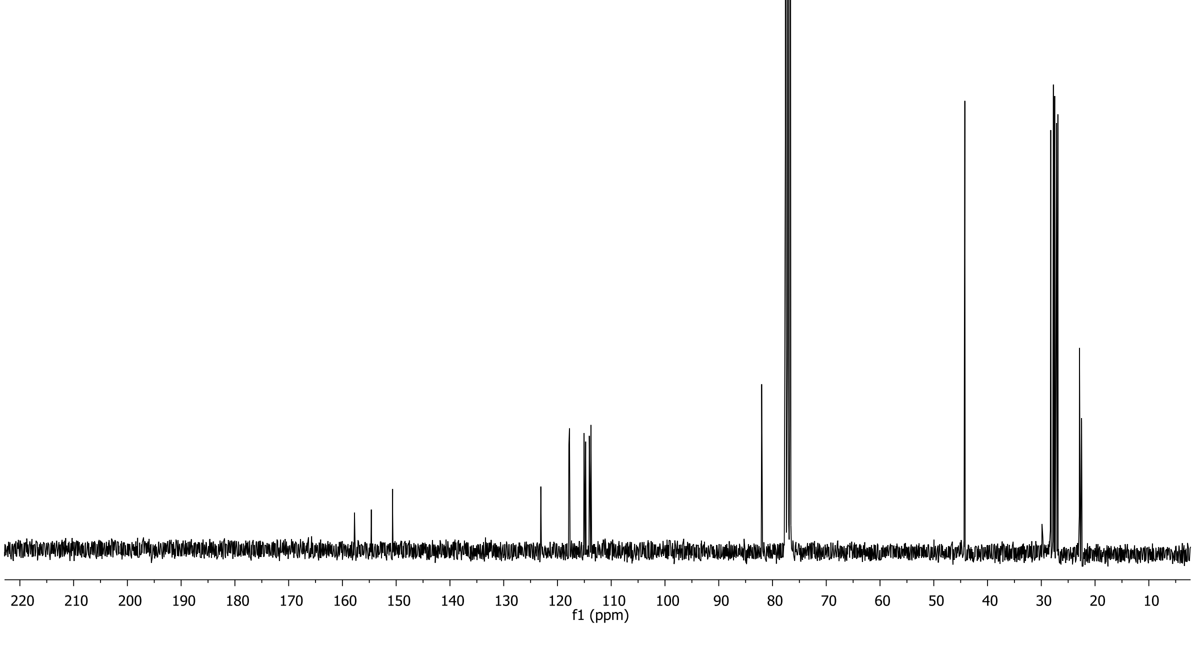

elcfb047b
19F 282.4MHz Job 26893 Coutant Eloi B047B CDCl3   25.0°C   0hours 1min
2,2-dicyclohexyl-6-fluorochroman

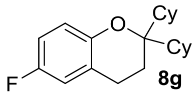

8g

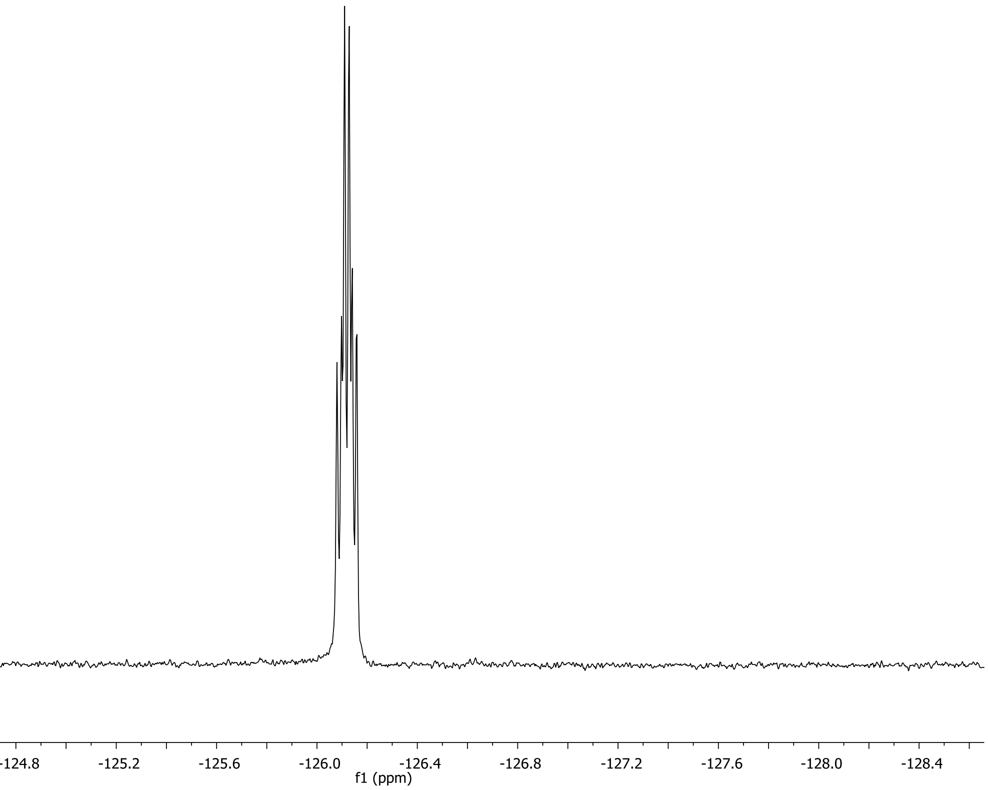

6-Bromo-2,2-dicyclohexylchroman (8h)

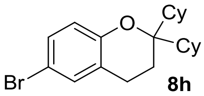

8h

1,1-Dicylohexylprop-2-en-1-ol (**7**, 14.6 mg, 65.7 µmol) and 4-bromophenol (**5h**, 58.4 mg, 338 µmol) were dissolved in toluene (0.14 mL). PPh_3AuNTf_2 (as the 2:1 toluene adduct) (2.6 mg, 3.5 µmol) was added to the resulting solution. The reaction was allowed to stir at 60 °C for 17 hours. The reaction was then filtered over a plug of silica, using diethyl ether as eluent. The crude mixture was purified using column chromatography; using neat hexane. Product **8h** was obtained as a white solid (14.4 mg, 38.2 µmol, 58%).

Mp: 105–107 °C.

ν_{max}/cm^{-1} 2922, 2851, 1476, 1448, 1257, 1243, 1185, 985, 871, 810, 756.

δ_H (300 MHz, $CDCl_3$) 7.18–7.10 (2H, m, Ar-H), 6.69–6.59 (1H, m, Ar-H), 2.64 (2H, t, *J* 6.7 Hz, CH_2), 1.85 (2H, d, *J* 6.7 Hz, CH_2), 1.90–1.61 (12H, m, Cy-H), 1.24–1.01 (10H, m, Cy-H).

δ_C (75 MHz, $CDCl_3$) 153.9 (C), 131.6 (CH), 130.2 (CH), 124.4 (C), 119.0 (CH), 111.0 (C), 82.5 (C), 44.3 (CH), 28.2 (CH), 27.7 (CH_2), 27.5 (CH_2), 27.1 (CH_2), 26.9 (CH_2), 22.8 (CH_2), 22.1 (CH_2).

Found (APCI$^+$) $[M + H]^+$ 377.1473, $C_{21}H_{30}BrO$ requires 377.1475.

elchb077a
1H 300.1MHz Job 26894 Coutant Eloi B077A CDCl3   25.0°C
6-bromo-2,2-dicyclohexylchroman

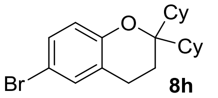

8h

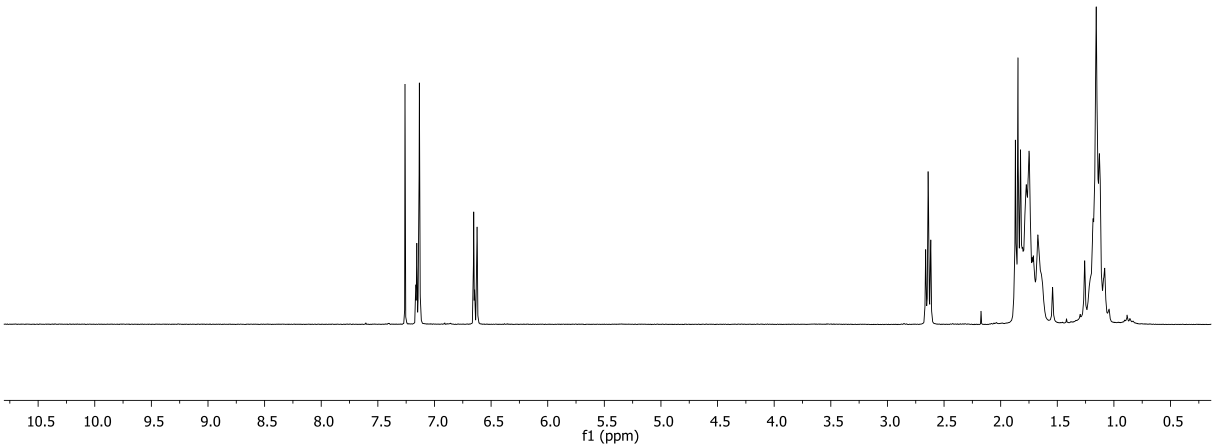

elcc077a
13C 75.5MHz Job 26785 Coutant Eloi 077A CDCl3   25.0°C   3 hours 1 min
6-bromo-2,2-dicyclohexylchroman ?

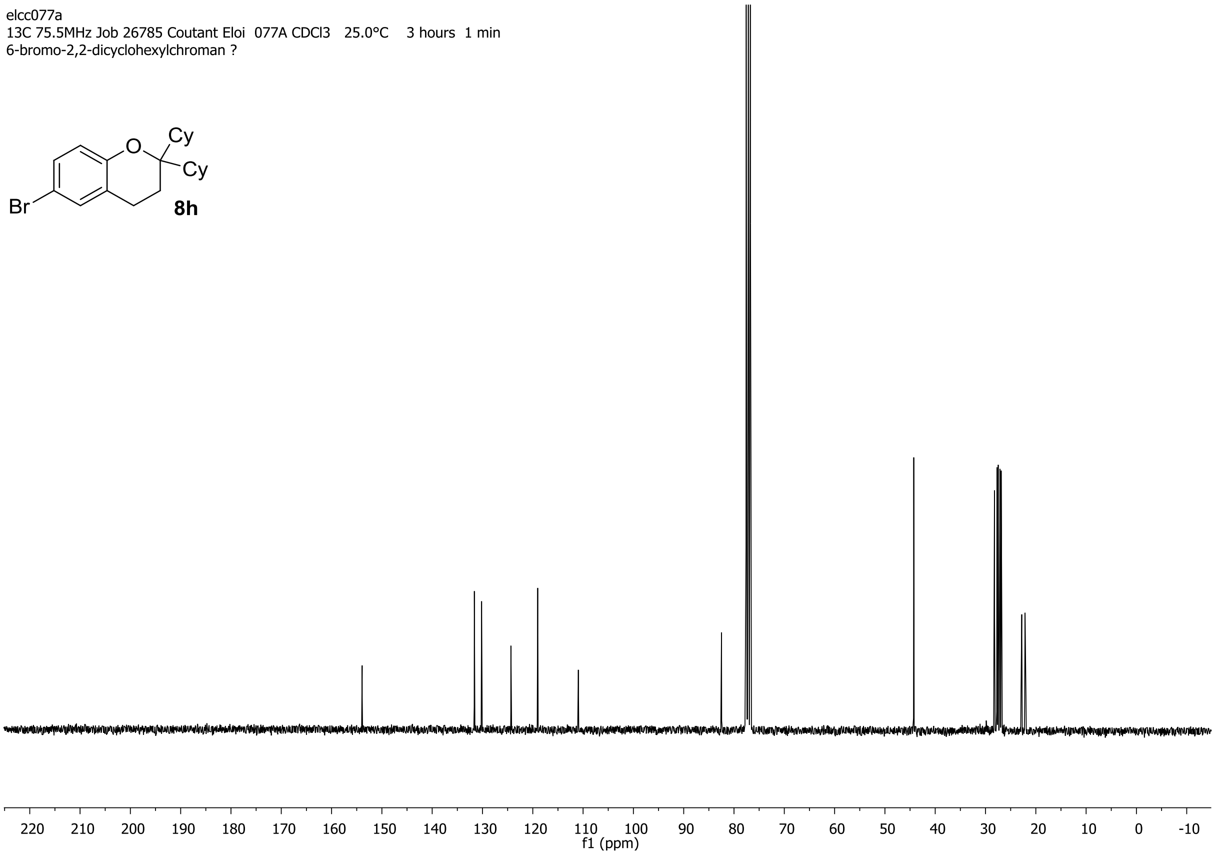

6-(*tert*-Butyl)-2,2-dicyclohexylchroman (8i)

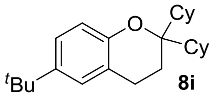

8i

1,1-Dicylohexylprop-2-en-1-ol (**7**, 15.1mg, 67.9 µmol) and 4-(*tert*-butyl)-phenol (**5i**, 20.4 mg, 136 µmol) were dissolved in toluene (0.14 mL). PPh_3AuNTf_2 (as the 2:1 toluene adduct) (2.5 mg, 3.4 µmol) was added to the resulting solution. The reaction was allowed to stir at 60 °C for 17 hours. The reaction was then filtered over a plug of silica, using diethyl ether as eluent. The crude mixture was purified using column chromatography, using a gradient eluent system of neat hexane to 9:1 hexane:diethyl ether. Product **8i** was obtained as as colourless oil (13.8 mg, 38.9 µmol, 57%).

ν_{max}/cm^{-1} 2924, 2852, 1499, 1450, 1363, 1268, 1257, 1244, 1187, 1132, 988, 892, 818, 760.

δ_H (300 MHz, $CDCl_3$) 7.09 (1H, dd, *J* 8.5, 2.5 Hz, Ar-H), 7.00 (1H, d, *J* 2.5 Hz, Ar-H), 6.69 (1H, d, *J* 8.5 Hz, Ar-H), 2.65 (2H, t, *J* 6.6 Hz, CH_2), 1.86 (2H, t, *J* 6.7 Hz, CH_2), 1.94–1.57 (12H, m, Cy-H), 1.29 (9H, s, $C(CH_3)_3$), 1.25–1.07 (10H, m, Cy-H).

δ_C (75 MHz, $CDCl_3$) 152.3 (C), 141.5 (C), 125.8 (CH), 124.3 (CH), 121.0 (C), 116.5 (CH), 81.8 (C), 44.2 (CH_3), 34.1 (C), 31.8 (CH), 28.3 (CH_2), 27.8 (CH_2), 27.5 (CH_2), 27.2 (CH_2), 27.0 (CH_2), 23.1 (CH_2), 22.4 (CH_2).

Found (APCI$^+$) $[M + H]^+$ 355.2994, $C_{25}H_{38}O$ requires 355.2995.

elchb034a
1H 300.1MHz Job 24718 Coutant Eloi B034A CDCl3 25.0°C
50:1 fraction - DiCyAA + 2eq p-tBuPhenol, 60C, o/n

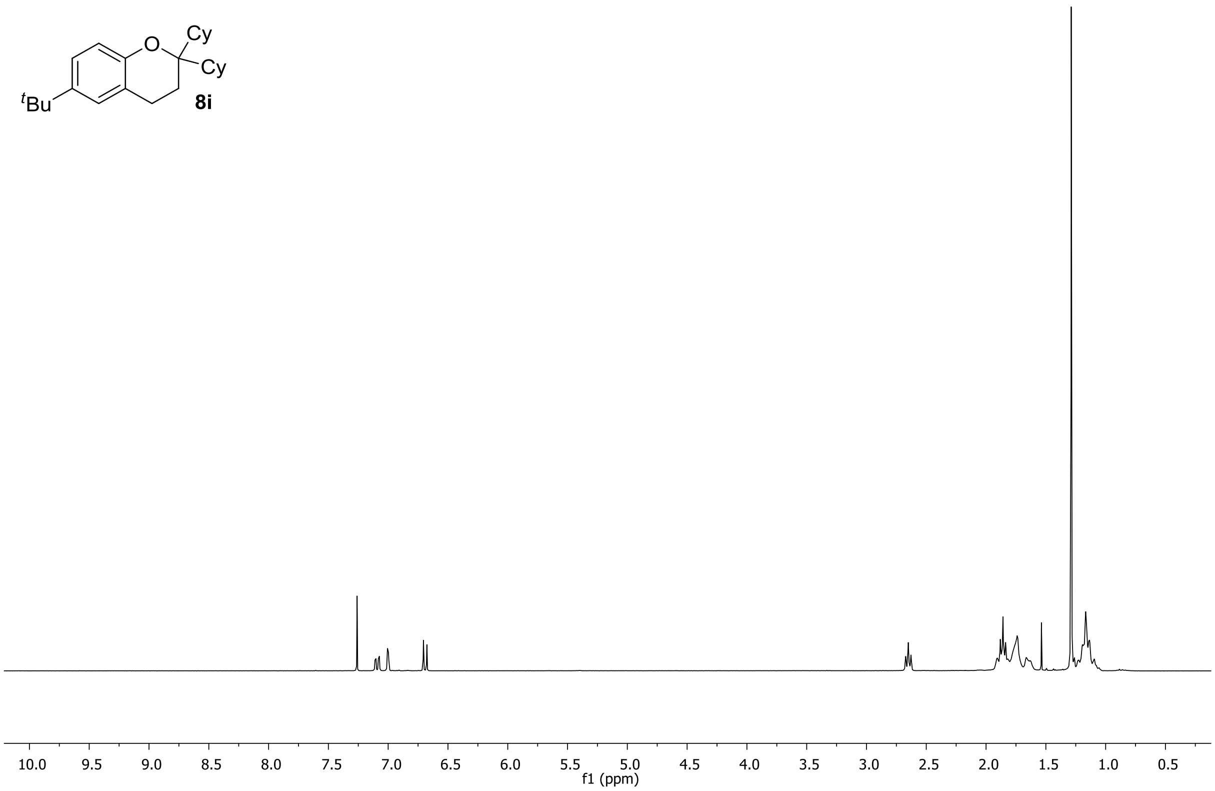

elccb034a
13C 75.5MHz Job 24758 Coutant Eloi B034A CDCl3 25.0°C 3 hours 1 min
p-tBu-DiCyChroman - DiCyAA + 2eq p-tBuPhenol, 60C, o/n

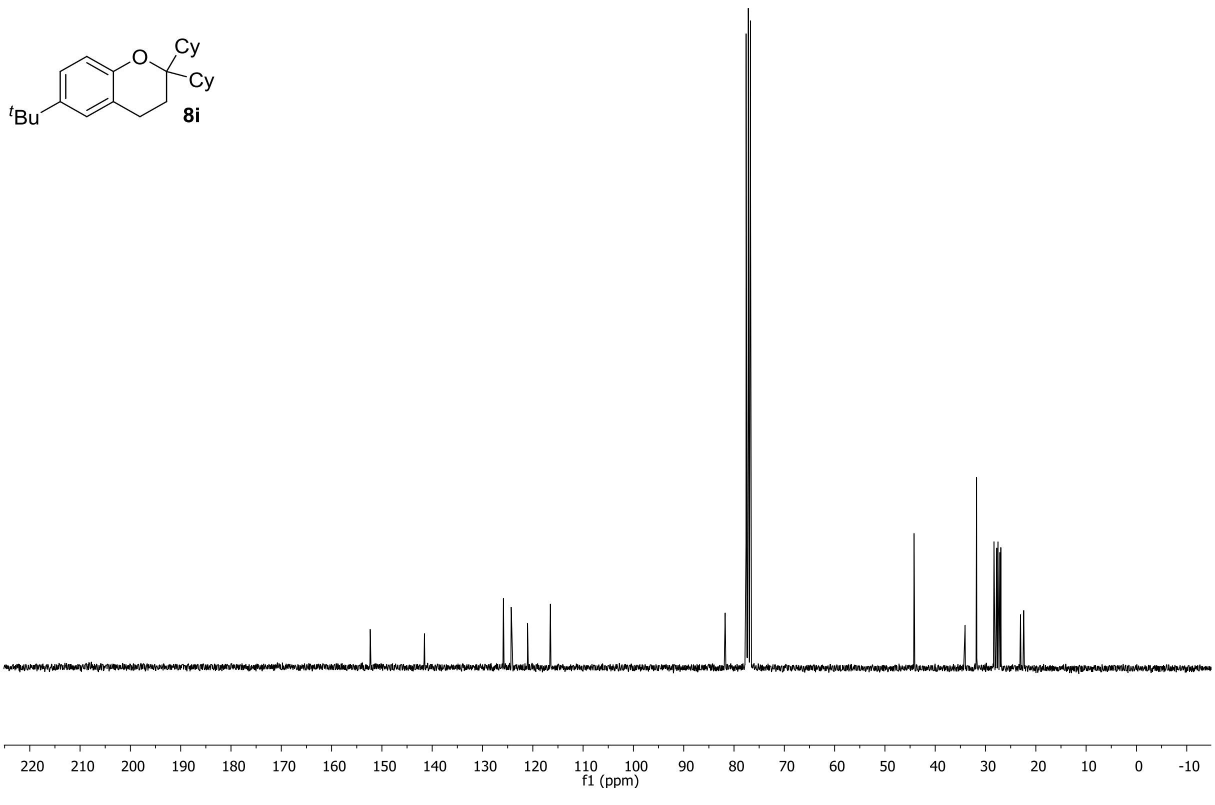

6,8-Di-*tert*-butyl-2,2-dicyclohexylchroman (8j)

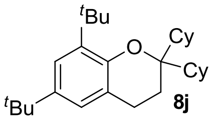

8j

1,1-Dicyclohexylprop-2-en-1-ol (**7**, 19.8 mg, 89.0 µmol) and 2,4-di-*tert*-butylphenol (**5j**, 93.3 mg, 452 µmol) were dissolved in toluene (0.19 mL). PPh_3AuNTf_2 (as the 2:1 toluene adduct) (3.4 mg, 4.6 µmol) was added to the resulting solution. The reaction was allowed to stir at 70 °C for 43 hours. The reaction was then filtered over a plug of silica, using diethyl ether as eluent. The crude mixture was purified using column chromatography, using a gradient eluent system of neat hexane to 9:1 hexane:diethyl ether. Product **8j** was obtained as a colourless oil (30.6 mg, 74.5 µmol, 83%).

ν_{max}/cm^{-1} 2923, 2853, 1470, 1446, 1361, 1231, 1203, 1173, 1128, 939, 895, 874, 754, 733.

δ_H (300 MHz, $CDCl_3$) 7.15 (1H, d, *J* 2.5 Hz, Ar-H), 6.87 (1H, d, *J* 2.5 Hz, Ar-H), 2.69 (2H, t, *J* 6.7 Hz, CH_2), 1.86 (2H, t, *J* 6.7 Hz, CH_2), 2.03–1.58 (12H, m,Cy-H), 1.40 (9H, s, $C(CH_3)_3$), 1.30 (9H, s, $C(CH_3)_3$), 1.29–1.07 (10H, m, Cy-H).

δ_C (75 MHz, $CDCl_3$) 150.7 (C), 140.1 (C), 136.4 (C), 123.9 (CH), 122.0 (CH), 120.7 (C), 83.1 (C), 44.7 (CH), 35.1 (C), 34.1 (C), 31.7 (CH_3), 30.5 (CH_3), 28.5 (CH_2), 28.0 (CH_2), 27.4 (CH_2), 27.0 (CH_2), 26.9 (CH_2), 23.2 (CH_2), 23.1 (CH_2).

Found (NSI^+) $[M + H]^+$ 411.3622, $C_{29}H_{47}O$ requires 411.3621.

elchc059a
1H 300.1MHz Job 26081 Coutant Eloi C059A CDCl3 25.2°C
A fraction - DiCyAA + 5eq 2,4-di(tBu)phenol, 70C, 2d

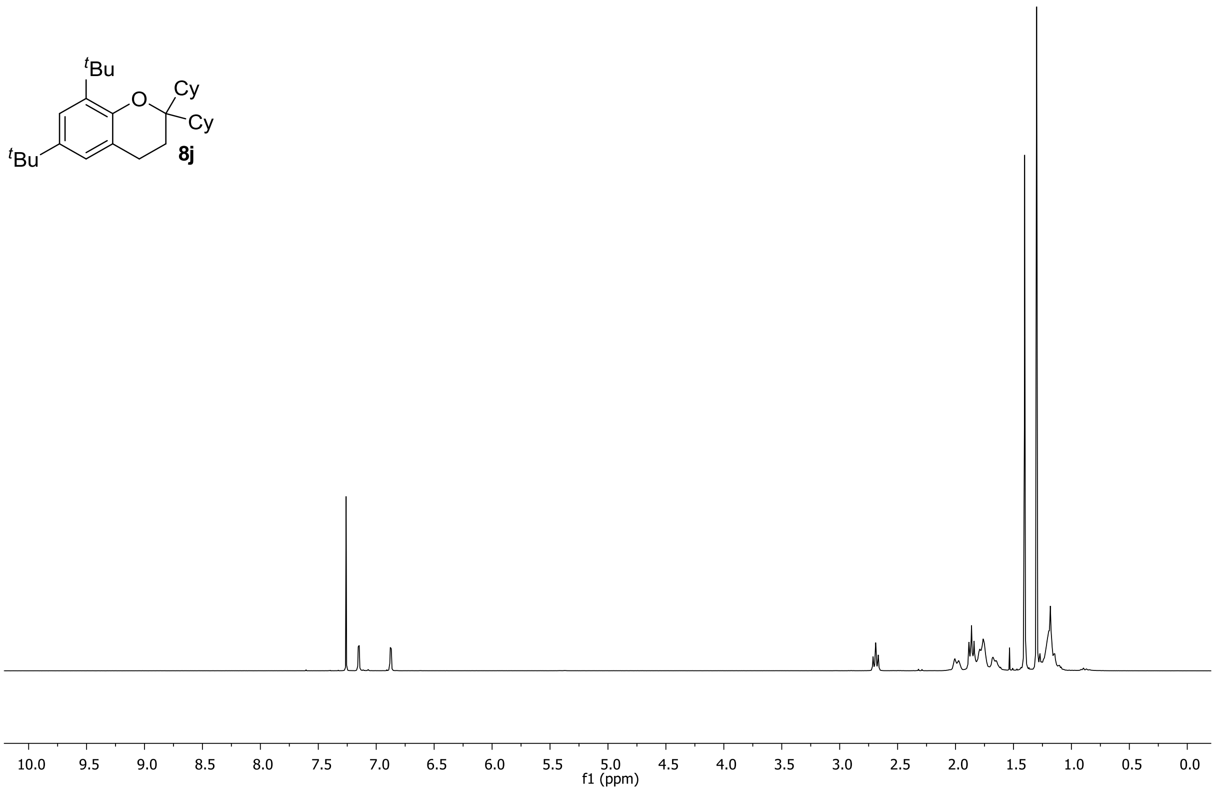

elccb059a
13C 75.5MHz Job 26130 Coutant Eloi B059A CDCl3 25.0°C 0 hour 36 min
A fraction - DiCyAA + 5eq 2,4-di(tBu)phenol, 70C, 2d

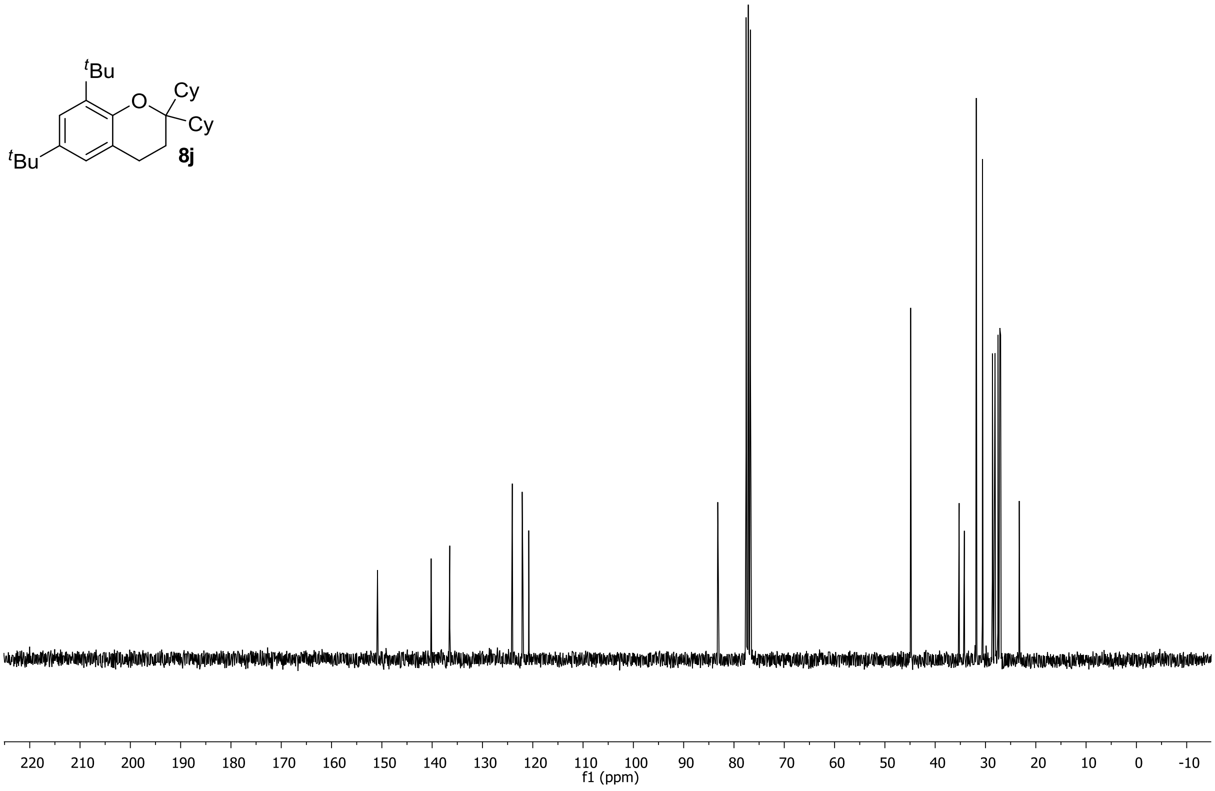

Methyl 2,2-dicyclohexylchroman-6-carboxylate (**8k**)

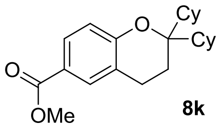

8k

1,1-Dicylohexylprop-2-en-1-ol (**7**, 14.7 mg, 66.1 µmol) and methyl 4-hydroxybenzoate (**5k**, 51.5 mg, 338 µmol) were dissolved in dioxane (0.21 mL). PPh_3AuNTf_2 (as the 2:1 toluene adduct) (2.5 mg, 3.4 µmol) was added to the resulting solution. The reaction was allowed to stir at 70 °C for 48 hours and 80 °C for 17 hours. The reaction was then filtered over a plug of silica, using ethyl acetate as eluent. The crude mixture was purified using column chromatography (10:1 hexane:diethyl ether). Product **8k** was obtained yellowish oil (16.3 mg, 45.7 µmol, 69%).

ν_{max}/cm^{-1} 2922, 2852, 1717, 1611, 1582, 1495, 1438, 1285, 1259, 1193, 1178, 1115, 1101, 983, 769, 755.

δ_H (300 MHz, $CDCl_3$) 7.77 (2H, m, Ar-H), 6.80–6.75 (1H, m, Ar-H), 3.86 (3H, s, CH_3), 2.70 (2H, t, *J* 6.7 Hz, CH_2), 1.89 (2H, t, *J* 6.7 Hz, CH_2), 1.94–0.70 (22H, m, Cy-H).

δ_C (75 MHz, $CDCl_3$) 167.4 (C), 159.1 (C), 131.4 (CH), 129.3 (CH), 121.8 (C), 121.0 (C), 117.1 (CH), 83.4 (C), 51.8 (CH_3), 44.4 (CH), 28.2 (CH_2), 27.7 (CH_2), 27.5 (CH_2), 27.1 (CH_2), 26.9 (CH_2), 22.9 (CH_2), 22.0 (CH_2).

Found (NSI^+) $[M + H]^+$ 357.2425, $C_{23}H_{33}O_3$ requires 357.2424.

elcha083a
1H 300.1MHz Job 27282 Coutant Eloi A083A CDCl3 25.0°C
Isolated product - EC83:A

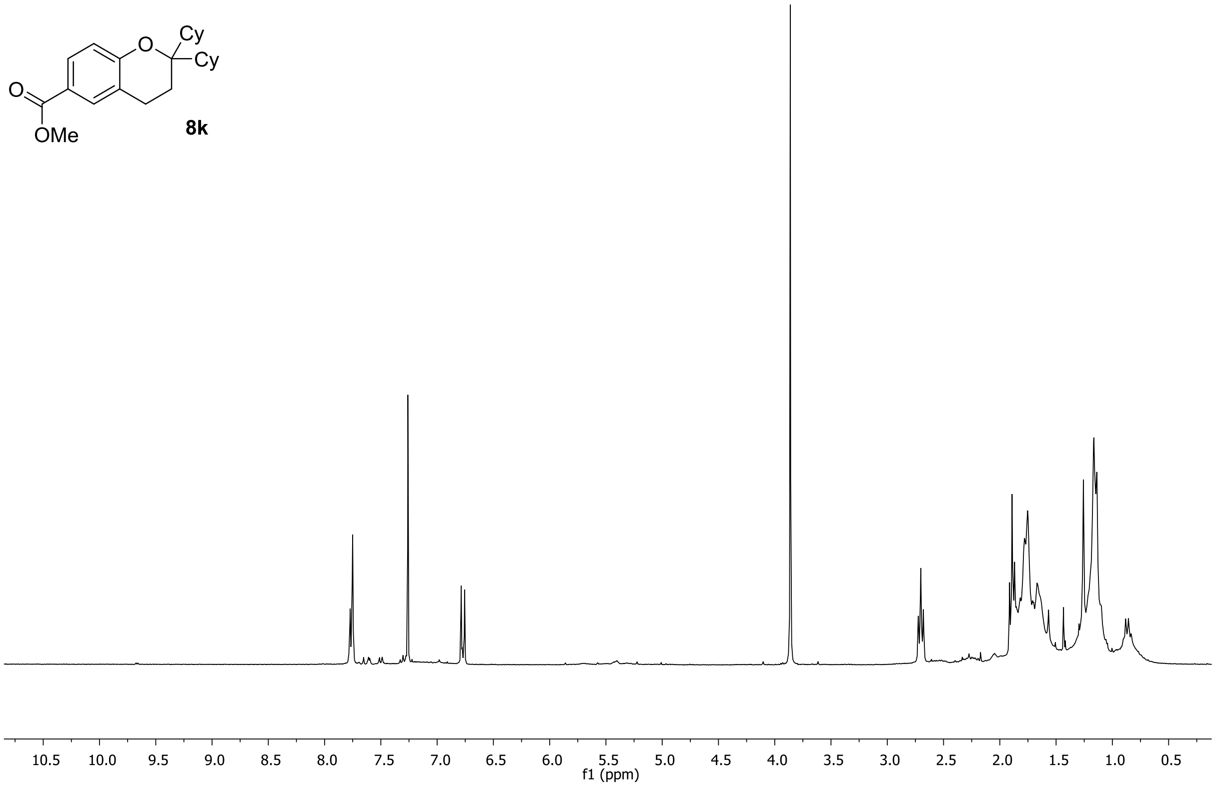

elcca083a
13C 75.5MHz Job 27339 Coutant Eloi A083A CDCl3 25.0°C 3 hours 1 min
EC83:A

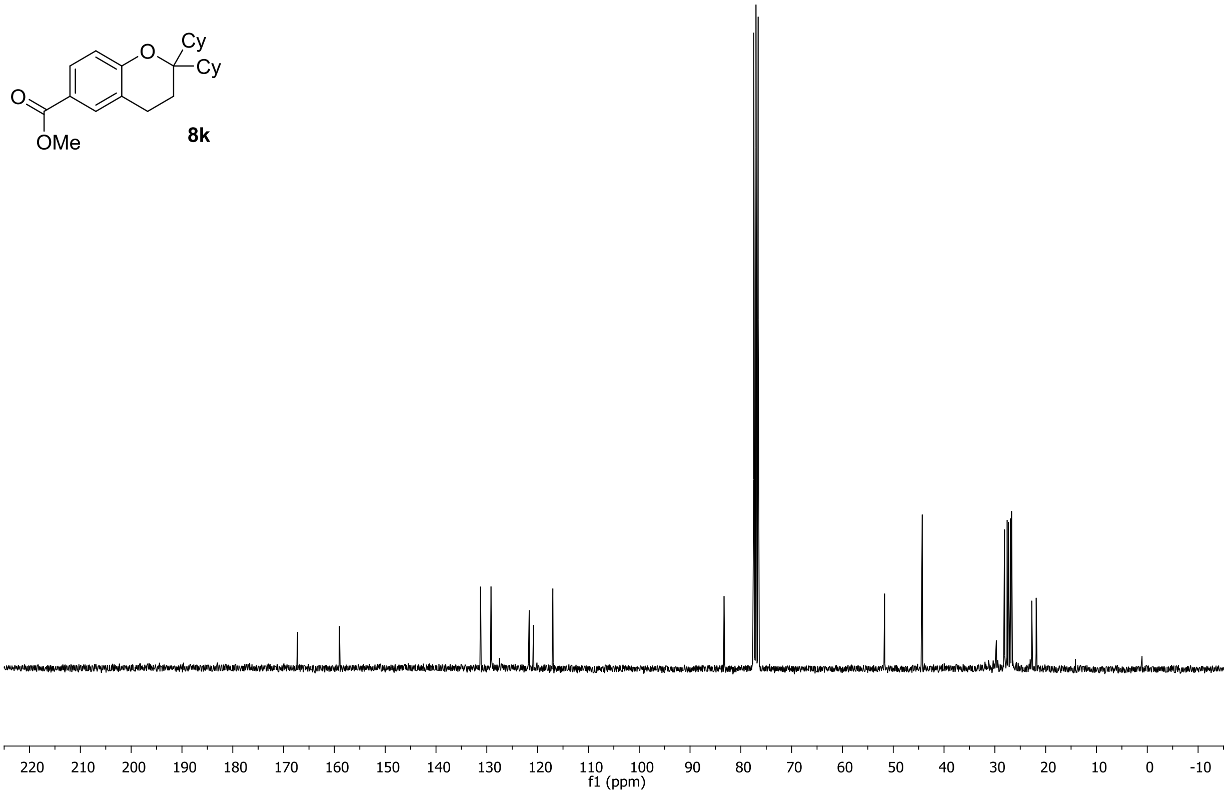

2,2-Dicyclohexyl-5,7,8-trimethylchroman-6-ol (8l)

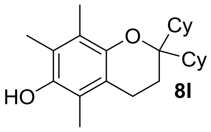

8l

Reaction carried out in a sealed tube. 1,1-Dicylohexylprop-2-en-1-ol (**7**, 15.4 mg, 69.2 µmol) and trimethylhydroquinone (**5l**, 51.2 mg, 336 µmol) were dissolved in dioxane (0.14 mL). PPh_3AuNTf_2 (as the 2:1 toluene adduct) (2.7 mg, 3.7 µmol) was added to the resulting solution. The reaction was tightly sealed and allowed to stir at 90 °C for 65.5 hours. The reaction was then filtered over a plug of silica, using ethyl acetate as eluent. The crude mixture was purified using column chromatography, using a gradient eluent system of neat hexane to 20:1 hexane:ethyl acetate. Product **8l** was obtained as a brown oil (20.5 mg, 57.5 µmol, 83%).

ν_{max}/cm^{-1} 3465, 2923, 2852, 1449, 1420, 1260, 1213, 1082, 1067, 908, 733.

δ_H (300 MHz, $CDCl_3$) 4.15 (1H, s, O<u>H</u>), 2.54 (2H, t, *J* 6.8 Hz, C<u>H$_2$</u>), 2.16 (3H, s, C<u>H$_3$</u>), 2.11 (6H, s, C<u>H$_3$</u>), 1.85 (2H, t, *J* 6.8 Hz, C<u>H$_2$</u>), 1.90–1.53 (12H, m, Cy-<u>H</u>), 1.23–1.06 (10H, m, Cy-<u>H</u>).

δ_C (75 MHz, $CDCl_3$) 145.9 (C), 144.3 (C), 122.6 (C), 121.1 (C), 118.3 (C), 117.9 (C), 80.1 (C), 43.8 (CH), 28.4 (CH_2), 28.0 (CH_2), 27.6 (CH_2), 27.2 (CH_2), 27.0 (CH_2), 23.4 (CH_2), 20.5 (CH_2), 12.4 (CH_3), 12.0 (CH_3), 11.4 (CH_3).

Found (NSI^+) $[M + H]^+$ 357.2788, $C_{24}H_{37}O_2$ requires 357.2788.

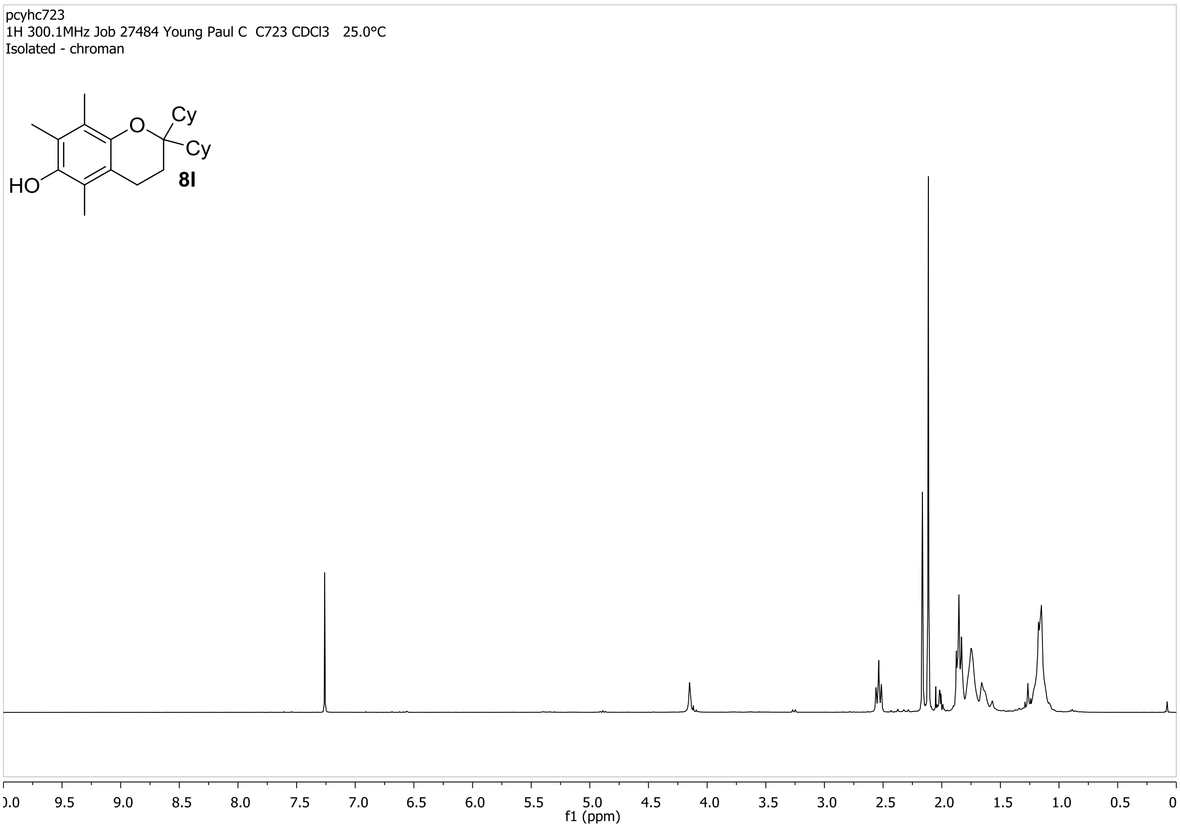
pcyhc723
1H 300.1MHz Job 27484 Young Paul C C723 CDCl3 25.0°C
Isolated - chroman
Cy
Cy
HO
O
8l
9.5
9.0
8.5
8.0
7.5
7.0
6.5
6.0
5.5
5.0
4.5
4.0
3.5
3.0
2.5
2.0
1.5
1.0
0.5
0
f1 (ppm)

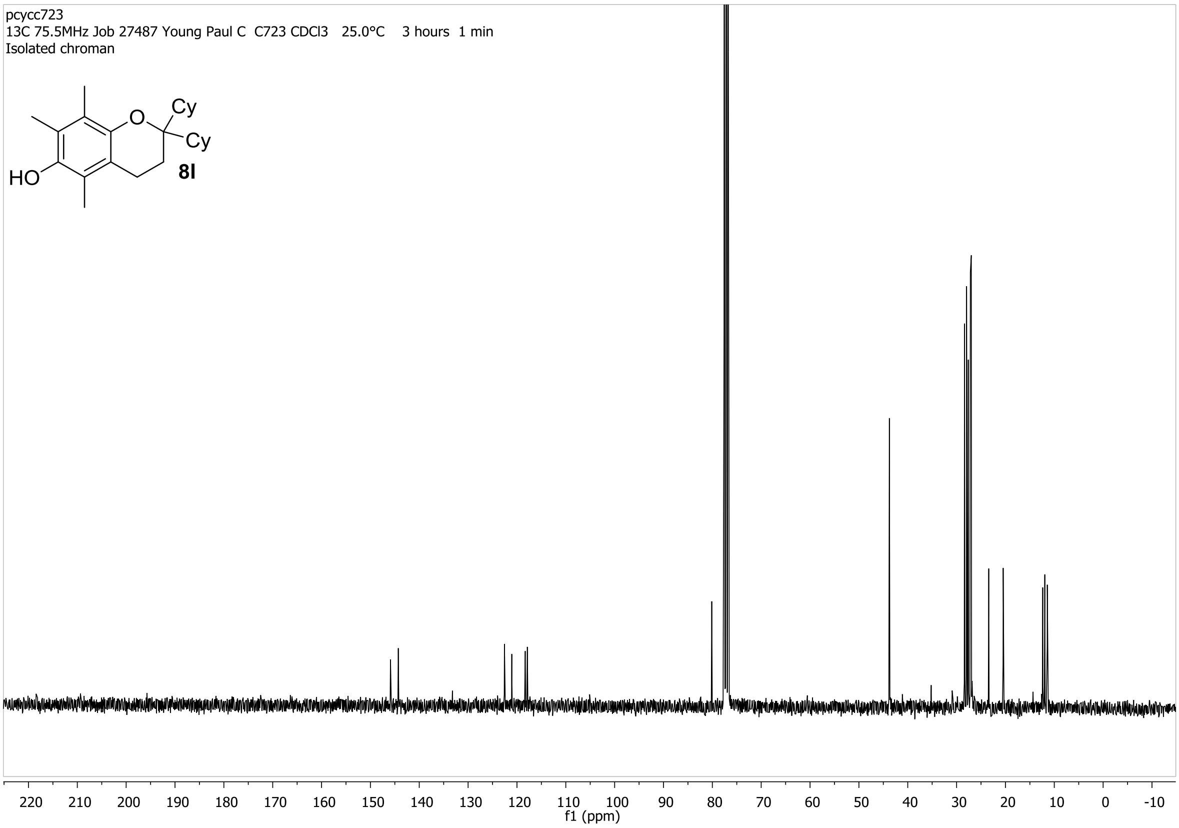
pcycc723
13C 75.5MHz Job 27487 Young Paul C C723 CDCl3 25.0°C 3 hours 1 min
Isolated chroman
Cy
Cy
HO
O
8l
220
210
200
190
180
170
160
150
140
130
120
110
100
90
80
70
60
50
40
30
20
10
0
-10
f1 (ppm)

2-(3,3-Dicyclohexylallyl)-3,5,6-trimethylcyclohexa-2,5-diene-1,4-dione (11)

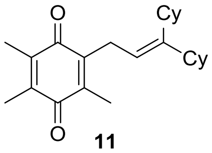

11

1,1-Dicylohexylprop-2-en-1-ol (**7**, 15.1 mg, 67.9 µmol) and trimethylhydroquinone (**5l**, 51.0 mg, 335 µmol) were dissolved in dioxane (0.14 mL). PPh_3AuNTf_2 (as the 2:1 toluene adduct) (2.5 mg, 3.4 µmol) was added to the resulting solution. The reaction was sealed and allowed to stir at 80 °C for 65.5 hours. The reaction was then filtered over a plug of silica, using ethyl acetate as eluent. The crude mixture was purified using column chromatography, using a gradient eluent system of neat hexane to 7:1 hexane:ethyl acetate. Product **11** was obtained as a yellow oil (8.5 mg, 24.0 µmol, 35%). (The major product is chroman **8l** at 45%)

ν_{max}/cm^{-1} 2924, 2851, 1642, 1448, 1374, 1302, 1262.

δ_H (300 MHz, $CDCl_3$) 4.88 (1H, t, *J* 6.9 Hz, C<u>H</u>), 3.25 (2H, d, *J* 6.9 Hz, C_{H2}), 2.63–2.48 (1H, m, Cy-<u>H</u>), 2.05–1.96 (9H, m, 3xC<u>H</u>$_3$), 1.92–0.97 (21H, m, Cy-<u>H</u>).

δ_C (75 MHz, $CDCl_3$) 188.2 (C), 187.1 (C), 152.0 (C), 144.0 (C), 140.6 (C), 140.4 (C), 122.1 (C), 117.5 (CH), 41.1 (CH), 40.1 (CH), 35.2 (CH_2), 30.9 (CH_2), 27.3 (CH_2), 26.8 (CH_2), 26.4 (CH_2), 26.3 (CH_2), 24.9 (CH_2), 12.6 (CH_3), 12.5 (CH_3), 12.3 (CH_3).

Found (NSI$^+$) $[M + H]^+$ 355.2630, $C_{24}H_{35}O_2$ requires 355.2632.

pcyhb722.1.fid
1H 300.1MHz Job 27363 Young Paul C B722 CDCl3 25.1°C
Isolated top spot

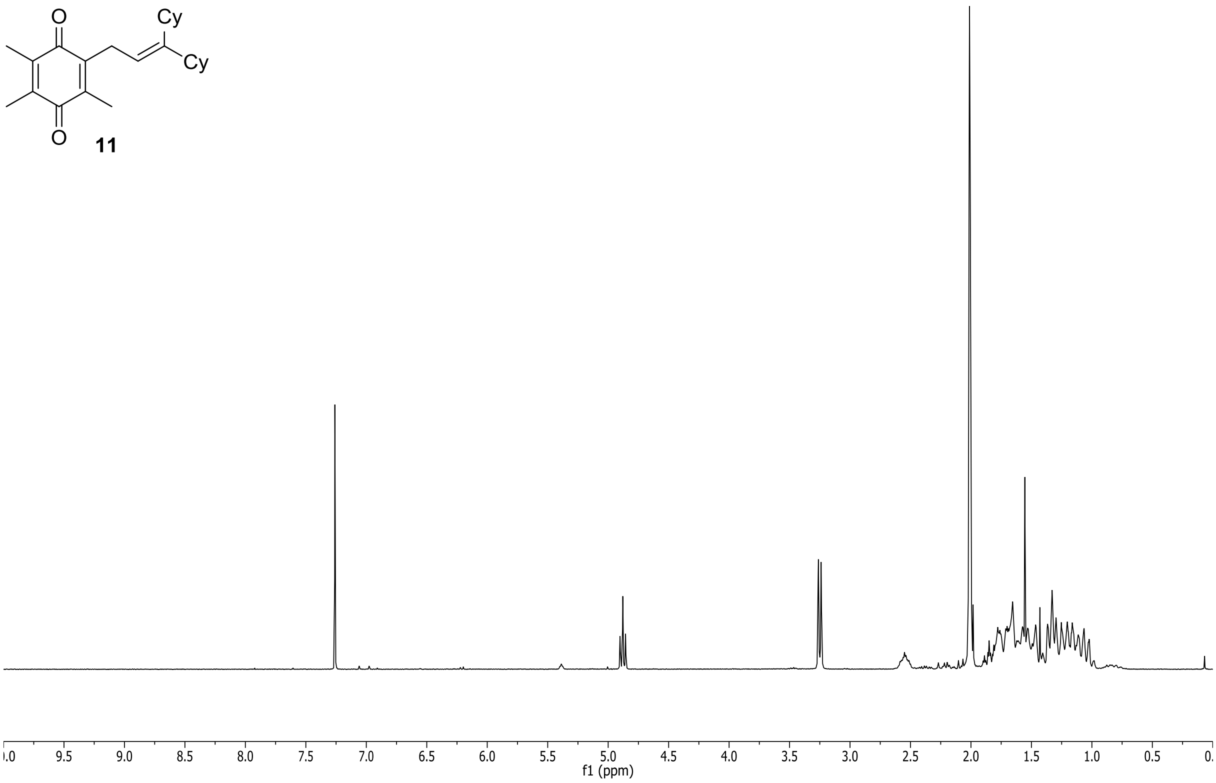

pcycb722.1.fid
13C 75.5MHz Job 27407 Young Paul C B722 CDCl3 24.9°C 3 hours 1 min
Isolated top spot

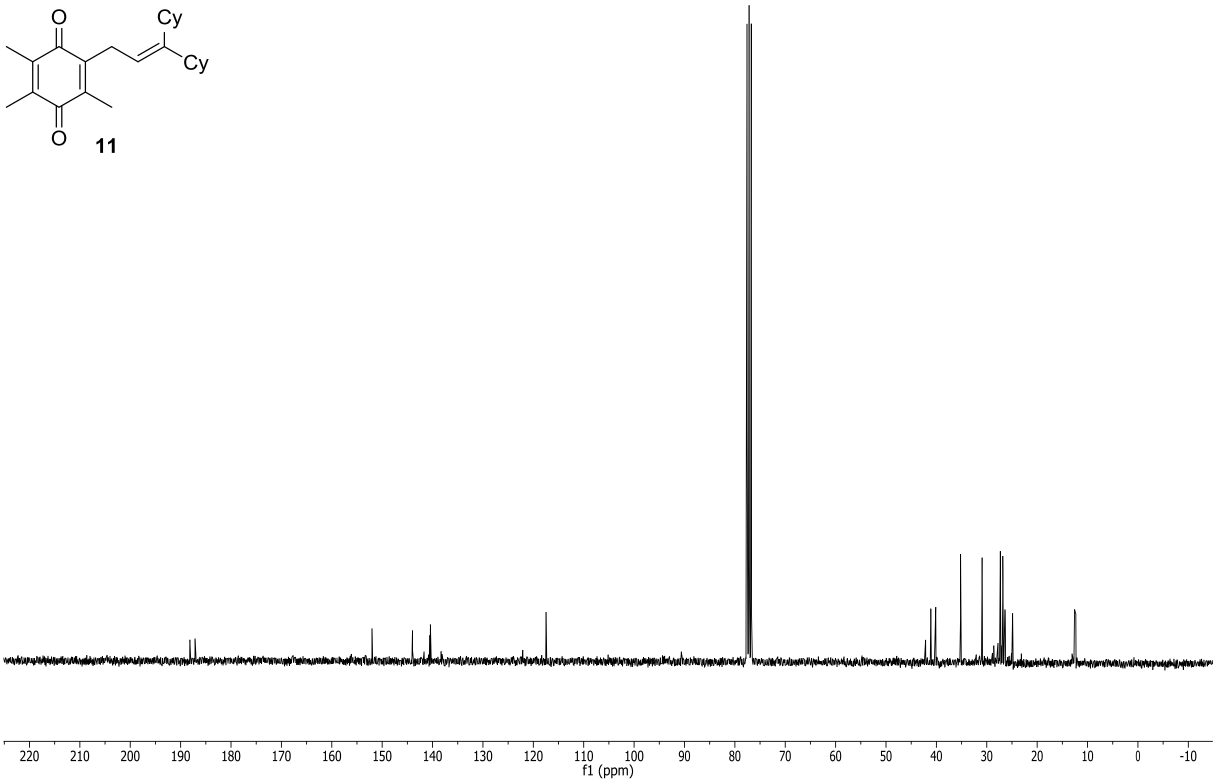

6,8-Di-*tert*-butyl-2,2-dihexylchroman **(8m)**

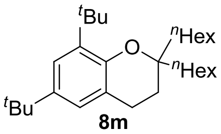

8m

7-Vinyltridecan-7-ol (**12**, 19.9 mg, 87.9 µmol) and 2,4-di-*tert*-butylphenol (**5j**, 91.1 mg, 442 µmol) were dissolved in toluene (0.20 mL). PPh_3AuNTf_2 (as the 2:1 toluene adduct) (3.4 mg, 4.6 µmol) was added to the resulting solution. The reaction was allowed to stir at 60 °C for 43 hours. The reaction was then filtered over a plug of silica, using diethyl ether as eluent. The crude product was purified using column chromatography, using a gradient eluent system of neat pentane to 9:1 pentane:diethyl ether. Product **8m** was obtained as a colourless oil (25.0 mg, 60.3 µmol, 69%).

ν_{max}/cm^{-1} 2953, 2928, 2859, 1467, 1444, 1361, 1231, 1203, 1127, 1056, 945, 876, 756.

δ_H (300 MHz, $CDCl_3$) 7.15 (1H, d, *J* 2.5 Hz, Ar-H), 6.90 (1H, d, *J* 2.5 Hz, Ar-H), 2.72 (2H, t, *J* 6.7 Hz, $C\underline{H_2}CH_2C$), 1.78 (2H, t, *J* 6.7 Hz, $CCH_2C\underline{H_2}C$), 1.67–1.57 (4H, m, 2x$C\underline{H_2}(CH_2)_4CH_3$), 1.39 (9H, s, $C(C\underline{H_3})_3$), 1.30 (9H, s, $C(C\underline{H_3})_3$), 1.36-1.26 (16H, m, 2x$CH_2(C\underline{H_2})_4CH_3$), 0.89 (6H, t, *J* 6.6 Hz, 2x$(CH_2)_5C\underline{H_3}$).

δ_C (75 MHz, $CDCl_3$) 150.2 (C), 140.6 (C), 136.8 (C), 124.3 (CH), 121.9 (CH), 120.2 (C), 78.6 (C), 36.9 (CH_2), 35.2 (C), 34.2 (C), 32.0 (CH_2), 31.8 (CH_3), 30.08 (CH_2), 30.06 (CH_3), 28.5 (CH_2), 23.7 (CH_2), 23.0 (CH_2), 22.8 (CH_2), 14.2 (CH_3).

Found (APCI$^+$) $[M + H]^+$ 415.3925, $C_{29}H_{51}O$ requires 415.3934.

elchb058a
1H 300.1MHz Job 26033 Coutant Eloi B058A CDCl3 25.1°C
A fraction - Hexyl/Hexyl AA + 2,4-di(tBu)phenol, 60C, 2d

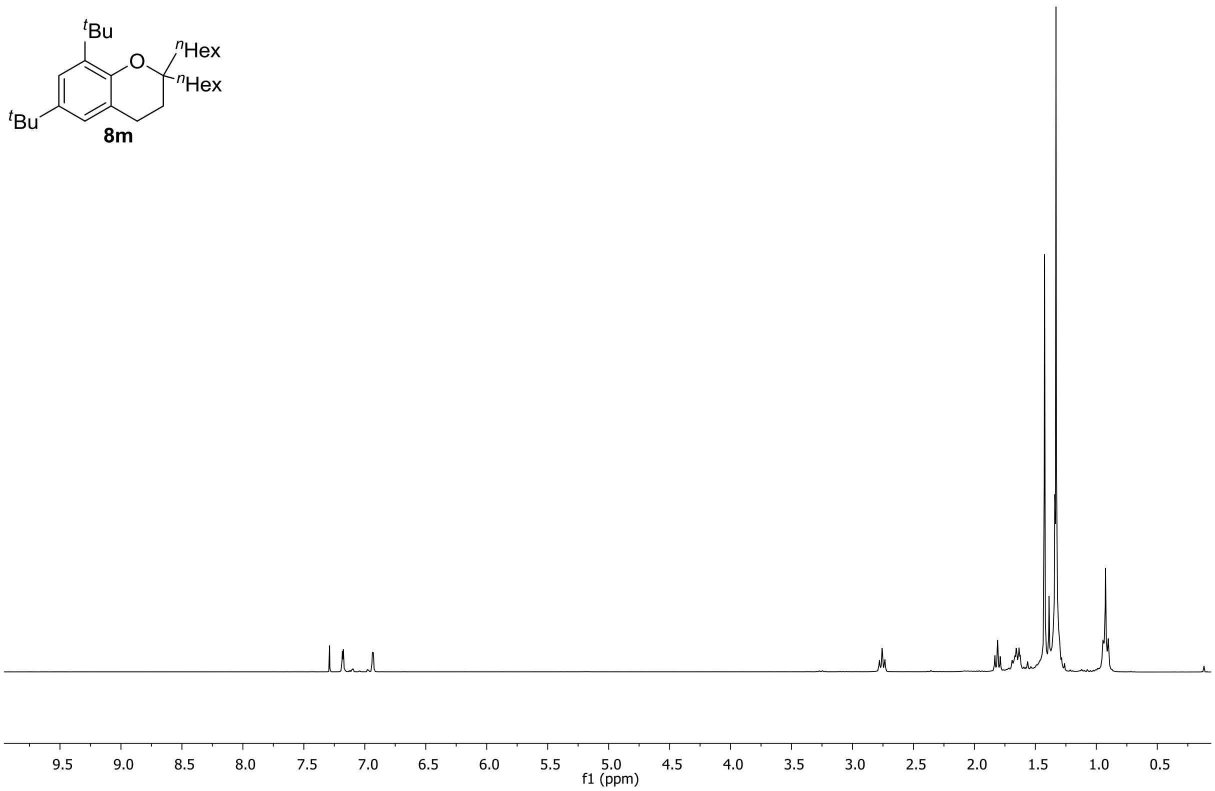

elccb058a
13C 75.5MHz Job 26096 Coutant Eloi B058A CDCl3 25.0°C 0 hour 36 min
A fraction - Hexyl/Hexyl AA + 2,4-di(tBu)phenol, 60C, 2d

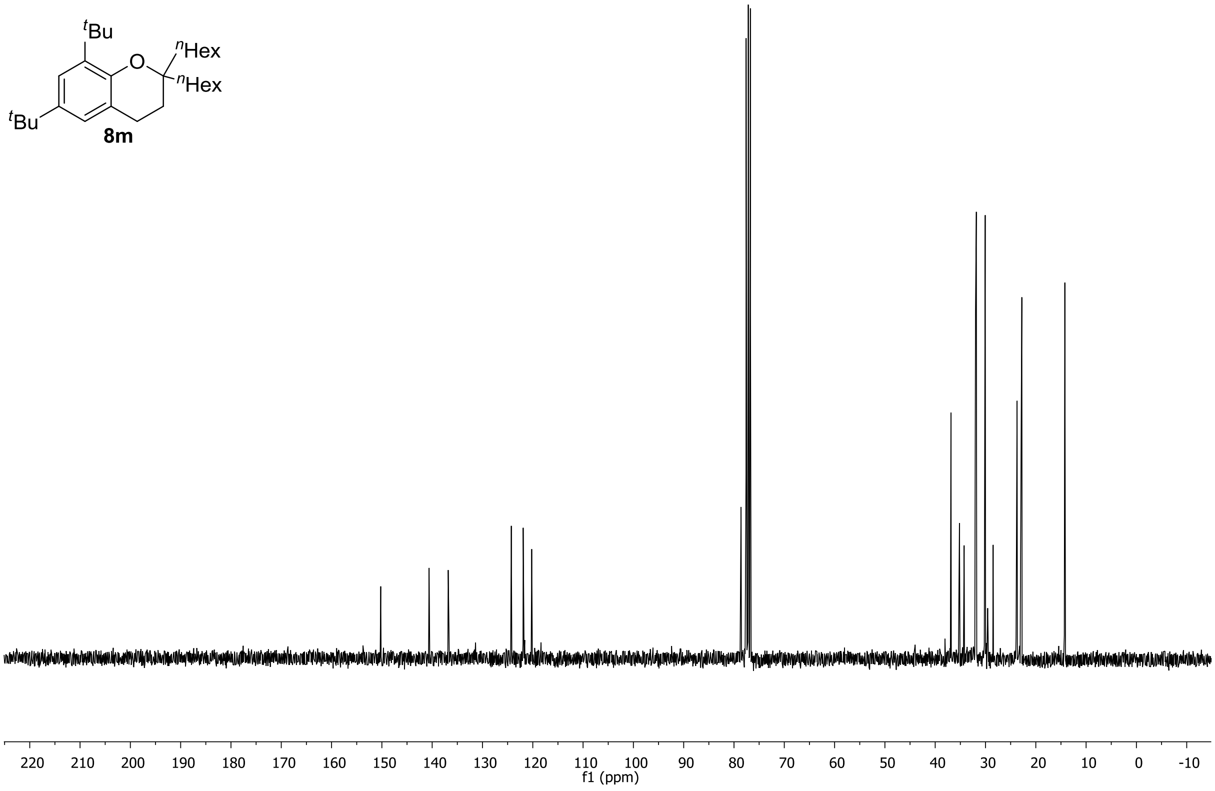

2,6,8-Tri-*tert*-butyl-2-methylchroman **(8n)**

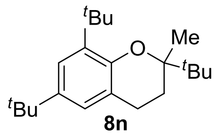

8n

3,4,4-Trimethylpent-1-en-3-ol (**4**, 20.5 mg, 159.9 µmol) and 2,4-di-*tert*-butylphenol (**5j**, 162.9 mg, 790 µmol) were dissolved in toluene (0.34 mL). PPh_3AuNTf_2 (as the 2:1 toluene adduct) (6 mg, 8.1 µmol) was added to the resulting solution. The reaction was allowed to stir at 60 °C for 67 hours. The reaction was then filtered over a plug of silica, using diethyl ether as the eluent. The crude mixture was purified using column chromatography, using a gradient eluent system of neat hexane to neat diethyl ether. Product **8n** was obtained as a yellowish oil (41.9 mg, 132 µmol, 83%).

ν_{max}/cm^{-1} 2955, 2871, 1473, 1444, 1373, 1364, 1237, 1223, 1153, 1130, 1093, 963, 906, 875, 755.

δ_H (300 MHz, $CDCl_3$) 7.18 (1H, d, *J* 2.5 Hz, Ar-$\underline{H}$), 6.95 (1H, d, *J* 2.5 Hz, Ar-$\underline{H}$), 2.90 (1H, ddd, *J* 16.7, 13.0, 6.3 Hz, C$\underline{H}$HCH$_2$), 2.76 (1H, ddd, *J* 16.7, 6.2, 1.8 Hz, CH$\underline{H}$CH$_2$), 1.99 (1H, td, *J* 13.0, 6.2 Hz, CH$_2$C$\underline{H}$H), 1.75 (1H, ddd, *J* 13.0, 6.3 Hz, 1.8, CH$_2$CH$\underline{H}$), 1.43 (9H, s, C(C$\underline{H_3}$)$_3$), 1.32 (9H, s, C(C$\underline{H_3}$)$_3$), 1.21 (3H, s, C$\underline{H_3}$), 1.13 (9H, s, C(C$\underline{H_3}$)$_3$).

δ_C (75 MHz, $CDCl_3$) 150.6 (C), 140.9 (C), 137.2 (C), 124.3 (CH), 121.9 (CH), 120.2 (CH), 80.7 (C), 38.1 (C), 35.2 (C), 34.3 (C), 31.8 (CH_3), 30.0 (CH_3), 25.8 (CH_3), 25.6 (CH_2), 23.4 (CH_2), 17.0 (CH_3).

Found (APCI$^+$) [M]$^+$ 316.2754, $C_{22}H_{36}O$ requires 316.2761.

elchb056a
1H 300.1MHz Job 25906 Coutant Eloi B056A CDCl3 24.9°C
A fraction - Me/tBu AA + 5eq 2,4-di(tBu)phenol, 60C, over w-e

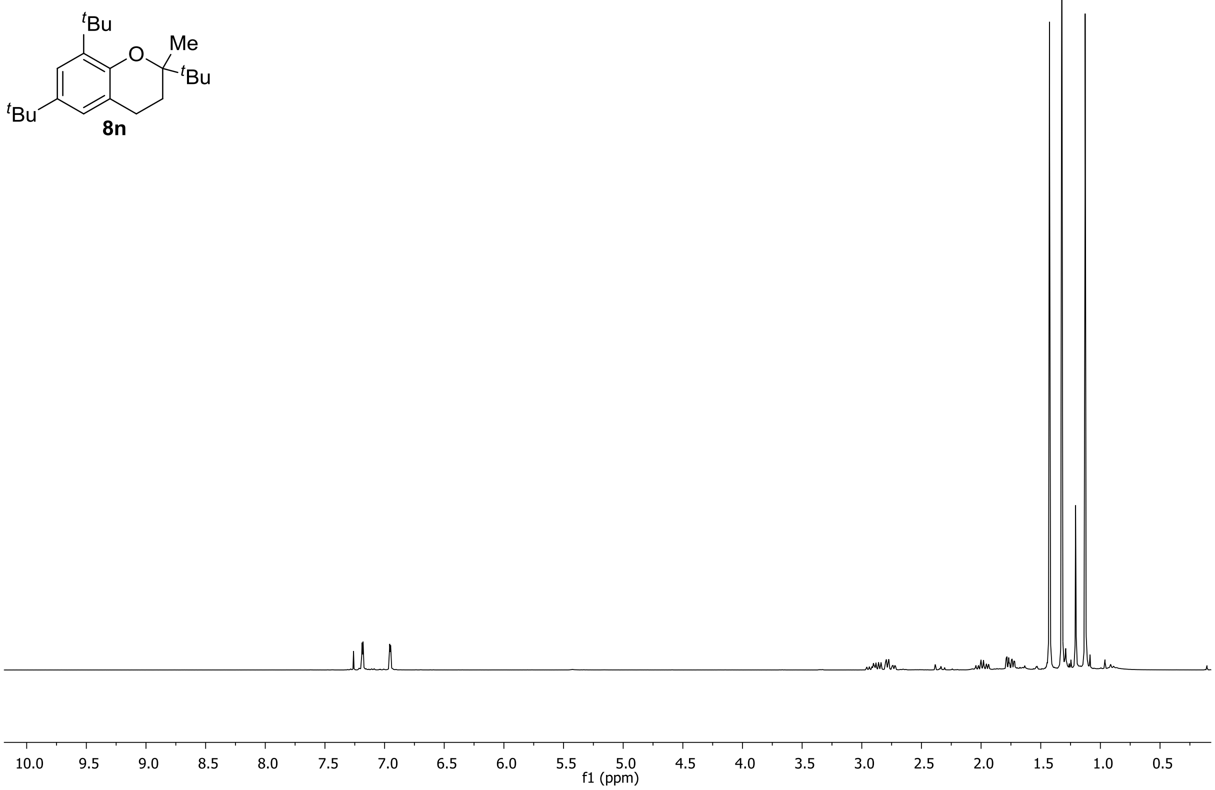

elccb056a
13C 75.5MHz Job 25918 Coutant Eloi B056A CDCl3 25.0°C 0 hour 18 min
A fraction - Me/tBu AA + 5eq 2,4-di(tBu)phenol, 60C, over w-e

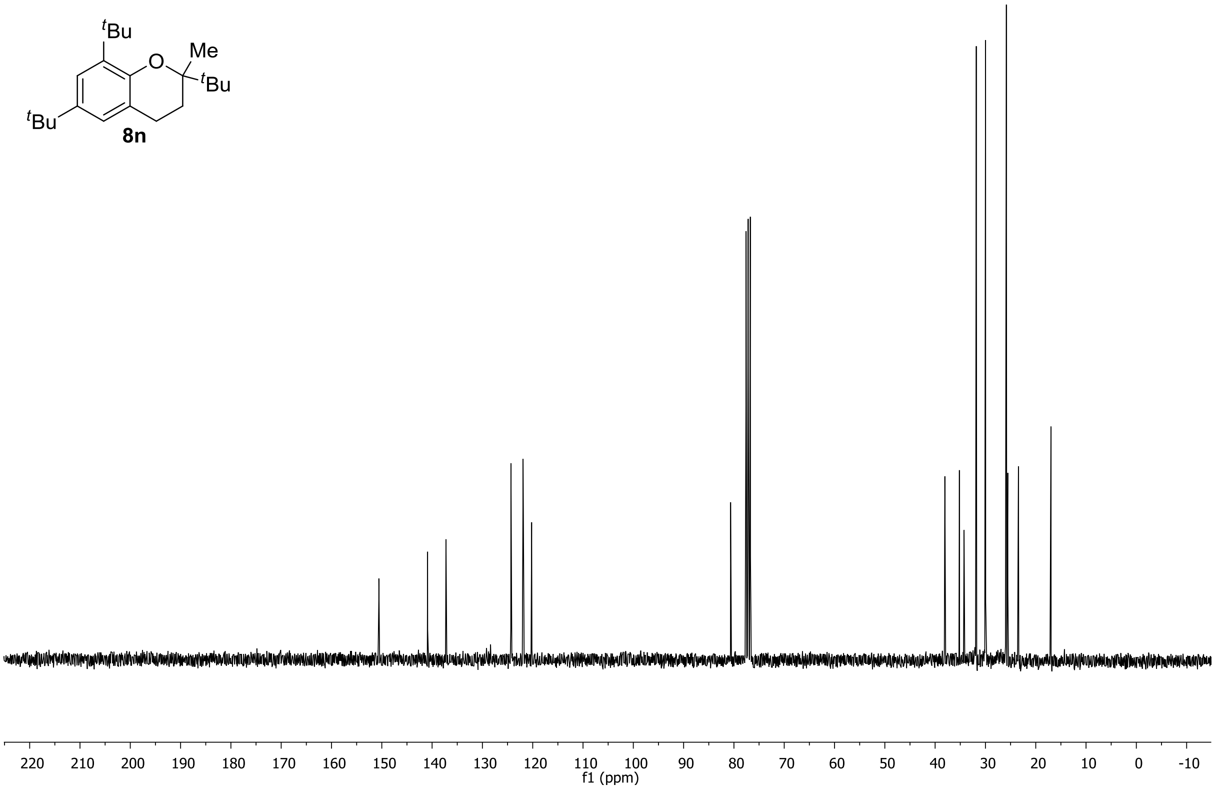

6,8-Di-*tert*-butyl-2,2-dimethylchroman (**8o**)

With a tertiary allylic alcohol:

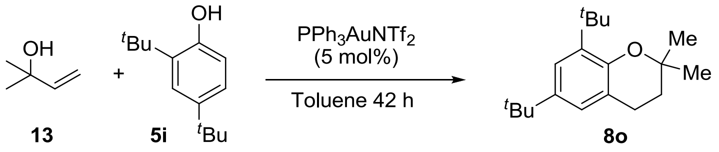

2-Methylbut-3-en-2-ol (**13**, 15.1 mg, 175 µmol) and 2,4-di-*tert*-butylphenol (**5j**, 182 mg, 880 µmol) were dissolved in toluene (0.36 mL). PPh_3AuNTf_2 (as the 2:1 toluene adduct) (6.4 mg, 8.7 µmol) was added to the resulting solution. The reaction was allowed to stir at 60 °C for 42 hours. The reaction was then filtered over a plug of silica, using diethyl ether as eluent. The crude mixture was purified using column chromatography, using neat hexane as eluent. Product **8o** was obtained as a colourless oil (30.6 mg, 112 µmol, 64%).

With a primary allylic alcohol:

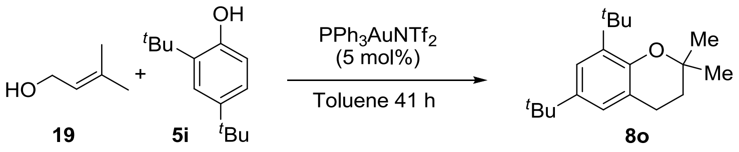

3-Methylbut-2-enol (**19**, 15.2 mg, 177 µmol) and 2,4-di-*tert*-butylphenol (**5j**, 181 mg, 876 µmol) were dissolved in toluene (0.36 mL). PPh_3AuNTf_2 (as the 2:1 toluene adduct) (6.4 mg, 8.7 µmol) was added to the resulting solution. The reaction was allowed to stir at 60 °C for 41 hours. The reaction was then filtered over a plug of silica, using diethyl ether as eluent. The crude product was purified using column chromatography, using neat hexane as eluent. Product **8o** was obtained as a colourless oil (37.6 mg, 137.0 µmol, 78%).

ν_{max}/cm^{-1} 2952, 2868, 1478, 1444, 1415, 1389, 1382, 1361, 1251, 1238, 1221, 1203, 1158, 1127, 940, 894, 876, 759, 752.

δ_H (300 MHz, $CDCl_3$) 7.17 (1H, d, *J* 2.5 Hz, Ar-H), 6.94 (1H, d, *J* 2.5 Hz, Ar-H), 2.81 (2H, t, *J* 6.8 Hz, CH_2), 1.80 (2H, t, *J* 6.8 Hz, CH_2), 1.41 (9H, s, $C(CH_3)_3$), 1.38 (6H, s, $C(CH_3)_2$), 1.32 (9H, s, $C(CH_3)_3$).

δ_C (75 MHz, $CDCl_3$) 150.3 (C), 140.9 (C), 136.9 (C), 124.2 (C), 121.8 (CH), 119.9 (C), 73.8 (C), 35.1 (C), 34.3 (C), 33.0 (CH_2), 31.8 (CH_3), 30.0 (CH_3), 27.3 (CH_3), 23.5 (CH_2).

Found (APCI+) $[M]^+$ 274.2287, $C_{19}H_{30}O$ requires 274.2291.

elchb069a
1H 300.1MHz Job 26551 Coutant Eloi B069A CDCl3 25.0°C
A fraction - SN2 or SN2' cyclised chroman?

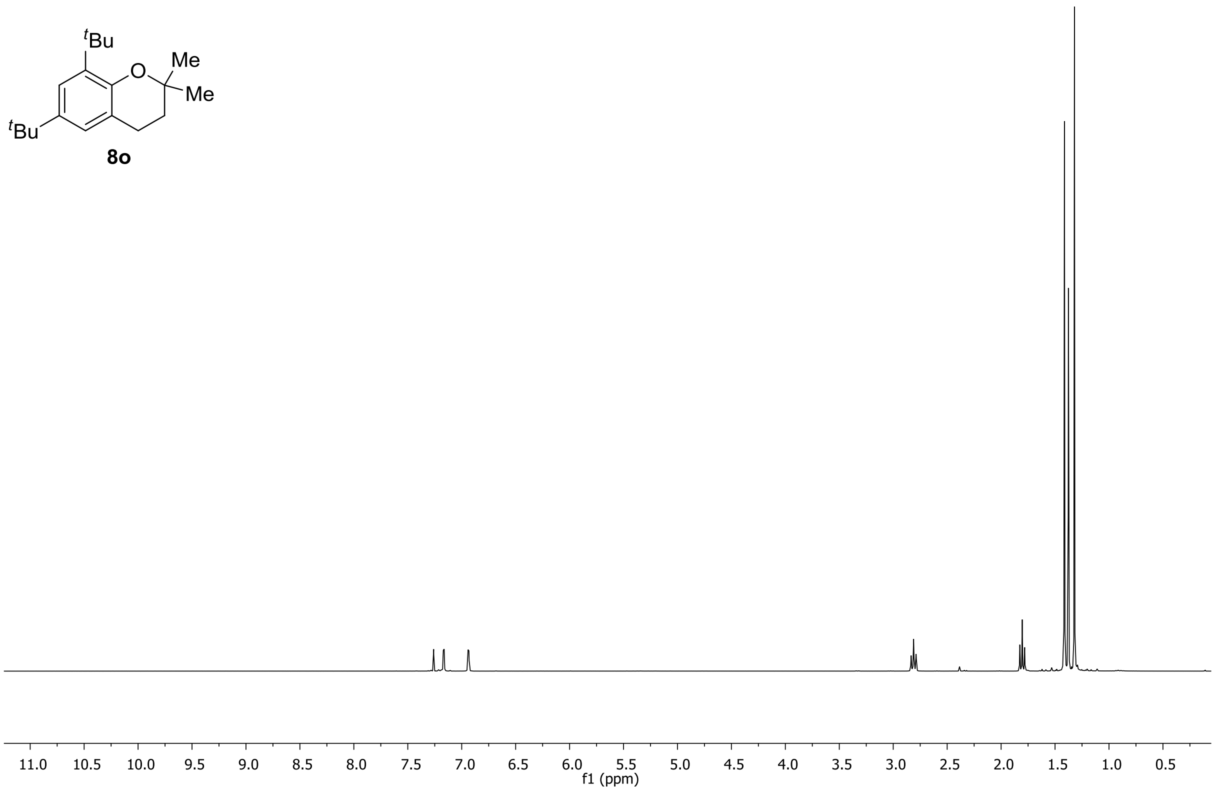

elccb069a
13C 75.5MHz Job 26559 Coutant Eloi B069A CDCl3 25.0°C 0 hour 18 min
A fraction - SN2 or SN2' cyclised chroman?

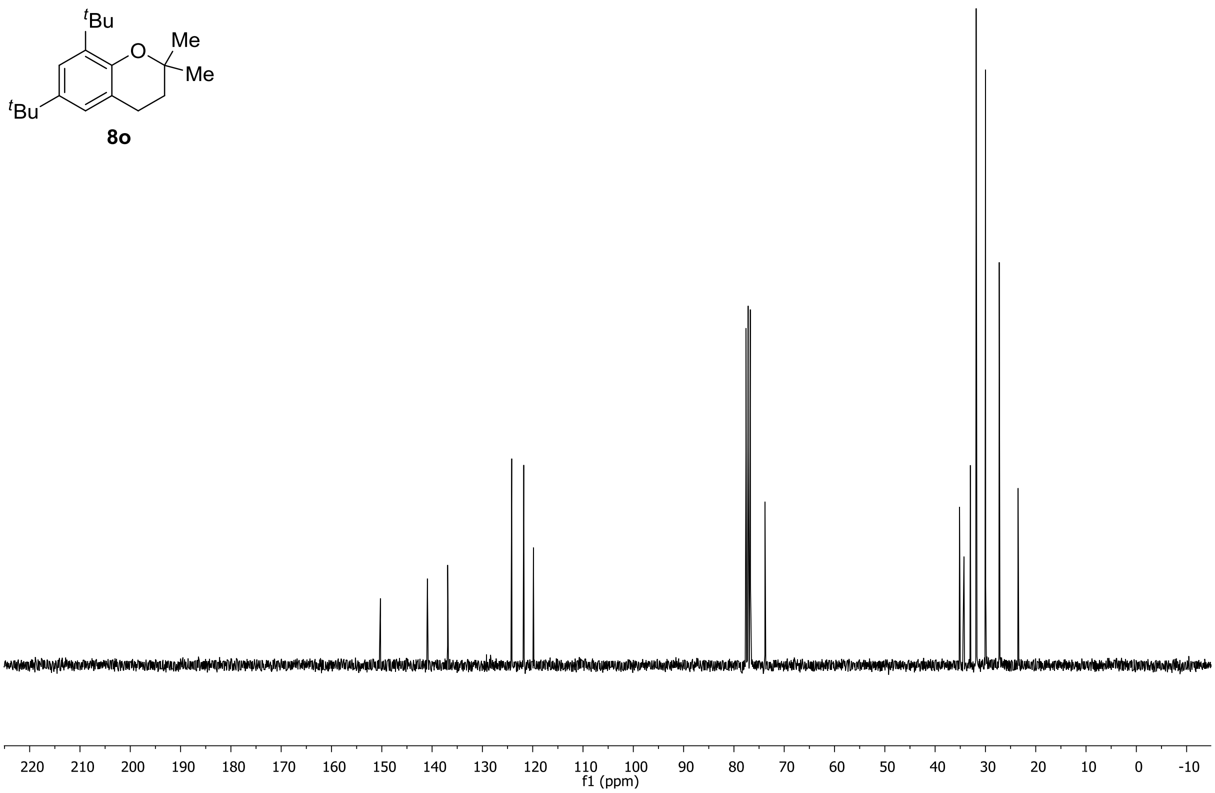

6,8-Di-*tert*-butylspiro[chroman-2,1'-cyclohexane] (8p)

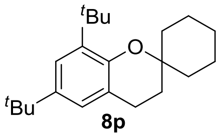

8p

1-Vinylcyclohexanol (**14**, 20.2 mg, 160 µmol) and the 2,4-di-*tert*-butylphenol (**5j**, 164 mg, 792 µmol) were dissolved in toluene (0.34 mL). PPh_3AuNTf_2 (as the 2:1 toluene adduct) (5.9 mg, 7.9 µmol) was added to the resulting solution. The reaction was allowed to stir at 60 °C for 41 hours. The reaction was then filtered over a plug of silica, using diethyl ether as eluent. The crude product was purified using column chromatography, using a gradient eluent system of neat hexane to 9:1 hexane:diethyl ether. Product **8p** was obtained as an off-white oil in approx. 85% purity (30.6 mg, 97.3 µmol, 61%).

ν_{max}/cm^{-1} 2933, 2863, 1477, 1461, 1444, 1361, 1230, 1203, 1161, 1148, 1128, 975, 944, 876, 755.

δ_H (300 MHz, $CDCl_3$) 7.17 (1H, d, *J* 2.5 Hz, Ar-H), 6.92 (1H, d, *J* 2.5 Hz, Ar-H), 2.77 (1H, t, *J* 6.8 Hz, CH_2), 1.79 (2H, t, *J* 6.8 Hz, CH_2), 1.91–1.47 (10H, m, $C(CH_2)_5$).

δ_C (75 MHz, $CDCl_3$) 150.2 (C), 140.6 (C), 136.6 (C), 124.4 (CH), 122.0 (CH), 120.3 (C), 75.3 (C), 36.0 (2 × CH_2), 35.3 (C), 34.2 (C), 31.8 (CH_3), 30.2 (CH_3), 22.8 (CH_2), 22.2 (2 × CH_2).

Found (APCI$^+$) $[M + H]^+$ 315.2678, $C_{22}H_{35}O$ requires 315.2682.

elchd061a
1H 300.1MHz Job 27360 Coutant Eloi d061a CDCl3 25.2°C

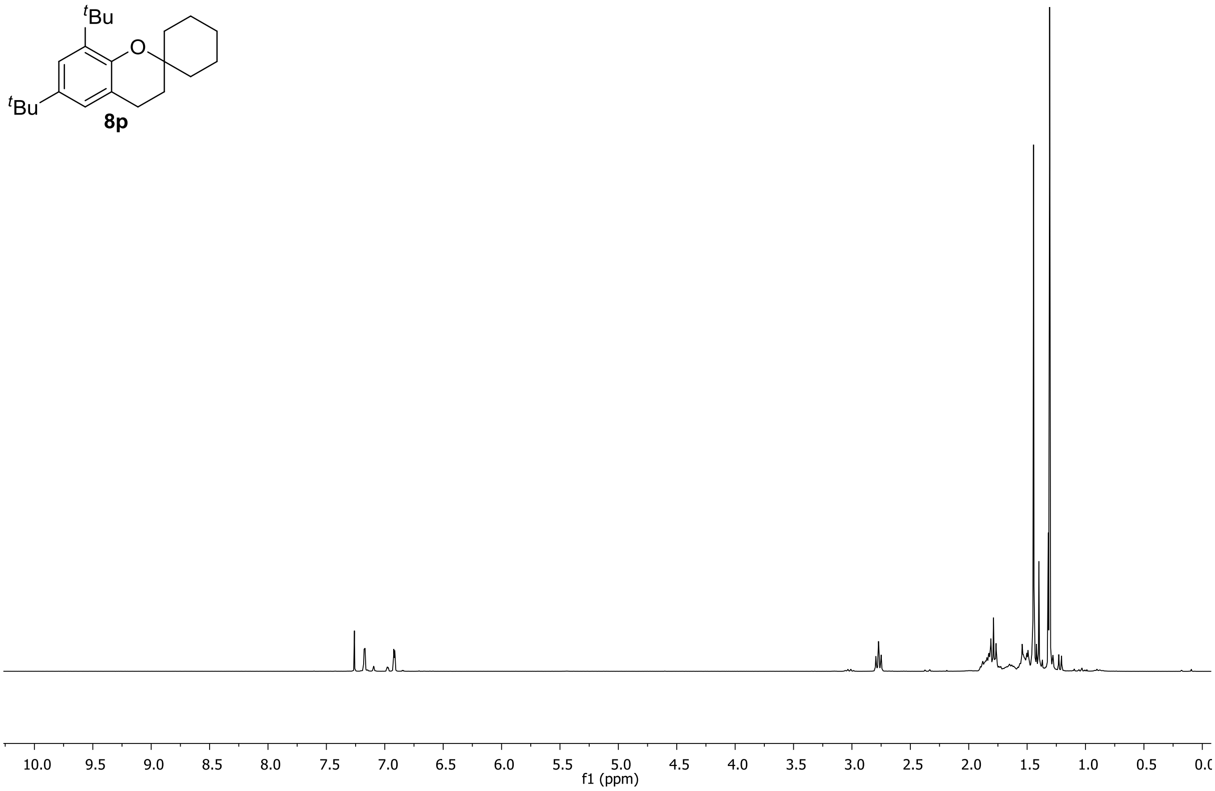

elchd061a
13C 75.5MHz Job 27363 Coutant Eloi d061a CDCl3 8 hours 16 min 25.2°C

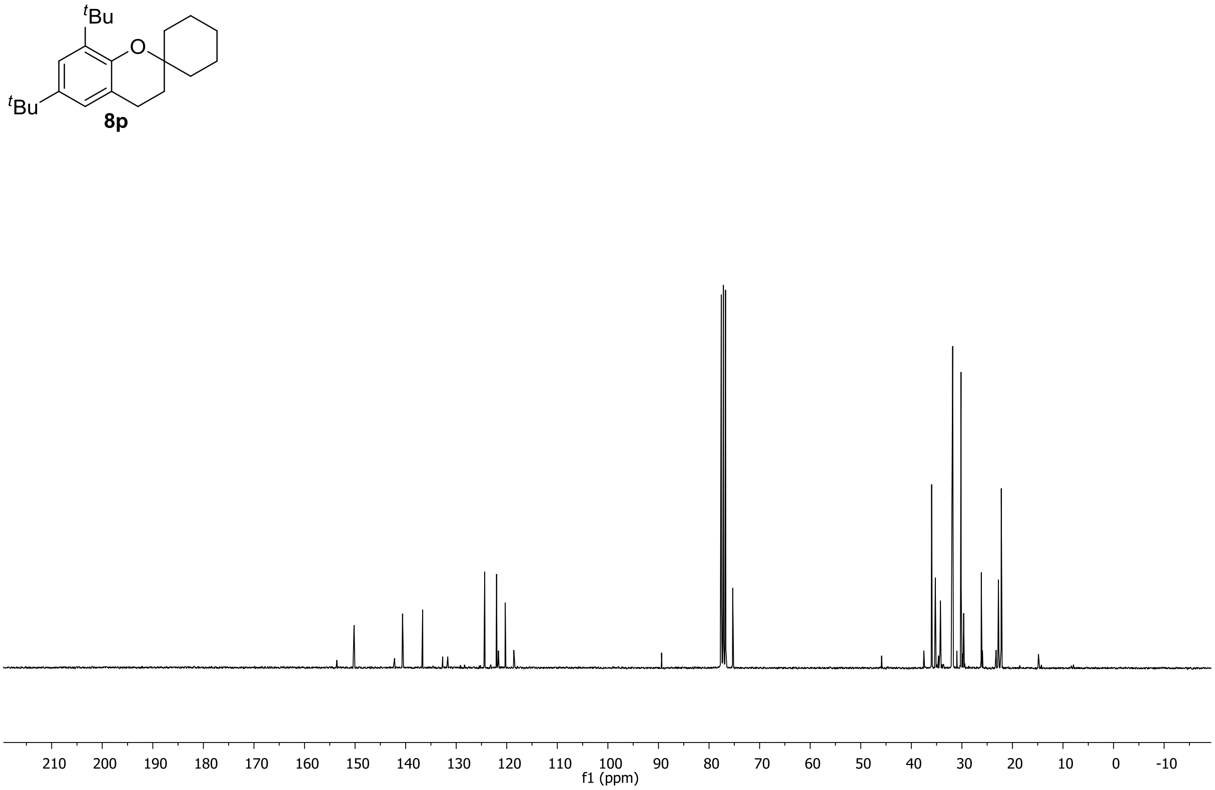

6,8-Di-*tert*-butyl-2-methyl-2-phenylchroman **(8q)**

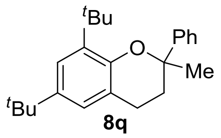

8q

2-Phenylbut-3-en-2-ol (**15**, 20.0 mg, 135 µmol) and the 2,4-di-*tert*-butylphenol (**5j**, 141 mg, 683 µmol) were dissolved in toluene (0.29 mL). PPh_3AuNTf_2 (as the 2:1 toluene adduct) (5.0 mg, 6.8 µmol) was added to the resulting solution. The reaction was allowed to stir at 70 °C for 41 hours. The reaction was then filtered over a plug of silica, using diethyl ether as eluent. The crude product was purified using column chromatography; using a gradient eluent system of neat hexane to 75:1 hexane:diethyl ether. Product **8q** was obtained as a colourless oil (30.1 mg, 89.5 µmol, 66%).

ν_{max}/cm^{-1} 2952, 2867, 1474, 1444, 1361, 1232, 1203, 1158, 1130, 1067, 946, 878, 763, 754, 698.

δ_H (300 MHz, $CDCl_3$) 7.47–7.24 (6H, m, $CC_6\underline{H_5}$), 7.23 (1H, d, *J* 2.5 Hz, Ar-$\underline{H}$), 6.87 (1H, d, *J* 2.5 Hz, Ar-H), 2.70 (1H, dt, *J* 16.3, 5.5 Hz, $C\underline{H}HCH_2$), 2.52 (1H, ddd, *J* 16.3, 8.9, 6.4 Hz, $CH\underline{H}CH_2$), 2.25 (1H, ddd, *J* 13.4, 6.4, 5.5 Hz, $CH_2C\underline{H}H$), 2.04 (1H, ddd, *J* 13.4, 8.9, 5.5 Hz, $CH_2CH\underline{H}$), 1.70 (3H, s, $C\underline{H_3}$), 1.51 (9H, s, $C(CH_3)_3$), 1.31 (9H, s, $C(CH_3)_3$).

δ_C (75 MHz, $CDCl_3$) 150.3 (C), 146.9 (C), 141.3 (C), 136.5 (C), 128.3 (CH), 126.7 (CH), 125.1 (CH), 124.2 (CH), 122.1 (CH), 120.6 (C), 79.0 (C), 35.3 (CH_2), 34.3 (C), 34.2 (C), 31.8 (CH_3), 30.2 (CH_3), 28.5 (CH_3), 23.7 (CH_2).

Found (APCI$^+$) $[M + H]^+$ 337.2525, $C_{24}H_{33}O$ requires 337.2526.

elchb076b
1H 300.1MHz Job 26890 Coutant Eloi B076B CDCl3   25.0°C
B fraction - Ph/Me AA + di(tBu)phenol, 70C, 2d

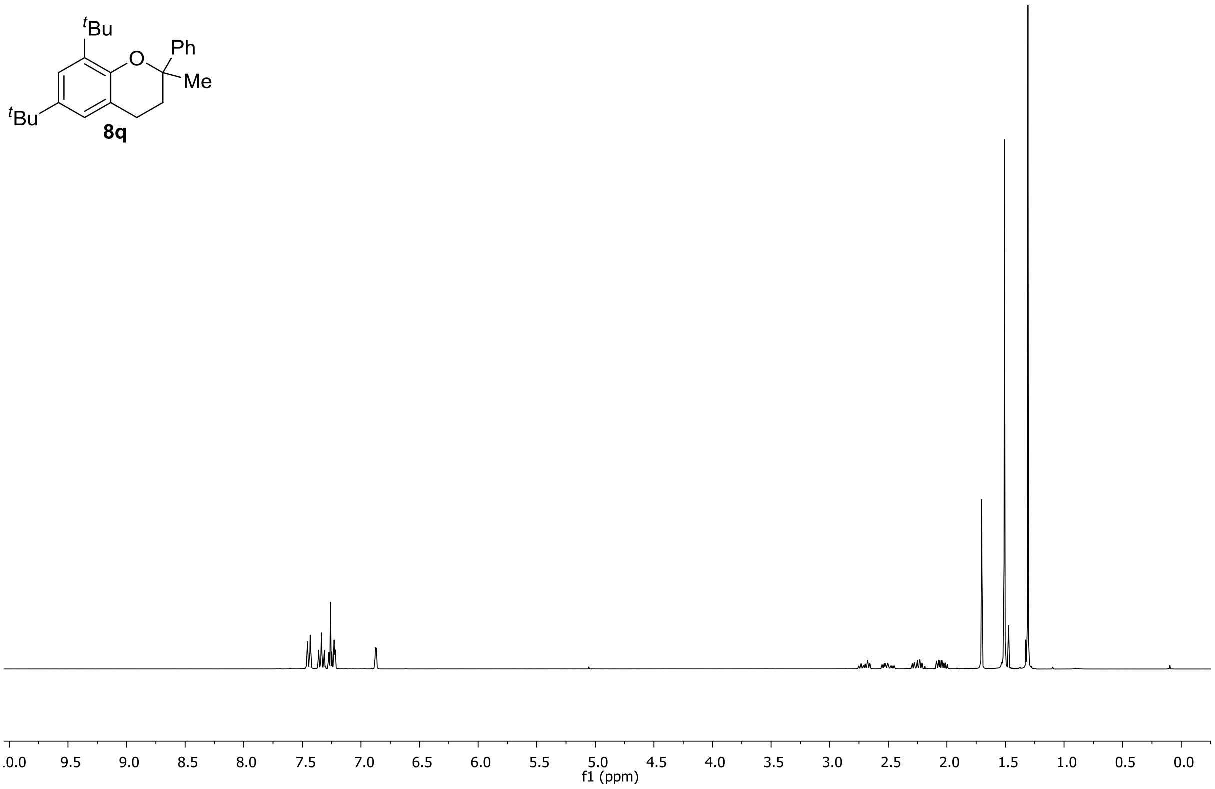

elccb076b
13C 75.5MHz Job 26904 Coutant Eloi B076B CDCl3   25.0°C   0 hour 36 min
B fraction - Ph/Me AA + di(tBu)phenol, 70C, 2d

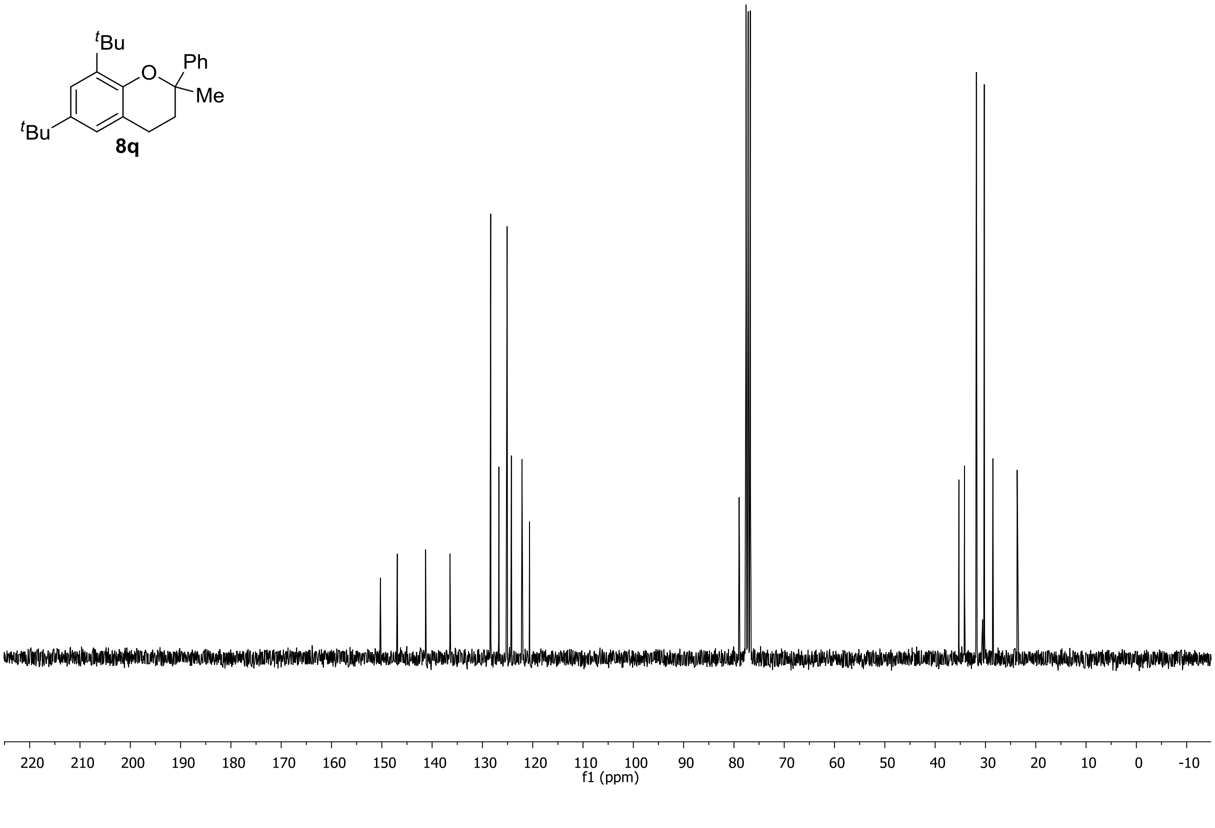

6,8-Di-*tert*-butyl-2,2-dibutyl-4-methylchroman **(8r)**

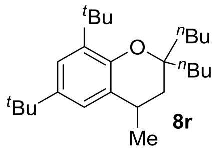

8r

5-(Prop-1-en-1-yl)nonan-5-ol (**16**, 19.8 mg, 107 µmol) and 2,4-di-*tert*-butylphenol (**5j**, 112 mg, 540 µmol) were dissolved in toluene (0.23 mL). PPh_3AuNTf_2 (as the 2:1 toluene adduct) (4.1 mg, 5.5 µmol) was added to the resulting solution. The reaction was allowed to stir at 70 °C for 43 hours. The reaction was then filtered over a plug of silica, using diethyl ether as eluent. The crude product was purified using column chromatography, using a gradient eluent system of neat hexane to 125:1 hexane:diethyl ether. Product **8r** was obtained as a colourless oil (17.9 mg, 48.0 µmol, 45%).

ν_{max}/cm^{-1} 2955, 2937, 2864, 1467, 1439, 1361, 1294, 1232, 1217, 1203, 1136, 985, 877, 756.

δ_H (300 MHz, $CDCl_3$) 7.16 (1H, d, *J* 2.4 Hz, Ar-$\underline{H}$), 7.12 (1H, d, *J* 2.4 Hz, Ar-$\underline{H}$), 3.03–2.76 (1H, m, C$\underline{H}$CH$_2$), 1.84 (1H, dd, *J* 13.5, 5.7 Hz, CHC$\underline{H}$H), 1.71–1.41 (9H, m, CHCH$\underline{H}$ and 2x(C$\underline{H_2}$)$_2$CH$_2$CH$_3$), 1.39 (9H, s, C(C$\underline{H_3}$)$_3$), 1.30 (9H, s, C(C$\underline{H_3}$)$_3$), 1.29–1.22 (4H, m, 2x(CH$_2$)$_2$C$\underline{H_2}$CH$_3$), 0.97–0.88 (6H, m, 2x(CH$_2$)$_2$CH$_2$C$\underline{H_3}$).

δ_C (75 MHz, $CDCl_3$) 149.7 (C), 140.5 (C), 136.6 (C), 125.6 (C), 122.0 (CH), 121.6 (CH), 78.5 (C), 39.4 (CH_2), 38.4 (CH_2), 35.2 (C), 34.6 (C), 34.5 (CH_2), 31.9 (CH_3), 30.1 (CH_3), 26.5 (CH_2), 26.3 (CH), 25.6 (CH_2), 23.49 (CH_2), 23.45 (CH_2), 21.1 (CH_3), 14.33 (CH_3), 14.28 (CH_3).

Found (APCI$^+$) [M + H]$^+$ 373.3464, $C_{26}H_{45}O$ requires 337.3465.

elchc068a
1H 300.1MHz Job 26511 Coutant Eloi C068A CDCl3 24.9°C
2,2-dibutyl-4-methyl-6,8-di-tert-butylchroman

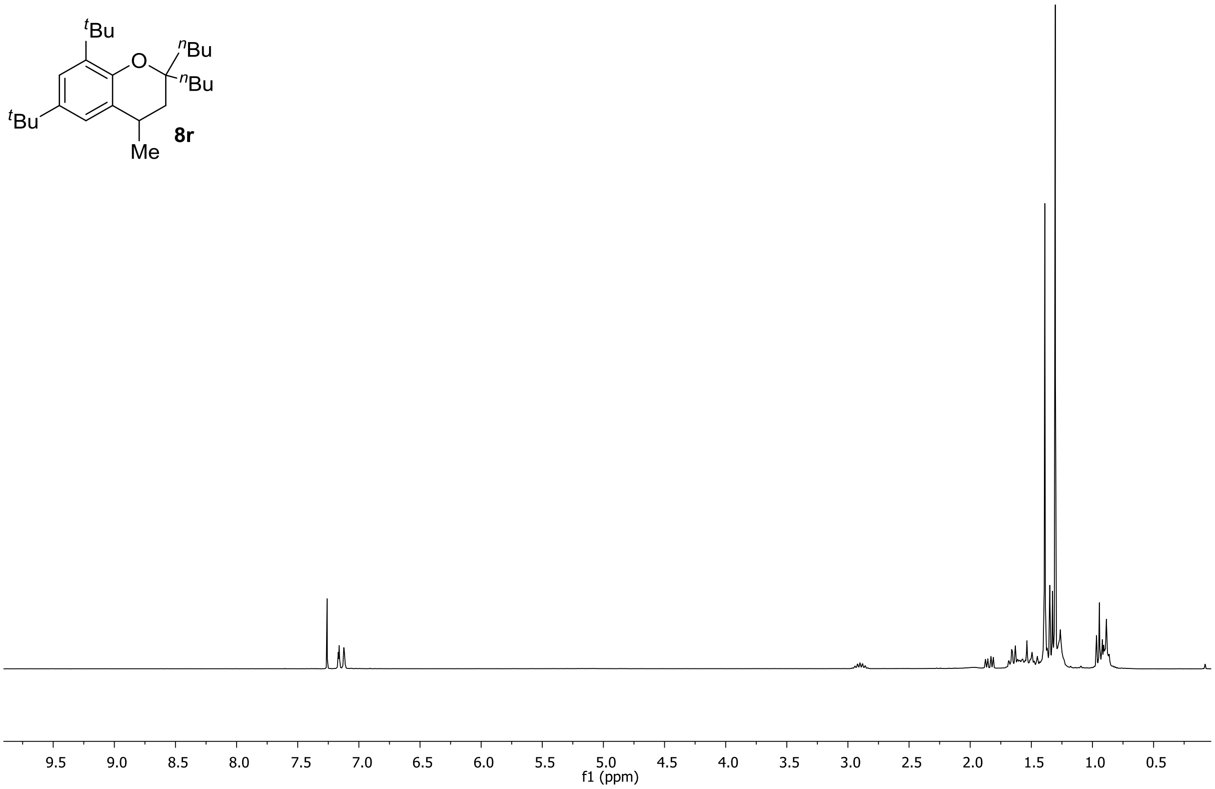

elccc068a
13C 75.5MHz Job 26520 Coutant Eloi C068A CDCl3 25.0°C 2 hours 43 min
2,2-dibutyl-4-methyl-6,8-di-tert-butylchroman

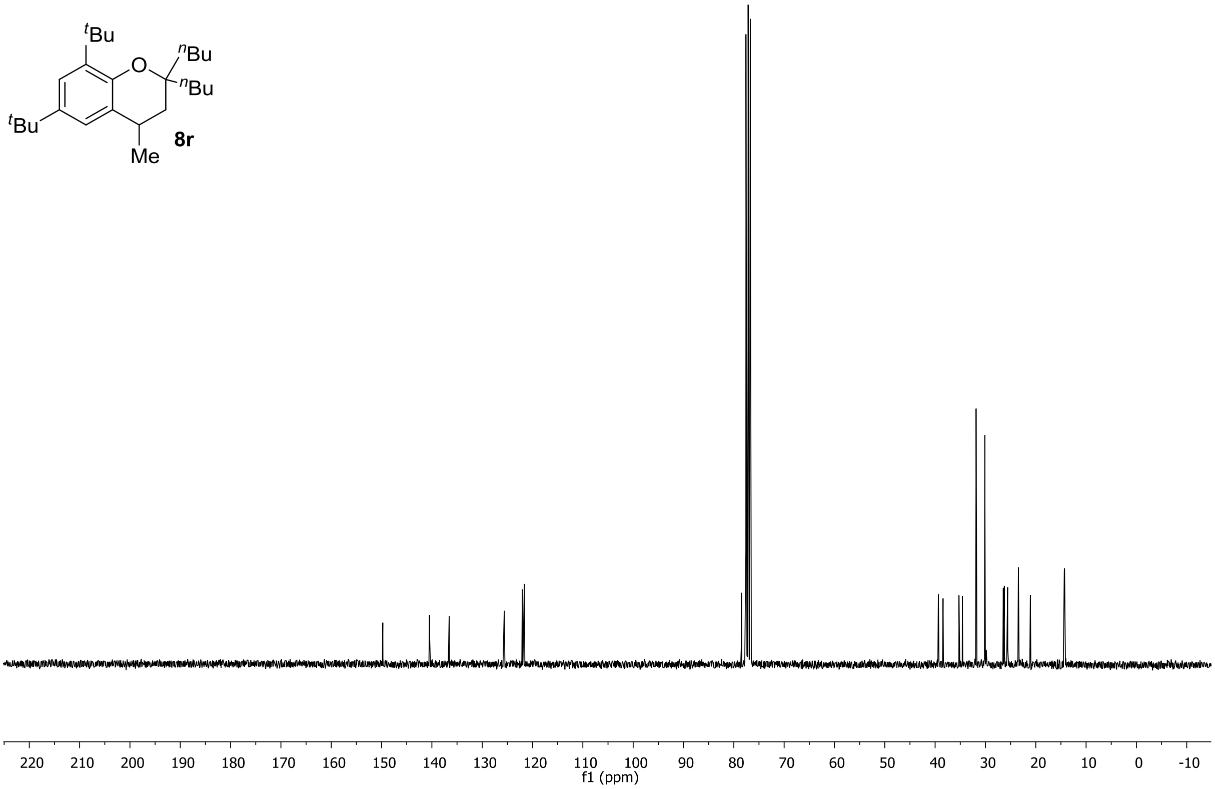

6,8-Di-*tert*-butyl-2-butyl-2,4-dimethylchroman (8s)

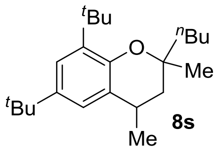

8s

4-Methyloct-2-en-4-ol (**17**, 20.6 mg, 145 µmol) and the 2,4-di-*tert*-butylphenol (**5j**, 145 mg, 704 µmol) were dissolved in toluene (0.30 mL). PPh_3AuNTf_2 (as the 2:1 toluene adduct) (5.3 mg, 7.2 µmol) was added to the resulting solution. The reaction was allowed to stir at 70 °C for 47 hours. The reaction was then filtered over a plug of silica, using diethyl ether as eluent. The crude product was isolated as an inseparable mixture of diastereoisomers by column chromatography, using a gradient eluent system of neat hexane to 125:1 hexane:diethyl ether. Product **8s** (mixture of diastereomers, approx. 2:1 ratio) was obtained as a colourless oil (22.8 mg, 69.0 µmol, 48%).

The following characterisation is on the ~2:1 diastereomeric mixture.

ν_{max}/cm^{-1} 2955, 2933, 2867, 1467, 1440, 1389, 1378, 1361, 1296, 1233, 1207, 1138, 876.

δ_H (300 MHz, $CDCl_3$) 7.23-7.12 (4H, m, Ar-H + Ar-H') 3.07–2.83 (2H, m, $(CH_3)C\underline{H}$ + $(CH_3)C\underline{H}'$), 1.95 (2H, dd, *J* 13.5, 5.8 Hz, $C\underline{H}H$ + $C\underline{H}H'$), 1.76 (2H, dd, *J* 13.5, 5.8 Hz, $CH\underline{H}$ + $CH\underline{H}'$), 1.72–1.41 (12H, m, $(C\underline{H}_2)_3CH_3$ + $(C\underline{H}_2)_3CH_3'$), 1.40 (9H, s, $C(C\underline{H}_3)_3$), 1.40 (9H, s, $C(C\underline{H}_3)_3'$), 1.39 (3H, s, $C\underline{H}_3$), 1.36 (3H, d, *J* 6.7 Hz, $CH(C\underline{H}_3)$), 1.35 (3H, d, *J* 6.7 Hz, $CH(C\underline{H}_3)'$), 1.32 (18H, s, $C(C\underline{H}_3)_3$ + $C(C\underline{H}_3)_3'$), 1.23 (3H, s, $C\underline{H}_3'$), 0.96 (3H, t, *J* 7.2 Hz, $(CH_2)_3C\underline{H}_3$), 0.90 (3H, t, *J* 7.2 Hz, $(CH_2)_3C\underline{H}_3'$).

δ_C (75 MHz, $CDCl_3$) 149.9 (C), 149.7 (C'), 140.7 (C), 140.6 (C'), 136.7 (C), 136.6 (C'), 125.6 (C'), 125.5 (C), 122.0 (CH'), 121.9 (CH), 121.7 (CH), 121.6 (CH'), 76.5 (C'), 76.0 (C), 43.3 (CH_2), 40.8 (CH_2), 40.5 (CH_2'), 37.6 (CH_2'), 35.2 (C + C'), 34.5 (C + C'), 31.9 (CH_3 + CH_3'), 30.2 (CH_3'), 30.1 (CH_3), 27.3 (CH_3'), 26.8 (CH_2'), 26.7 (CH), 26.6 (CH'), 26.0 (CH_2), 23.5 (CH_2 + CH_2'), 22.6 (CH_3), 21.1 (CH_3' + CH_3), 14.3 (CH_3' + CH_3).

Found (APCI$^+$) $[M + H]^+$ 331.2994, $C_{23}H_{39}O$ requires 331.2995.

elchb073a
1H 300.1MHz Job 26722 Coutant Eloi B073A CDCl3 25.0°C
2-butyl-2-methyl-6,8-di-tert-butylchroman ?

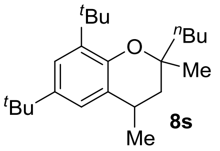

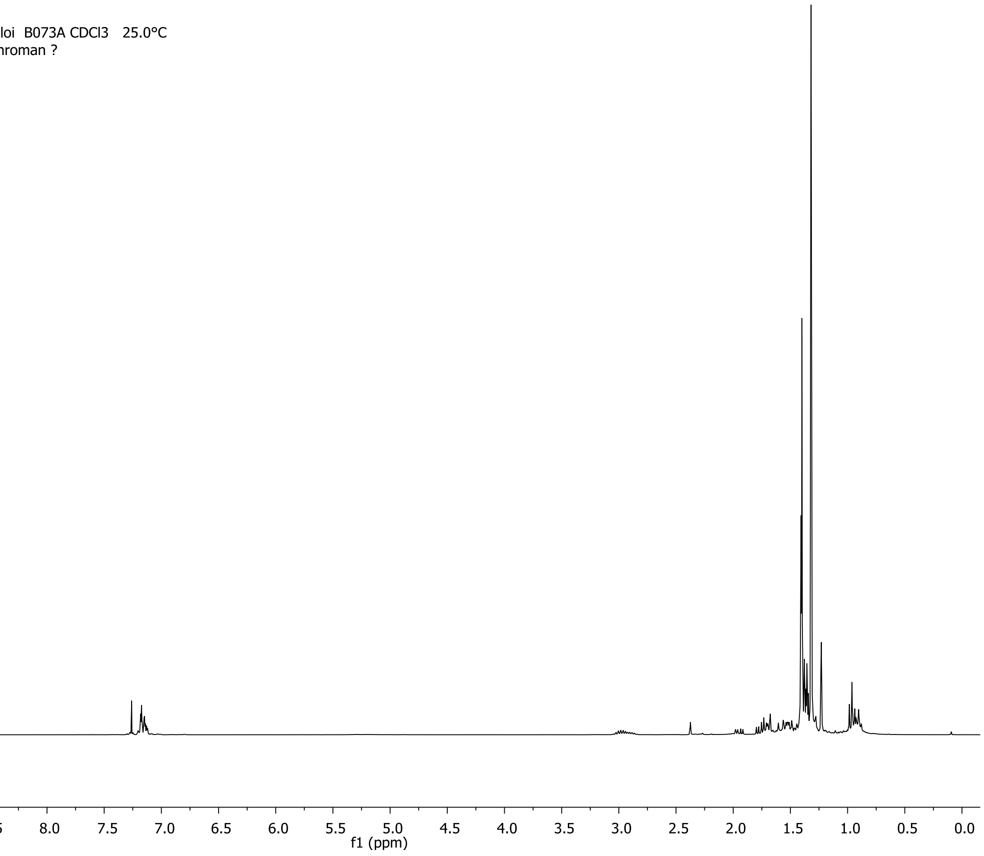

elccb073a
13C 75.5MHz Job 26739 Coutant Eloi B073A CDCl3 25.0°C 0 hour 36 min
2-butyl-2-methyl-6,8-di-tert-butylchroman ?

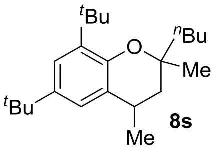

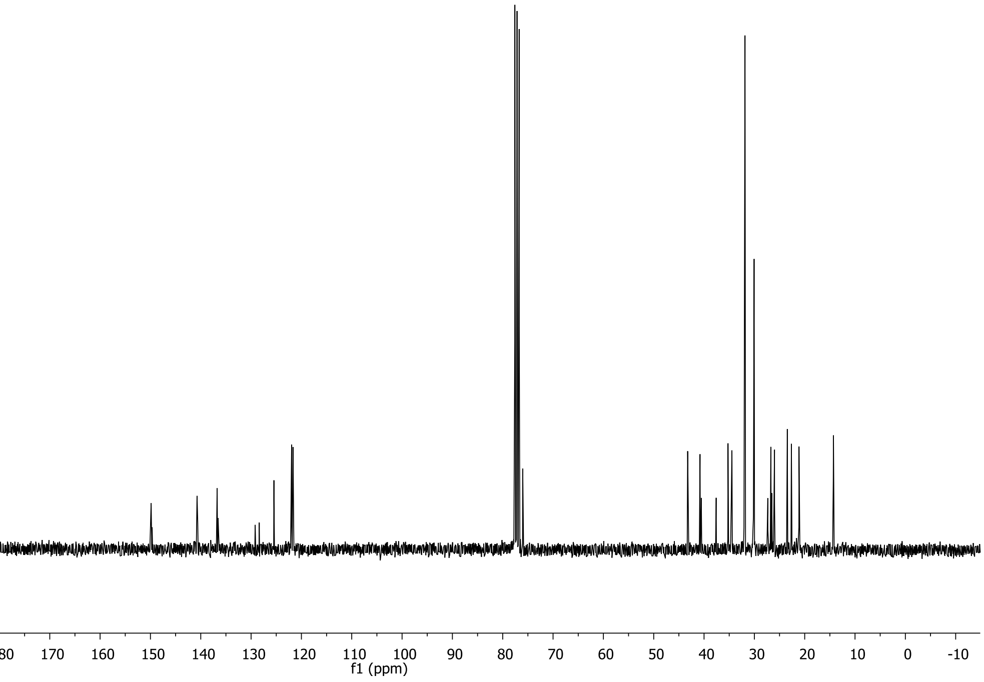

2,4-Di-*tert*-butyl-6-(3-butyl-2-methylhept-2-en-1-yl)phenol (9t)

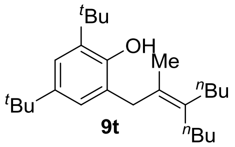

9t

5-(Prop-1-en-2-yl)nonan-5-ol (**18**, 19.5 mg, 106 µmol) and 2,4-di-*tert*-butylphenol (**5j**, 112 mg, 540 µmol) were dissolved in toluene (0.23 mL). PPh_3AuNTf_2 (as the 2:1 toluene adduct) (4.0 mg, 5.4 µmol) was added to the resulting solution. The reaction was allowed to stir at 60 °C for 42 hours. The reaction was then filtered over a plug of silica, using diethyl ether as eluent. The crude product was purified using column chromatography, using a gradient eluent system of neat hexane to 10:1 hexane:diethyl ether. Product **9t** was obtained as a colourless oil (13.6 mg, 36.5 µmol, 35%).

ν_{max}/cm^{-1} 3445, 2956, 2933, 2871, 1479, 1459, 1362, 1216, 1201, 756, 735.

δ_H (300 MHz, $CDCl_3$) 7.17 (1H, d, *J* 2.5 Hz, Ar-H), 6.94 (1H, d, *J* 2.5 Hz, Ar-H), 5.48 (1H, s, O-H), 3.42 (2H, s, $C\underline{H_2}C(CH_3)$=C), 2.26–2.19 (2H, m, $=CC\underline{H_2}(CH_2)_2CH_3$), 2.15–2.08 (2H, m, $=CC\underline{H_2}(CH_2)_2CH_3$), 1.57 (3H, s, $=CC\underline{H_3}$), 1.40 (9H, s, $C(C\underline{H_3})_3$), 1.45–1.27 (8H, m, $2xCH_2(C\underline{H_2})_2CH_3$), 1.29 (9H, s, $C(C\underline{H_3})_3$), 0.93 (3H, t, *J* 7.0 Hz, $(CH_2)_3C\underline{H_3}$), 0.92 (3H, t, *J* 7.1 Hz, $(CH_2)_3C\underline{H_3}$).

δ_C (75 MHz, $CDCl_3$) 151.9 (C), 141.8 (C), 138.2 (C), 135.4 (C), 126.4 (C), 125.4 (CH), 125.0 (C), 122.1 (CH), 36.8 (CH_2), 35.0 (C), 34.4 (C), 32.6 (CH_2), 32.4 (CH_2), 31.8 (CH_3), 31.4 (CH_2), 31.0 (CH_2), 29.9 (CH_3), 23.2 (CH_2), 23.1 (CH_2), 17.5 (CH_3), 14.2 (2 × CH_3).

Found (APCI$^+$) $[M - H]^+$ 371.3302, $C_{26}H_{43}O$ requires 371.3308.

elchb067b
1H 300.1MHz Job 26549 Coutant Eloi B067B CDCl3 24.8°C
B fraction - Bu/Bu beta-methyl-substituted AA

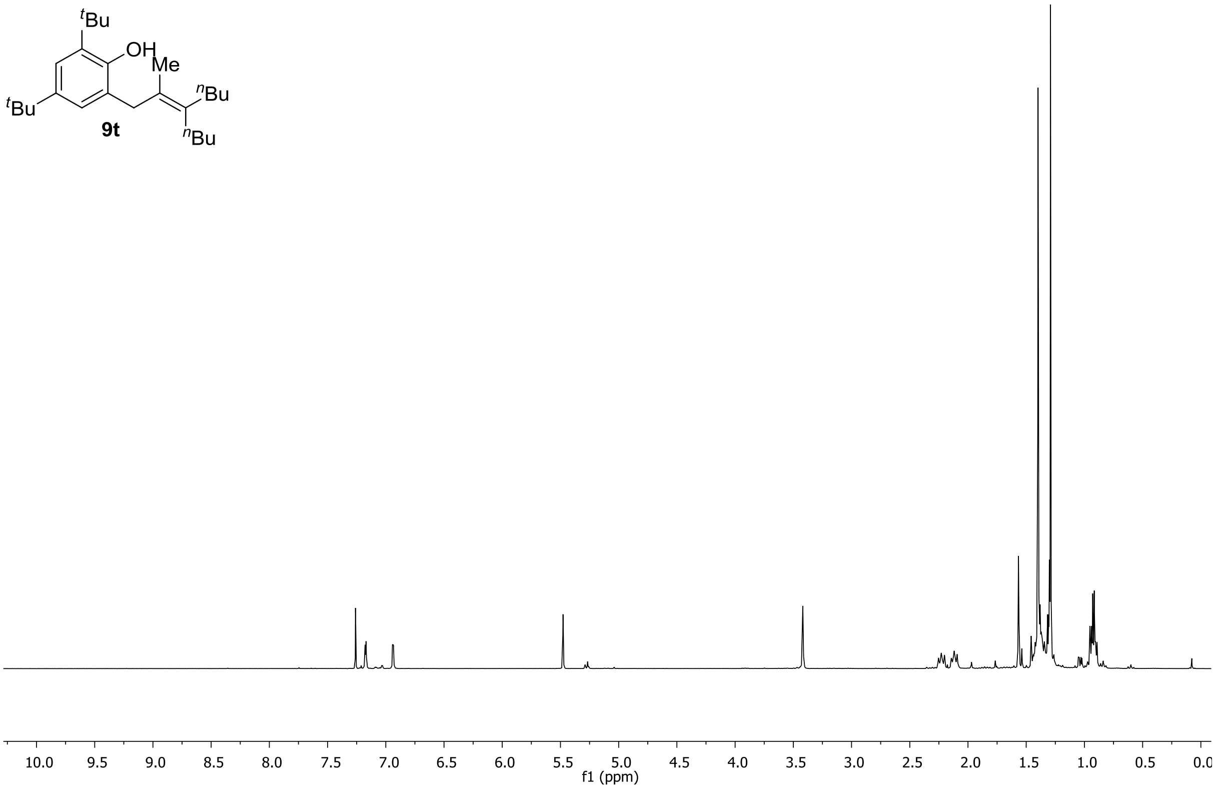

elccb067b
13C 75.5MHz Job 26558 Coutant Eloi B067B CDCl3 25.0°C 2 hours 7 min
B fraction - Bu/Bu beta-methyl-substituted AA

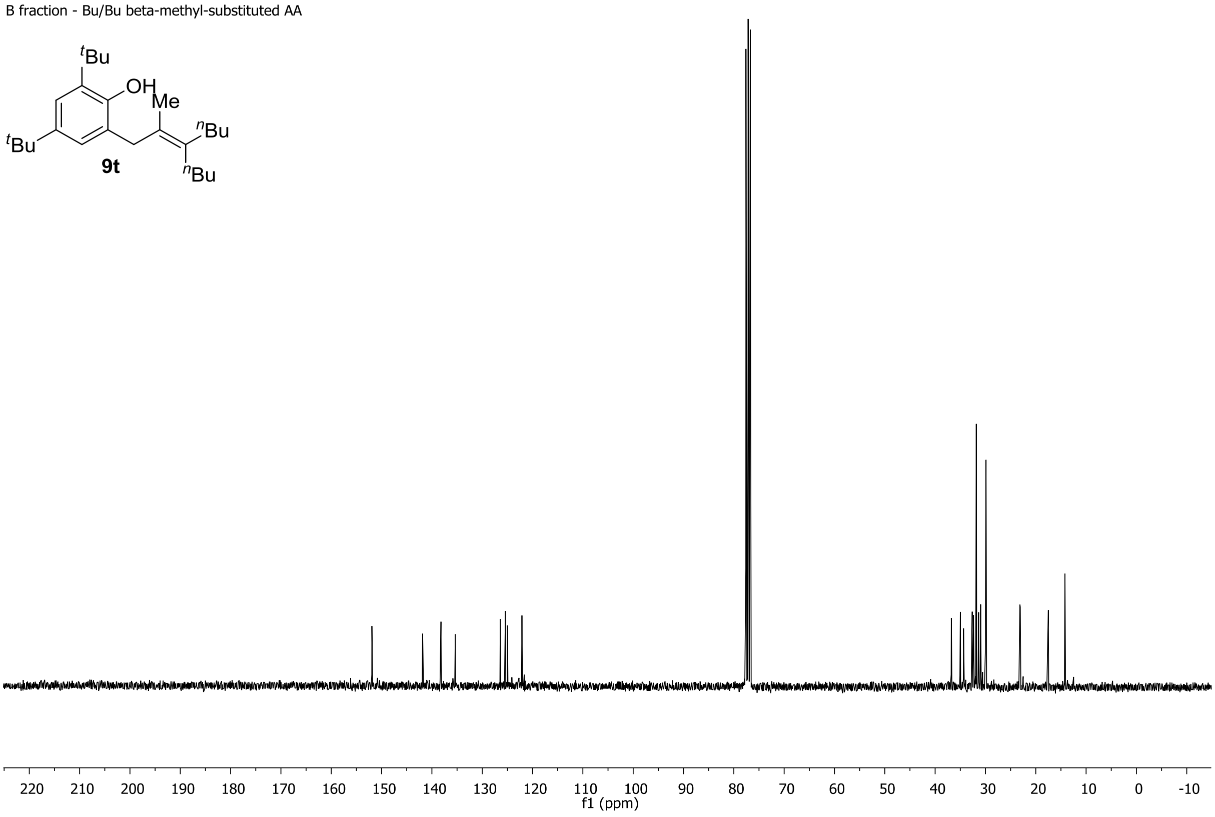

6,8-Di-*tert*-butyl-2,2,4-trimethylchroman (8u)

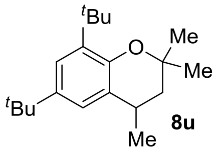

4-Methylprop-3-en-2-ol (**20**, 9.8 mg, 97.8 µmol) and the 2,4-di-*tert*-butylphenol (**5j**, 105 mg, 509 µmol) were dissolved in toluene (0.21 mL). PPh_3AuNTf_2 (as the 2:1 toluene adduct) (3.6 mg, 4.9 µmol) was added to the resulting solution. The reaction was allowed to stir at 70 °C for 42 hours. The reaction was then filtered over a plug of silica, using diethyl ether as eluent. The crude product was purified using column chromatography, using neat hexane as eluent. Product **8u** was obtained as a colourless oil (14.3 mg, 49.6 µmol, 51%).

ν_{max}/cm^{-1} 2955, 2929, 2909, 2868, 1455, 1440, 1382, 1361, 1237, 1225, 1213, 1142, 947, 876.

δ_H (300 MHz, $CDCl_3$) 7.18–7.15 (1H, m, Ar-H), 7.13 (1H, dd, *J* 2.5, 1.0 Hz, Ar-H), 3.03–2.89 (1H, m, CH), 1.82 (1H, dd, *J* 13.3, 6.1 Hz, CHH), 1.55 (1H, app. t, *J* 13.3 Hz, CHH), 1.44 (3H, s, CH_3), 1.39 (9H, s, $C(CH_3)_3$), 1.36 (3H, d, *J* 6.7 Hz, $CH(CH_3)$), 1.31 (9H, s, $C(CH_3)_3$), 1.26 (3H, s, CH_3).

δ_C (75 MHz, $CDCl_3$) 149.8 (C), 140.8 (C), 136.7 (C), 125.4 (C), 121.9 (CH), 121.7 (CH), 73.8 (C), 42.9 (CH_2), 35.2 (C), 34.5 (C), 31.9 (CH_3), 30.4 (CH_3), 30.0 (CH_3), 27.0 (CH), 24.8 (CH_3), 21.1 (CH_3).

Found (APCI$^+$) $[M]^+$ 288.2444, $C_{20}H_{32}O$ requires 288.2448.

elcha088a
1H 300.1MHz Job 27481 Coutant Eloi A088A CDCl3 25.0°C
6,8-di(tBu)-2,2,4-trimethylchroman?

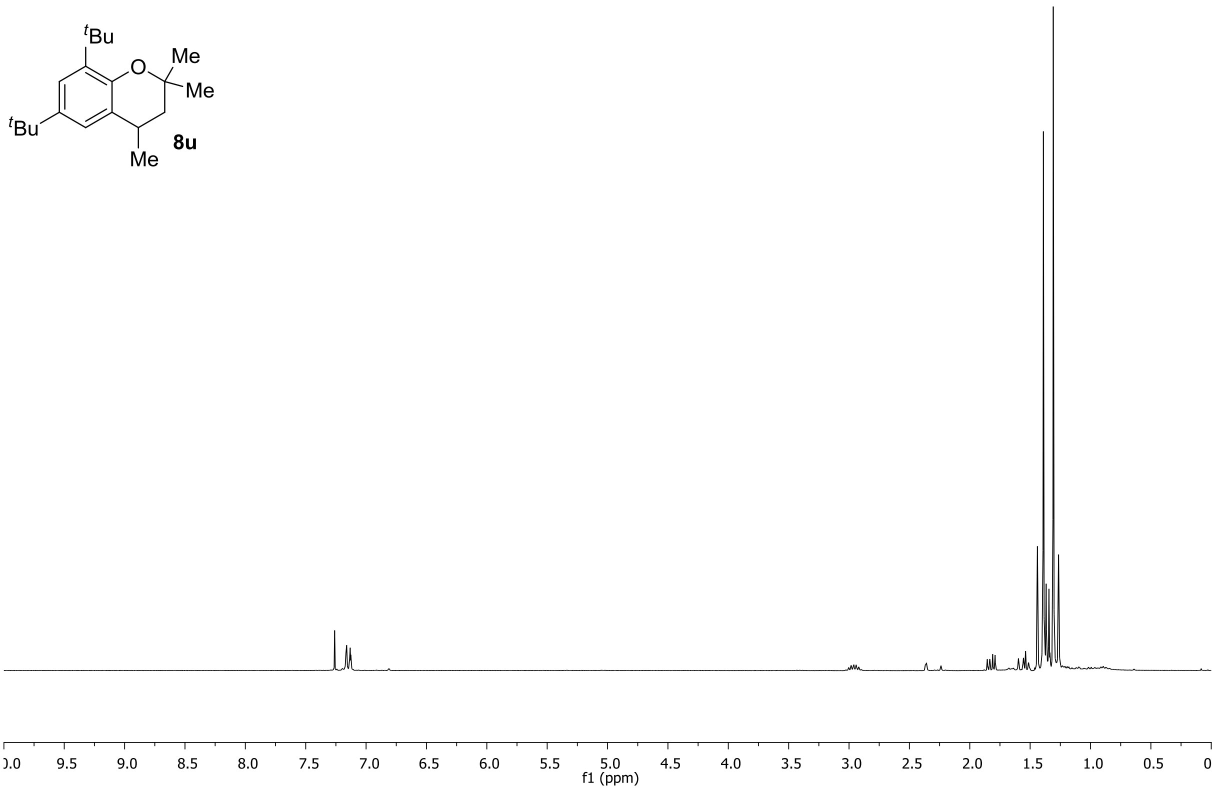

elcca088a
13C 75.5MHz Job 27486 Coutant Eloi A088A CDCl3 25.0°C 1 hour 12 min
6,8-di(tBu)-2,2,4-trimethylchroman

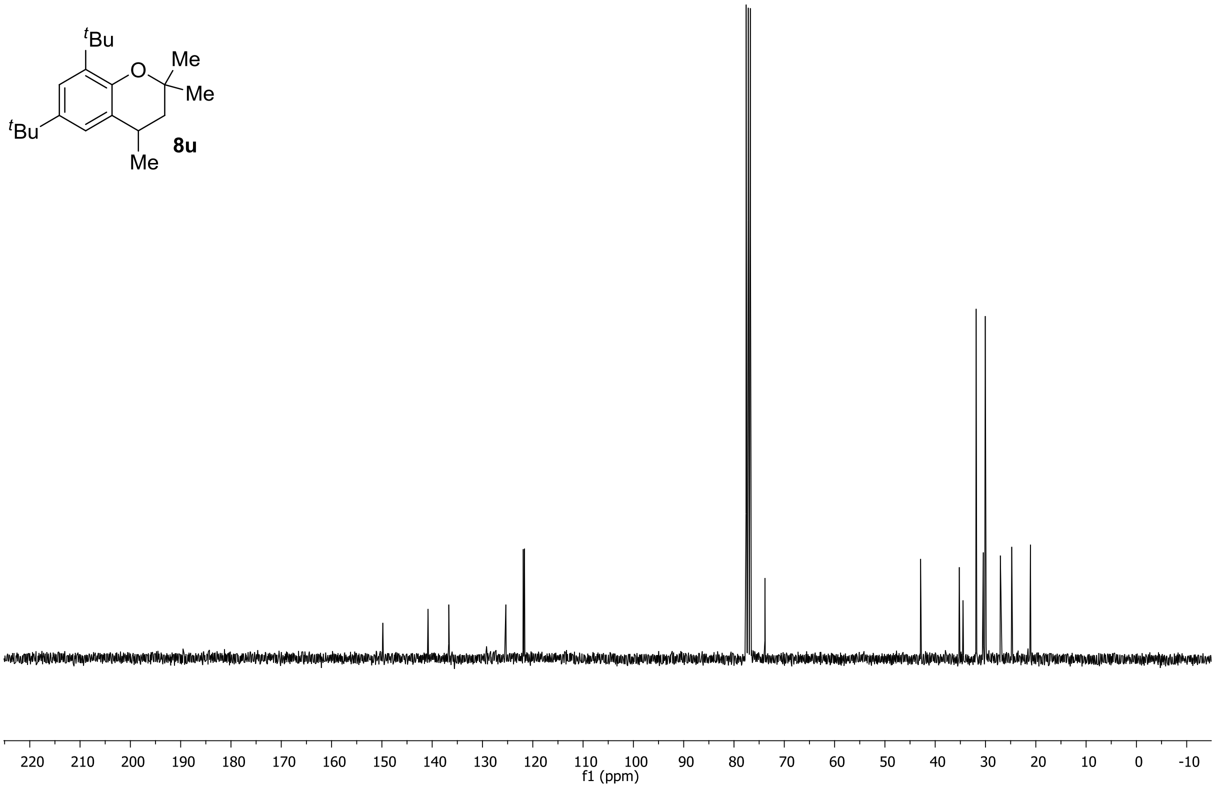

2,4-Di-*tert*-butyl-6-cinnamylphenol (9v)

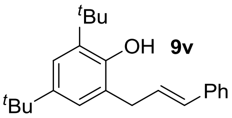

9v

2-Methyl-1-phenylprop-2-en-1-ol (**21**, 15.2 mg, 113 µmol) and the 2,4-di-*tert*-butylphenol (**5j**, 115 mg, 559 µmol) were dissolved in toluene (0.23 mL). PPh_3AuNTf_2 (as the 2:1 toluene adduct) (4.2 mg, 5.7 µmol) was added to the resulting solution. The reaction was allowed to stir at 60 °C for 41 hours. The reaction was then filtered over a plug of silica, using diethyl ether as eluent. The crude product was purified using column chromatography, using a gradient eluent system of 50:1 hexane:diethyl ether to 9:1 hexane:diethyl ether. Product **9v** was obtained as a yellow oil (27.1 mg, 84.0 µmol, 74%).

ν_{max}/cm^{-1} 3525, 2955, 2905, 2868, 1479, 1448, 1362, 1215, 1199, 972, 878, 755, 692.

δ_H (300 MHz, $CDCl_3$) 7.43–7.25 (6H, m, Ph-H and Ar-H), 7.07 (1H, d, *J* 2.5 Hz, Ar-H), 6.63 (1H, d, *J* 15.9 Hz, =CHPh), 6.42 (1H, dt, *J* 15.9 Hz, 6.5, =CH), 5.13 (1H, s, OH), 3.60 (2H, dd, *J* 6.5, 1.3 Hz, CH_2), 1.44 (9H, s, $C(CH_3)_3$), 1.34 (9H, s, $C(CH_3)_3$).

δ_C (75 MHz, $CDCl_3$) 151.4 (C), 142.7 (C), 136.8 (C), 136.1 (C), 132.1 (CH), 128.7 (CH), 128.1 (CH), 127.7 (CH), 126.5 (CH), 125.2 (CH), 124.6 (C), 123.0 (CH), 36.1 (CH_2), 35.0 (C), 34.4 (C), 31.8 (CH_3), 30.0 (CH_3).

Found ($APCI^+$) $[M + NH_4]^+$ 340.2630, $C_{23}H_{34}ON$ requires 340.2635.

elcha064a
1H 300.1MHz Job 26258 Coutant Eloi A064A CDCl3 25.0°C
A fraction (3 prod) - 1-phenylprop-2-enol + di(tBu)phenol 60C 2d

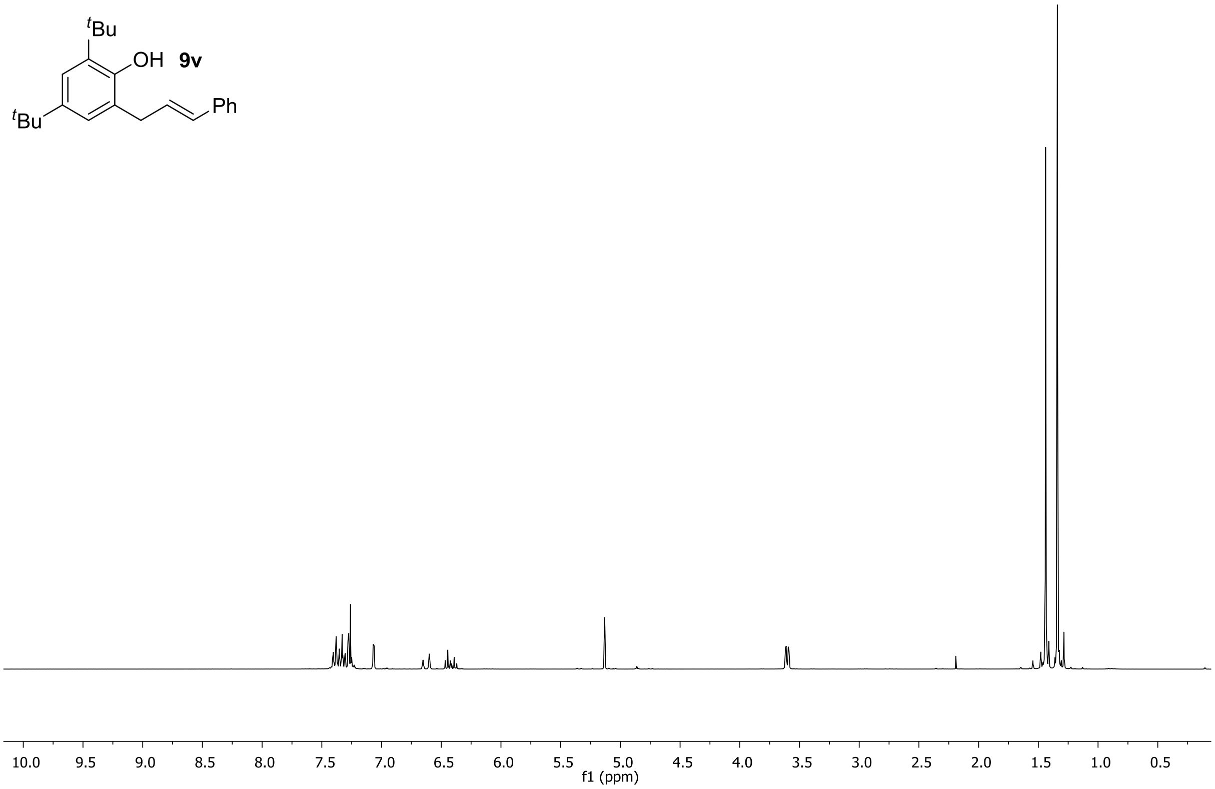

elccb064a
13C 75.5MHz Job 26905 Coutant Eloi B064A CDCl3 25.0°C 0 hour 54 min
Ph/H AA - FC Sn2' product

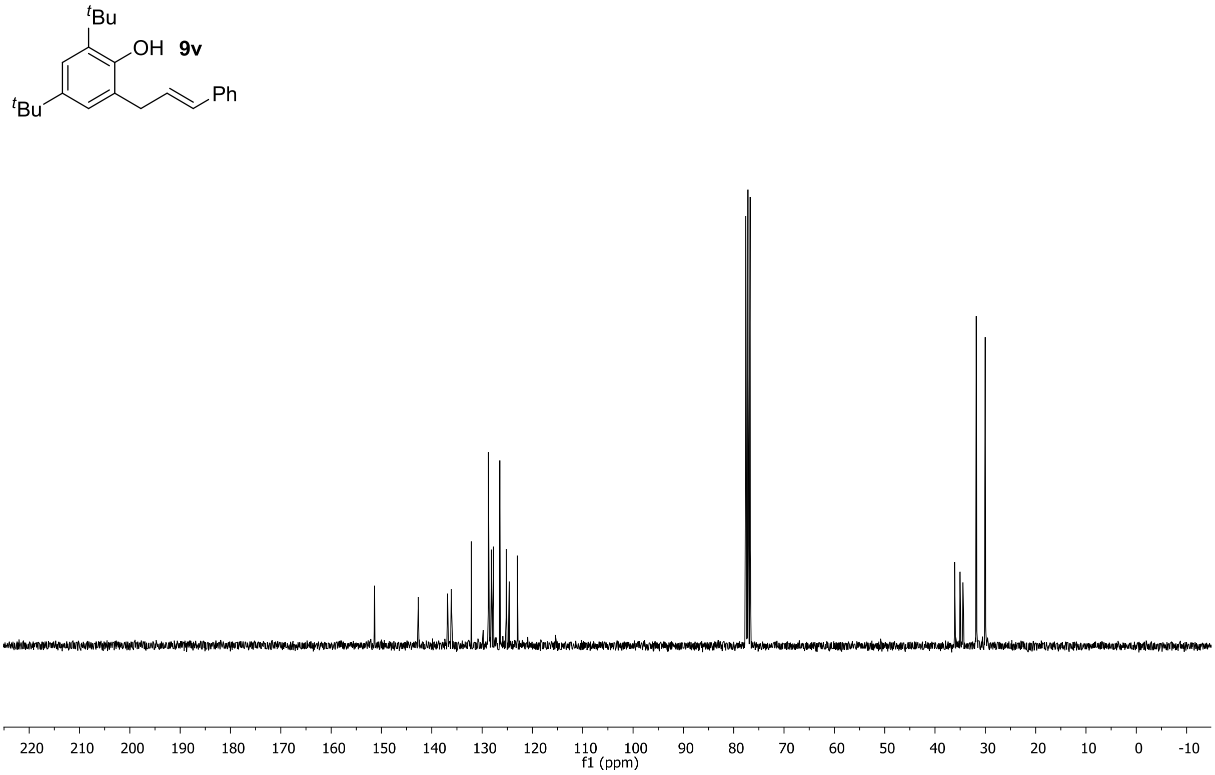

6,8-Di-*tert*-butyl-2-phenylchroman **(8v)**

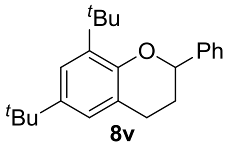

8v

6,8-Di-*tert*-butyl-2-phenylchroman (**20**, 15.1 mg, 112.5 µmol) and 2,4-di-*tert*-butylphenol (**5j**, 115.4 mg, 559 µmol) were dissolved in toluene (0.10 mL). PPh_3AuNTf_2 (as the 2:1 toluene adduct) (4.1 mg, 5.5 µmol) was added to the resulting solution. $HNTf_2$ (1.6 mg, 5.7 µmol) was measured out in a glove box, then dissolved in toluene (0.13 mL), and added to the reaction vial. The reaction was allowed to stir at 60 °C for 41 hours. The reaction was then filtered through a plug of silica, using diethyl ether as the eluent. The crude mixture was purified using column chromatography, using neat hexane. The isolated material was impure, so a plug of silica was used to further purify the product. The impurity was washed off in neat hexane, and the desired product was obtained after an ether wash. Product **8v** was obtained as a colourless oil (18.7 mg, 58.0 µmol, 52%).

ν_{max}/cm^{-1} 2952, 2867, 1496, 1476, 1444, 1361, 1231, 1127, 877, 752, 697.

δ_H (300 MHz, $CDCl_3$) 7.53–7.27 (5H, m, C($C_6\underline{H_5}$)), 7.20 (1H, d, *J* 2.5 Hz, Ar-$\underline{H}$), 6.98 (1H, d, *J* 2.4 Hz, Ar-$\underline{H}$), 5.02 (1H, dd, *J* 10.5, 2.4 Hz, OC$\underline{H}$), 3.08 (1H, ddd, *J* 16.5, 11.8, 6.5 Hz, CC$\underline{H}$HCH_2), 2.85 (1H, ddd, *J* 16.5, 5.5, 2.6 Hz, CCH$\underline{H}$CH_2), 2.26–1.98 (2H, m, CHC$\underline{H_2}$CH_2), 1.40 (9H, s, C(C$\underline{H_3}$)$_3$), 1.32 (9H, s, C(C$\underline{H_3}$)$_3$).

δ_C (75 MHz, $CDCl_3$) 151.8 (C), 142.6 (C), 142.1 (C), 137.2 (C), 128.5 (CH), 127.7 (CH), 126.1 (CH), 124.4 (CH), 122.0 (CH), 121.2 (C), 77.9 (CH), 35.2 (C), 34.4 (C), 31.8 (CH_3), 30.8 (CH_2), 30.1 (CH_3), 26.4 (CH_2).

Found (APCI$^+$) [M – H]$^+$ 321.2214, $C_{23}H_{29}O$ requires 321.2213.

pcyhd7531H 300.1MHz Job 29296 Young Paul C D753 CDCl3 24.9°C
Purified

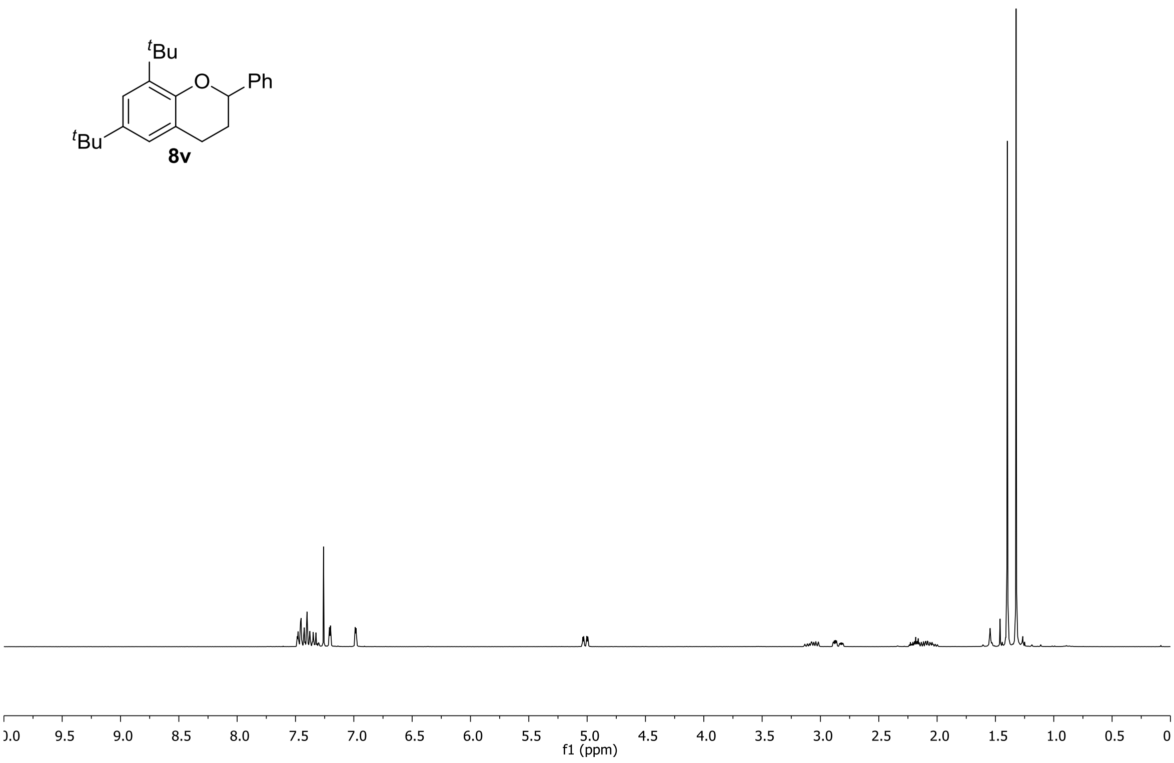

pcycd75313C 75.5MHz Job 29317 Young Paul C D753 CDCl3 25.0°C 2 hours 7 min
Purified

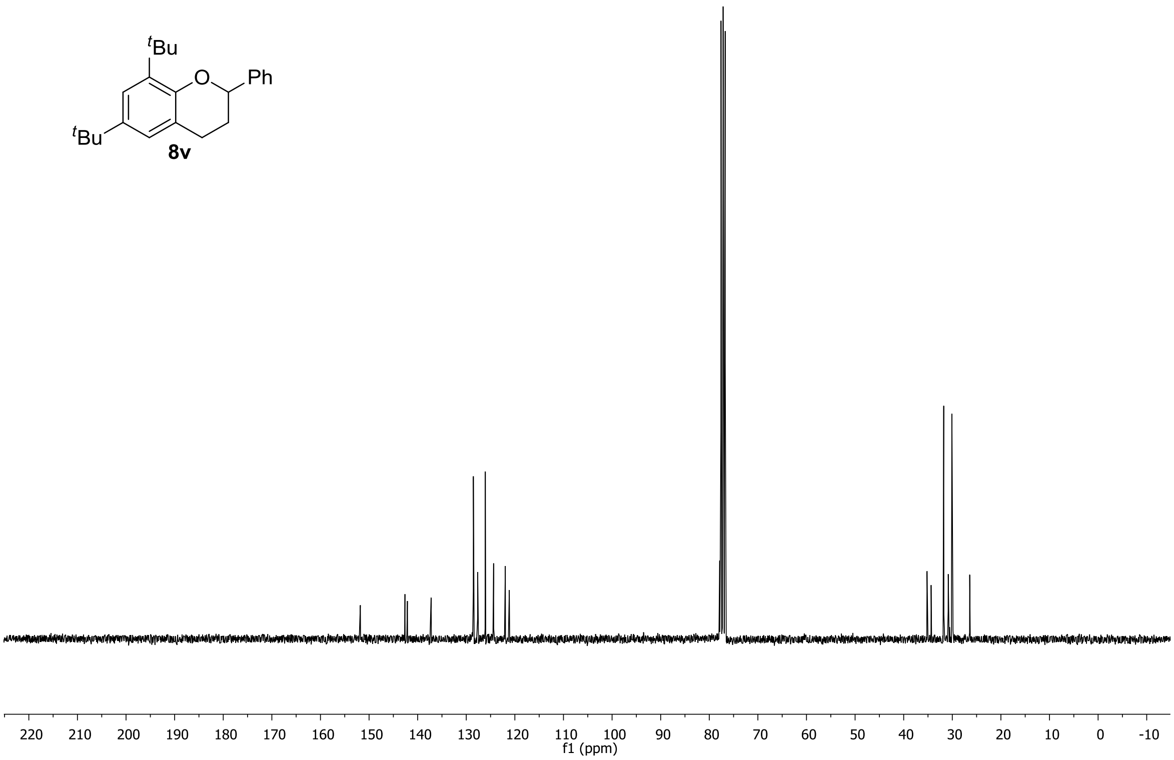

As noted in footnote 77 of the article, a minor rearrangement side product is often observed in 15-25% yields when the allylic alcohol **7** is used as a substrate (noted by the appearance of the distinctive multiplet at ~5.4–5.5 ppm in the ^{1}H-NMR spectra of the crude mixture). As the desired chroman products **8a-l** were still obtained in good yields, the minor side product was not isolated, except in the case of Entry 3, Table 2 (using phenol **5b**), and appears to be a rearrangement product of **9b** (**SI-1b**).

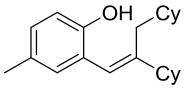

SI-1b

ν_{max}/cm^{-1} 3410, 2912, 2851, 1508, 1447, 1260, 1202, 1181, 1102, 807.

δ_H (400 MHz, $CDCl_3$) 6.90-6.84 (2H, m, Ar-H), 6.66 (1H, d, *J* 8.0 Hz, Ar-H), 5.46–5.40 (1H, m, =CH), 4.53 (1H, s, OH), 2.53–2.45 (1H, m, C$\underline{H}$H), 2.35–2.26 (1H, m, CH$\underline{H}$), 2.25 (3H, s, CH_3), 2.11–2.01 (2H, m, CH_2), 1.97–0.73 (20H, m, Cy-H).

δ_C (101 MHz, $CDCl_3$) 151.3 (C), 139.0 (C), 130.7 (CH), 130.0 (C), 128.6 (C), 127.5 (CH), 123.9 (CH), 115.3 (CH), 53.9 (CH), 40.2 (CH), 32.1 (CH_2), 31.4 (CH_2), 30.2 (CH_2), 28.3 (CH_2), 26.90 (CH_2), 26.86 (CH_2), 26.77 (CH_2), 25.53 (CH_2), 25.46 (CH_2), 23.3 (CH_2), 23.1 (CH_2), 20.7 (CH_3). Found (APCI$^+$) $[M + H]^+$ 313.2530, $C_{22}H_{33}O$ requires 313.2526.

elchd023b
1H 400.1MHz Job 20038 Coutant Eloi D023B CDCl3 25.0°C
*

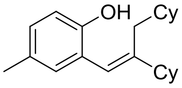

SI-1b

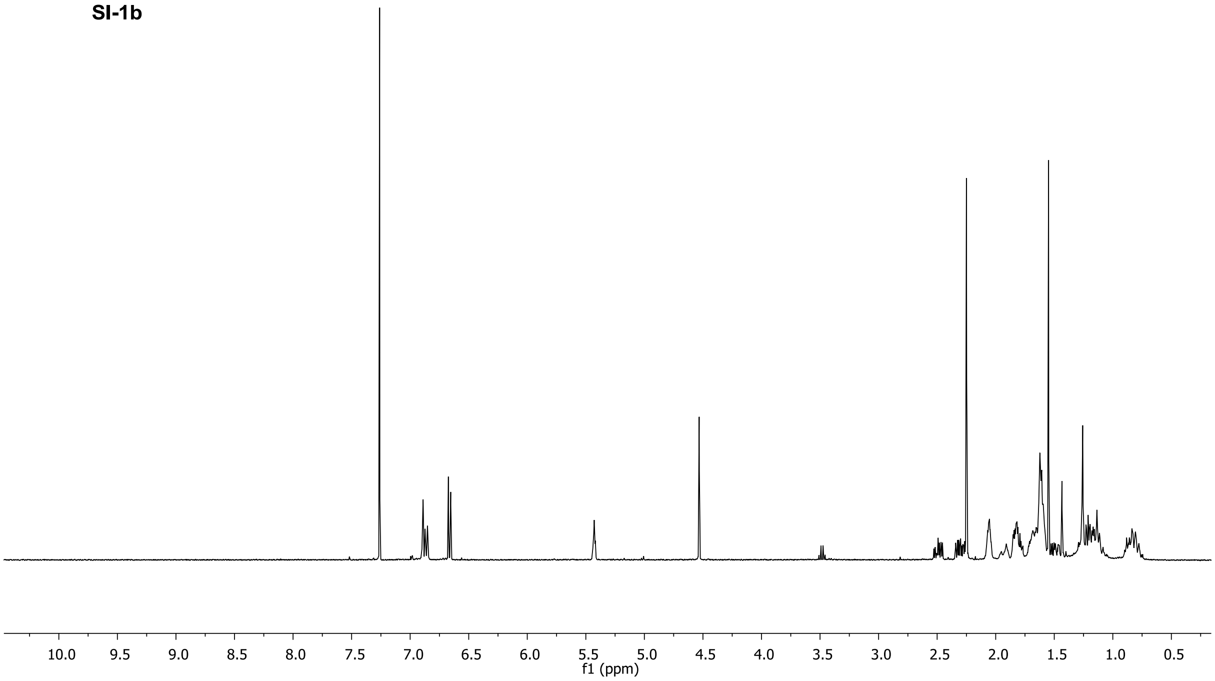

elccd023b
13C 100.6MHz Job 20041 Coutant Eloi D023B CDCl3 25.0°C 14 hours 20 min
*

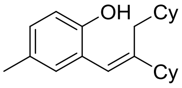

SI-1b

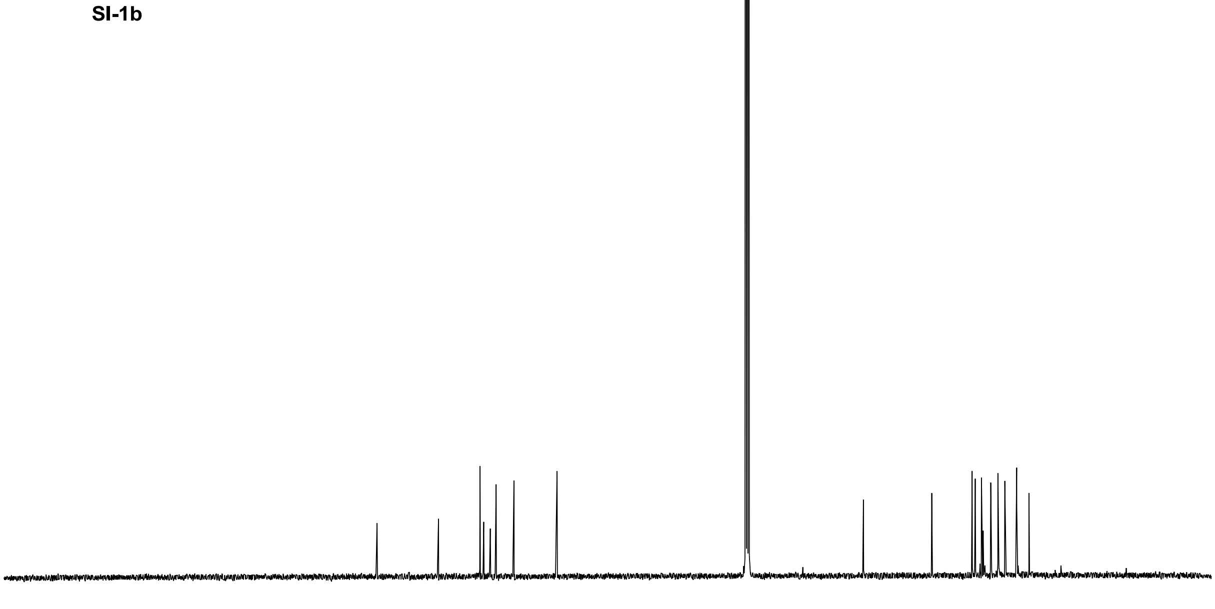

References

(1) Morrill, C.; Grubbs, R. H. *J. Am. Chem. Soc.* **2005**, *127*, 2842–2843.

(2) Young, P. C.; Schopf, N. A.; Lee, A.-L. *Chem. Commun.,* **2013**, *49*, 4262-4264.

(3) Fernandez-Mateos, A.; Madrazo, S. E.; Teijoin, P. H.; Gonzalez, R. R. *Eur. J. Org. Chem.*, **2010**, *5*, 856-861.

(4) (a) Masuda, Y.; Hoshi, M.; Arase, A. *Bull Chem. Soc. Jpn*. **1992**, *65*, 3294-3299. (b) Cain, M. E. *J. Chem. Soc.* **1964**, 3532-3535.
